# Robust and Sensitive Analysis of Mouse Knockout Phenotypes

**DOI:** 10.1371/journal.pone.0052410

**Published:** 2012-12-26

**Authors:** Natasha A. Karp, David Melvin, Sanger Mouse Genetics Project, Richard F. Mott

**Affiliations:** 1 Mouse Informatics Group, Wellcome Trust Sanger Institute, Hinxton, Cambridge, United Kingdom; 2 The Wellcome Trust Centre for Human Genetics, Oxford, United Kingdom; University of Westminster, United Kingdom

## Abstract

A significant challenge of *in-vivo* studies is the identification of phenotypes with a method that is robust and reliable. The challenge arises from practical issues that lead to experimental designs which are not ideal. Breeding issues, particularly in the presence of fertility or fecundity problems, frequently lead to data being collected in multiple batches. This problem is acute in high throughput phenotyping programs. In addition, in a high throughput environment operational issues lead to controls not being measured on the same day as knockouts. We highlight how application of traditional methods, such as a Student’s *t*-Test or a 2-way ANOVA, in these situations give flawed results and should not be used. We explore the use of mixed models using worked examples from Sanger Mouse Genome Project focusing on Dual-Energy X-Ray Absorptiometry data for the analysis of mouse knockout data and compare to a reference range approach. We show that mixed model analysis is more sensitive and less prone to artefacts allowing the discovery of subtle quantitative phenotypes essential for correlating a gene’s function to human disease. We demonstrate how a mixed model approach has the additional advantage of being able to include covariates, such as body weight, to separate effect of genotype from these covariates. This is a particular issue in knockout studies, where body weight is a common phenotype and will enhance the precision of assigning phenotypes and the subsequent selection of lines for secondary phenotyping. The use of mixed models with *in-vivo* studies has value not only in improving the quality and sensitivity of the data analysis but also ethically as a method suitable for small batches which reduces the breeding burden of a colony. This will reduce the use of animals, increase throughput, and decrease cost whilst improving the quality and depth of knowledge gained.

## Introduction

The mouse is the premier model organism for understanding gene function in development and disease. To further the functional annotation of the mammalian genome, the International Mouse Phenotyping Consortium (www.mousephenotype.org/ *)* [1] aims to phenotype knockouts for all mouse genes, building on the large collection of targeted alleles in C57BL/6N embryonic stem cells available from the International Knockout Mouse Consortium [2]–[4]. Many centres are screening mutant mouse strains to identify genes with phenotypes of interest and are making this data publicly available [1] as primary screen data with pipelines constructed to give a shallow but broad review of an animal’s phenotype. The complementary role of secondary phenotyping is to confirm and extend the primary observations into specialised fields of research. Concern over reproducibility of phenotyping experiments has been raised [5]–[7]. Some of these reproducibility issues have been tracked to the presence of environment*genetic interactions [5], [7], [8] but may be arising from poor design and analysis [9]–[11]. Poor experimental design, analysis and reporting was noted to be a significant problem in a systematic review of published papers involving *in vivo* experiments [10]. This has led to the publication of the Animal Research: Reporting *In Vivo* Experiments (ARRIVE) guidelines [12] a check list to lead the field towards good practice. This includes ensuring the analysis is appropriate for the design and data characteristics, such as the results are robust and have isolated cause and effect (high internal validity). An experiment is described as having high internal validity when the effect (e.g. phenotypic difference) can be confidently assigned to the treatment (e.g. genotype difference). To achieve high internal validity, careful experimental design is needed to account for potential confounders and the statistical test used needs to consider the structure of the data appropriately. The threats from poor control of confounding factors have been identified in many biological fields, from biomarker discovery to genome wide association studies [13], [14].

Through high throughput phenotyping programs, where data is systematically collected on one genetic background, the significant sources of variation can be identified and it has become obvious that batch (defined here as those readings collected on a particular day) can lead to large variation in phenotyping variables. This observation has significant implications for the data analysis of both high throughput and secondary phenotyping experiments where use of small batches of animals is common. It is challenging and costly to produce sufficient animals of the right age within a narrow time point for an experiment. Consider the Sanger Mouse Genetics Project which requires 7 male and 7 female homozygote mice, generated by a heterozygote cross; a best case scenario would require 14 mating pairs being assembled at the same point in time [15]. In order to generate these mating pairs, there would be a staged breeding process to generate the mice which involves several rounds of expansions depending on breeding success. This best case scenario is commonly hampered by fecundity, viability or other phenotypic problems within a line and hence to achieve a one batch pipeline the pairing number needs increasing significantly. In contrast, by accepting smaller numbers of mice in multiple batches, lower breeding pair numbers can be established. The smaller scale allows the generation of mice to answer firstly developmental and breeding issues and secondly to feed the pipeline over time and subsequent litters. As soon as we have mice of the right age, these are entered into the pipeline and have an average batch size of three for an allele per gender when we aim to phenotype 7 mice per gender for each allele. This batch approach, has allowed us to utilise animals that would otherwise be discarded as the process had not generated the required experimental sample size. Multiple small batches allow us to meet the high throughput pipeline needs and also help reduce the breeding cost per line. However, this approach will have implications on the data analysis, in the presence of temporal variation; the phenotypes of mice in the same batch are likely to be more similar than those from different batches. Furthermore, the operational constraints arising in a high throughput environment make optimal experimental design impractical; typically, mutant and control mice are not assayed on the same day, so any phenotypic differences could be due to genotype or to subtle changes in the environment (e.g. temperature fluctuations or pipetting errors). Data analysis, with the aim of controlling for variability over time [16], [17], is a major challenge for high throughput phenotyping and often a problem in secondary phenotyping.

Body weight is known to correlate with many other biologically interesting variables (e.g. bone density, blood calcium level and high- density lipoproteins) [17], [18]. Furthermore, body weight is a highly heritable trait, and consequently commonly altered in knockout lines of mice [19]. It is therefore unsurprising when the knockout also results in difference in these other variables. It raises the question as to whether the change in these variables is as expected given the observed change in body weight. Statistically, body weight in these examples is described as a confounding variable, which is one that it is associated with both the probable cause (genotype) and the outcome (phenotypic trait of interest). To understand the observed phenotype, the analysis pipeline should assess whether the change observed was due to the genotype or associated with the body weight change accompanying the genotype change.

Current analysis methods can be divided into two types; a reference range methodology (RR)(as implemented at http://www.sanger.ac.uk/mouseportal/; [20]), or the application of traditional statistical tests [21]–[23]. In RR, control mice of the same genetic background and sex are used to estimate the natural variation in a trait. In the Sanger Mouse Genetics Project, a knockout has an “abnormal phenotype” if over 60% of the mutant mice lie outside the range of 95% of the natural variation in the controls. This percentage was empirically selected to ensure the majority of mice for a line were affected. With this method, there is no p-value and the false positive and negative rates are undefined and not controlled. Traditional statistical tests, such as a Student’s *t*-Test or ANOVA, do control the false positive rate if factors such as body weight and batch do not affect the phenotype. Moreover, as the Student’s *t*-Test is the most powerful statistical test for a difference in the means of two groups with Normal errors, it should be preferred to the RR in principle. However, a more important consideration is that the traditional tests produce false positive phenotype calls if weight and batch affect the phenotype.

An alternative method, linear mixed models (MM) are a class of statistical models suited to modelling multiple sources of variability on a phenotype, where some explanatory factors (such as sex, body weight and mutant genotype) are assumed to take fixed values that affect the population mean, whilst others such as batch are treated as affecting the covariance structure; animals from the same batch will have correlated phenotypes. MM are an established technique in the analysis of complex traits (for example in maize [24] and mice [25]), but to our knowledge they have not been usedwithin the mutant mouse phenotyping community and no comparison or discussion on this method versus others has been published. The few examples we have identified, include Kafkafi *et al.* who used a MM to compare open field data for various mouse lines across institutes to assess the prevalence of genotype*environment interactions and treated the variation between laboratory as a random effect [8]; Wainwright *et al.*, in a study looking at the impact of pre-natal ethanol exposure in mice on behaviour and brain size, demonstrated the value of a MM approach where litter was treated as a random effect over an ANOVA on litter mean data [26]; Goncalves *et al.*, used a MM to query cardiovascular data where a repeated measures design had been used in mice and the subject was treated as the random effect [27].

With high throughput data, we are treating batch as the random effect adding variation to the data. The variation in batch arises from multiple factors including technician, reagent lot, day, cage, mother and litter size [17]. All these effects are modelled and tested within the MM framework. For each mutant strain, we test the contributions of sex, weight, genotype and genotype-by-sex interaction by fitting two nested mixed models (Equation [1], and Equation [2]), where the phenotype of mouse *i* is assessed within the *j*-th batch. (See Table 1 for parameter and associated definitions.) A comparison between the fits of the models tests whether the phenotype is mediated by a body weight change. The MM can be interpreted as a generalisation of the T-test that takes into account the explanatory variables, in the sense that it is almost identical to the T-test if they are not significant.
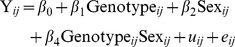
(1)

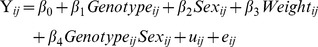
(2)


**Table 1:** Parameters and the associated definitions for equation 1 and 2.

Parameter	Definition
Y	The dependent variable (e.g. the variable of interest)
*β*_o_	The expected value of Variable Y for the reference levels of genotype (wildtype) and sex (female) when weight equals zero if weight included.
*β*_1_	The effect of genotype (i.e. knockout (homozygote or heterozygote) versus. wildtype)
*β*_2_	The effect of sex (female versus. male)
*β*_3_	The effect of body weight (measured in grams).
*β*_4_	The fixed effect associated with the genotype by sex interaction
*u*	The random effect associated with the intercept for batch. The distribution of these random effects are U_j_ ∼ N(0, σ^2^)
*e*	The distribution of the residual

For smaller scale projects, such as in secondary phenotyping, where the batch number is limited, there is an potential alternative to the MM of treating batch as a fixed effect rather than a random effect and then using a generalised linear model. As the measured batches are a random subsets of all possible batches, treating it as a random variable in a MM allows us to reflect the random selection of batch and thus is theoretically more appropriate. This can be seen in that the user is typically not interested in batch i.e. what was the result on Wednesday and how did it differ exactly from Monday etc. The primary need is to account for batch in the analysis. Furthermore, the MM will be more sensitive, as it economises on the number of degrees of freedom used by the factor levels; instead of estimating a mean for every single factor level, the random effect model estimates the distribution of the mean [28].

To assess these approaches, we investigated control data to assess the temporal variation visualised with batch. We then considered the applicability of the various analysis methods in the presence of multiple batches. To demonstrate the issues we analysed four randomly-selected mutant colonies (*Ppp3ca^tm2e(EUCOMM)Wtsi^*, *Sparc^tm1a(EUCOMM)Wtsi^*, *Cenpj^tm1a(EUCOMM)Wtsi^*, and *Slc25a21^tm1a(KOMP)Wtsi^*) from the Sanger Mouse Genetics Project with a focus on seven traits from the Dual-Energy X-Ray Absorptiometry (DEXA) screen [29] which focuses on bone and tissue composition. Here we show that a linear mixed model is an appropriate method to query data which has a batch issue. We also show how this approach detects subtle but important quantitative differences in phenotype that are currently overlooked. This manuscript intends to demonstrate that this method is a significant improvement over methods currently applied in identifying phenotypes and has ethical benefits.

## Materials and Methods

### Ethics Statement

The care and use of all mice in this study was carried out in accordance with UK Home Office regulations, UK Animals (Scientific Procedures) Act of 1986 under two UK Home Office licences (80/2076 and 80/2485) which were reviewed yearly by the Wellcome Trust Sanger Institute’s Ethical Review Committee.!.

### Mice

Mice were maintained in a specific pathogen free unit on a 12 hr light: 12 hr dark cycle with lights off at 7∶30 pm and no twilight period. The ambient temperature was 21±2°C and the humidity was 55±10%. Mice were housed for phenotyping using a stocking density of 3–5 mice per cage (overall dimensions of caging: (L×W×H) 365×207×140 mm, floor area 530 cm^2^) in individually ventilated caging (Tecniplast Seal Safe1284L) receiving 60 air changes per hour. In addition to Aspen bedding substrate, standard environmental enrichment of two nestlets, a cardboard Fun Tunnel and three wooden chew blocks was provided. Mice were given water and diet *ad libitum*. At 4 weeks of age, mice were transferred from Mouse Breeders Diet (Lab Diets, 5021-3) to a high fat (21.4% fat by crude content; 42% calories provided by fat) dietary challenge (Special Diet Services, Western RD 829100).

### Dual-energy X-ray Absorptiometry (DEXA) Measurements

At 14 weeks of age the mice were weighed and then anaesthetized with Ketamine (100 mg/kg, Ketaset, Fort Dodge Animal Health) and Xylazine (10 mg/kg, Rompun, Bayer Animal Health). Body length (nose to tail base (cm)) was measured and dual-energy X-ray imaging performed using a PIXImus II Bone Densitometer (GE Medical Systems, United Kingdom). The region of interest on the resulting images was manually selected to exclude the skull, and then the Lunar PIXImus software package calculated body fat mass (g), lean mass (g), fat percentage estimate (%), bone area (cm^2^), bone mineral density (BMD) (g/cm^2^) and bone mineral content (BMC) (g). Quality control using a phantom mouse was performed prior to imaging. The Ketamine/Xylazine anaesthesia was reversed using Atipamezole hydrochloride (1 mg/kg, Antisedan, Pfizer Animal Health). Cages were processed randomly and different genotypes could be housed together, hence there was no pattern to the order in which animals were processed. When mice were fully ambulant, they were housed in their group cages and kept for further experiments.

### Other Phenotypic Screens

Data from the MGP pipeline is obtained following the standard SOP available at https://www.mousephenotype.org/impress.

### Datasets

To assess the significance of batch and weight to variability of a trait, B6Brd;B6N-*Tyr^c-Brd^* wildtype data from the MGP pipeline from 2009 until 2012 was used. This gave a large dataset (average size: 1700 measurements from 170 independent batches) for 88 quantitative traits.

To compare the different analysis methods, data from *Ppp3ca^tm2e(EUCOMM)Wtsi^*, *Sparc^tm1a(EUCOMM)Wtsi^*, *Cenpj^tm1a(EUCOMM)Wtsi^*, and *Slc25a21^tm1a(KOMP)Wtsi^* were obtained from the Mouse Genetic Project running at the Wellcome Trust Sanger Institute. Three of these were randomly selected from a subset of genes which had one or more hits in DEXA as assessed by the RR, whilst one was randomly selected from a subset of genes which had no hit in DEXA as assessed by the RR. Within the pipeline, we screen a single control cohort (7 males and 7 females) each week, matching the genetic background of the mutant lines. We selected control mice run in the same pipeline on the same genetic background contemporaneously with the corresponding knockout mice (Table 2). From the website http://www.sanger.ac.uk/mouseportal/, data for each gene can be viewed or the raw data can be downloaded using the MGP Phenotyping BioMart http://www.sanger.ac.uk/htgt/biomart/martview/).

**Table 2:** For each allele the composition of the datasets.

Allele	Genetic background	Homozygotes				Controls			
		Male		Female		Male		Female	
		N	B	N	B	N	B	N	B
*Ppp3ca^tm2e(EUCOMM)Wtsi^*	B6Brd;B6N-*Tyr^c-Brd^*	7	4	7	4	271	39	287	42
*Slc25a21^tm1a(KOMP)Wtsi^*	B6Brd;B6N-*Tyr^c-Brd^*	7	2	7	3	38	9	40	10
*Cenpj^tm1a(EUCOMM)Wtsi^*	B6Brd;B6N-*Tyr^c-Brd^*	8	3	7	3	198	56	200	31
*Sparc^tm1a(EUCOMM)Wtsi^*	B6Brd;B6N-*Tyr^c-Brd^*	9	2	7	3	223	40	229	42

N refers to the total number of mice for that dataset and B refers to the number of batches of mice within that dataset, where a batch is a group of mice measured on the same day.

### Data Analysis

Mixed model data analysis was performed using R (package: nlme version 3.1) [30] ( File S1 gives the R code used). An iterative top down modelling strategy was implemented with six steps [31] starting with the most comprehensive model (either Eq. [1] or [2]). First the analysis selects a structure for the random effects, then a covariance structure for the residual, then the model is reduced by removing non-significant fixed effects, finally the genotype effect is tested and model diagnostics visualised. During the model building stage, the hypotheses were tested with a threshold of p<0.05. For the hypothesis test of primary interest, the impact of genotype, p-values were adjusted to account for the multiple comparisons completed to control the false discovery rate to 0.05 (R function: p.adjust).

The reference range methodology is implemented within the Sanger Laboratory Information Management System (LIMS) database that houses the data captured from the high throughput pipeline. The reference range for each variable was built using data from all control mice with the same genetic background, age and sex which were collected using the same standard operating procedure. This is intended to build a reference range that encompass all sources of variation seen in the pipeline, such as phenotyper or batch, hence if a mutant is outside this reference range the difference is likely to have arisen from the genotype difference. For each mutant line and for each sex an abnormal phenotype was highlighted if ≥60% of the mutant mice lie either above or below the 95% confidence interval of the reference range. The 95% confidence interval is taken as the 2.5 and 97.5 percentile for each variable. Using percentile avoids any distribution assumptions thereby increasing precision of the methodology. In addition to the automatic identification of abnormality using the above rule, a manual assessment was made by biological experts, who use knowledge of events on the day or across phenotypic variables or sex to make a call. Any call of abnormality, whether manual or automated, was compared to the call of significance made by the mixed model methodology.

## Results

### Is Batch Variability a Significant Issue?

To assess the significance of batch variability in phenotyping of quantitative variables, the proportion of variance arising from batch relative to sex and weight (when applicable) were estimated for 88 phenotypic traits. Linear regression models, which included the covariates of interest, were fitting towildtype data from the same genetic background (B6Brd;B6N-*Tyr^c-Brd^)*, age and gender (typically dataset size: 1700 measurements from 170 independent batches) and then the proportion of variance explained by the covariate relative to the total variance calculated (Table S1). On average 22.3±1.5% (standard error of the mean) of the total variance arose from batch, 11.9±1.8% (standard error of the mean) arose from sex and 6.3±1.2% (standard error of the mean) arose from body weight variation. This analysis finds that batch explains about a quarter of the observed phenotypic variation, and for some phenotypes was comparable to or more significant than the effects of sex and weight and hence we can conclude that batch is a significant factor in the variability of phenotyping data.

### Evaluating Appropriateness of the Various Statistical Approaches

The use of traditional statistical tests, RR and MM in identifying significant phenotypes are discussed in detail and Table 3 summaries the differences.

**Table 3:** Comparison of the analysis methods used for identifying phenodeviants.

	Traditional method	RR	MM
Sensitivity?	✓	X	✓
Does output include a *p* value which will allow multiple testing correction?	✓	X	✓
Can the method account for batch?	X	X	✓
Can the method include additional variables, e.g. weight, in presence of batch?	X	X	✓
Are the calls reliable in presence of batch?	X	✓	✓

The classification traditional method refers to the use of classic inferential statistical methods typically used e.g. student *t*-Test, RR refers to a reference range, and MM to a mixed model approach.

### Reference Range

The RR relies on establishing the natural variation in a phenotype. To establish a RR, a minimum of 120 data points are needed [32] and the resulting thresholds are specific to a laboratory and its procedures [33]. This can limit its use in small scale phenotyping projects. If we make the simplifying assumption that the 95% range is estimated perfectly and that no other factors (such as batch or weight) need to be accounted for, then the probability that a given number of the N tested mutants lie outside the 95% range is distributed as a binomial random variable B(N,0.05). Thus for the Sanger Mouse Genetics Project, where we usually phenotype N = 7 mutant mice per sex and require 60% of the mice to lie outside the reference range, the probability of observing at least 5/7 extreme phenotypes by chance (the false positive rate) is about 6×10^−6^ per sex per tested phenotype, and the chance that either sex is significant is about 1.2×10^−5^ per phenotype. This probability increases significantly if fewer mutant mice are tested. Thus RR is stringent but it is not a well-controlled significance test and could be considered a qualitative test. This can be seen in the output, an abnormal phenotype is either identified or not, there is no calculated p-value. Without a measure of the false positive rate, there is no possible correction for multiple testing. Furthermore, the reference range has no capacity to include additional covariates, such as a body weight, to separate the genotype effect from changes in these covariates.

### Traditional Statistical Methods

With phenotyping data, traditional statistical tests, such as a Student’s *t*-Test or a Mann-Whitney test are commonly used to compare control and treated data whilst a 2-way ANOVA allows the comparison of control and treated data for both genders simultaneously. These tests make a number of mathematical assumptions; providing these are met then these tests are sensitive and generate p-values that can be used with multiple testing correction. If these assumptions are not met then the p-values will not be reliable. One assumption in common with the traditional approaches is that the measurements are independent measurements from a single population and the errors will thus be independent. With significant temporal variation, the presence of multiple batches invalidates this assumption and the correlation can lead to an inflated estimate of statistical significance and thus false positive calls (type one errors) [34].

The commonly used statistically tests have no capacity to include additional covariates, such as a body weight, to separate the genotype effect from changes in these covariates. However, there are traditional statistical tests such as an analysis of covariance (ANCOVA) that can incorporate a covariate such as weight [18]. These tests still make the assumption that the readings are independent so if the experiment is designed to avoid these issues then an ANCOVA is an approach that could be used.

The other problem, common in high throughput phenotyping, is where controls are not measured on the same day as the knockouts, either due to practical constraints or breeding constraints, which leads to batch confounding the experiment. As batch is known to vary, then it confounds as it correlates with both the trait of interest and the treatment. In this situation, it is difficult to distinguish whether the differences in treatment groups are due to the treatment or the confounding factor. When traditional statistical tests are applied, the user is making the assumption that the experiment has been designed to manage all potential confounders and hence the cause and effect has been isolated. With high throughput data, it is not always possible to avoid batch issues and hence we do not consider the Student’s *t*-Test or Mann-Whitney Test as appropriate tools and are not considered further within this manuscript.

### Mixed Models

**Figure 1:**
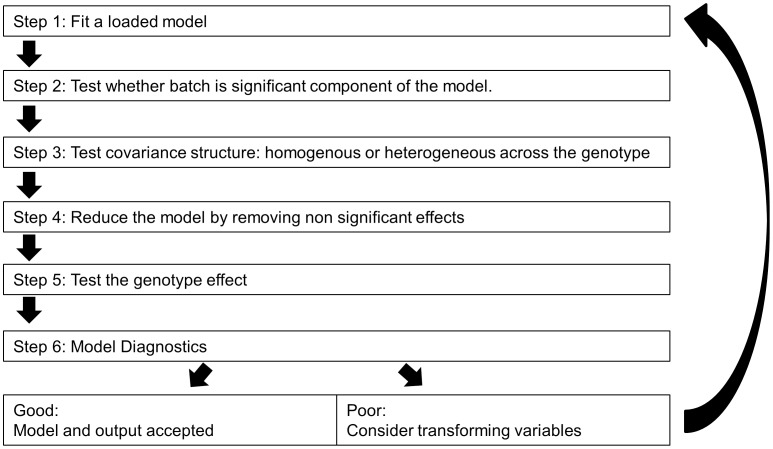
An overview of the mixed method methodology implemented. The process can be summarised as a top down methodology involving six steps to build a mixed model to query the phenotyping data.

**Figure 2:**
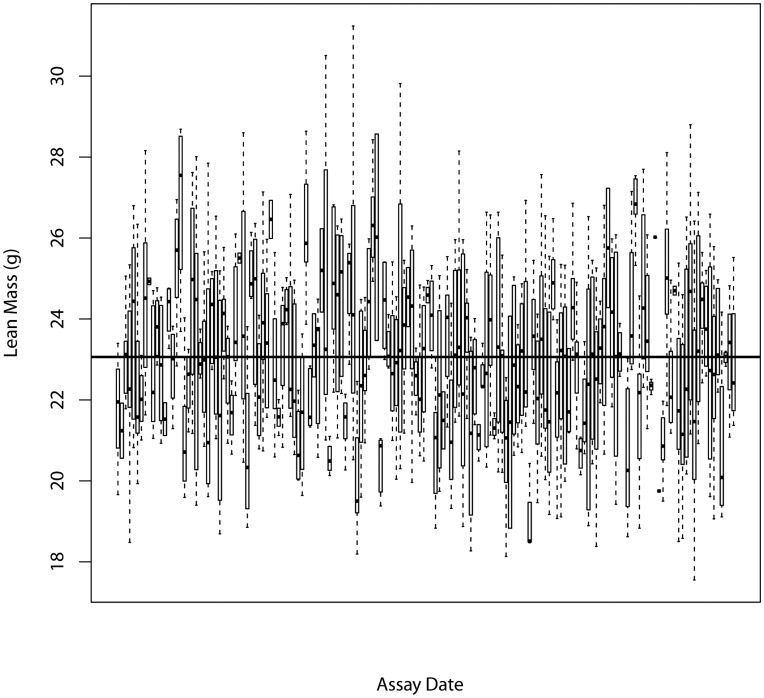
Examining control data to assess batch to batch variation. Representative time course plot showing the batch to batch variation in control data for male mice from a B6Brd;B6N-*Tyr^c-Brd^* genetic background. Example shown is the variation seen in the fat mass variable measured in grams. For each day, data was collected a box plot is drawn as a five point summary indicating the minimum, 1^st^ quartile, median, 3^rd^ quartile and maximum. The global median fat mass value is shown with a black solid line.

**Figure 3:**
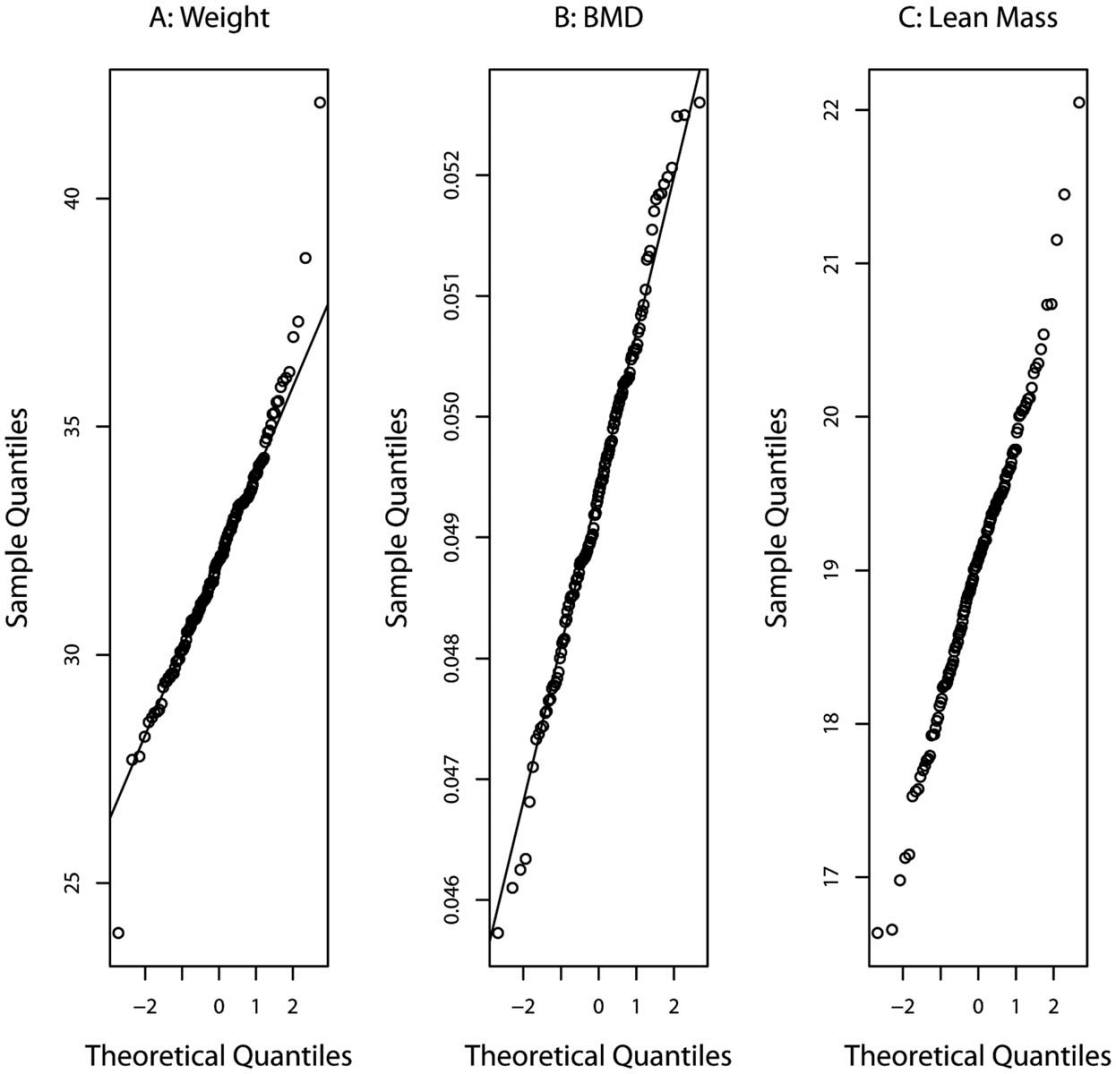
Assessing distribution of batch for a variable. Representative Normal Q-Q plots of the distribution of the mean for a batch in control data for male mice from a B6Brd;B6N-*Tyr^c-Brd^* genetic background. A: weight, B: bone mineral density and C: lean mass.

**Figure 4:**
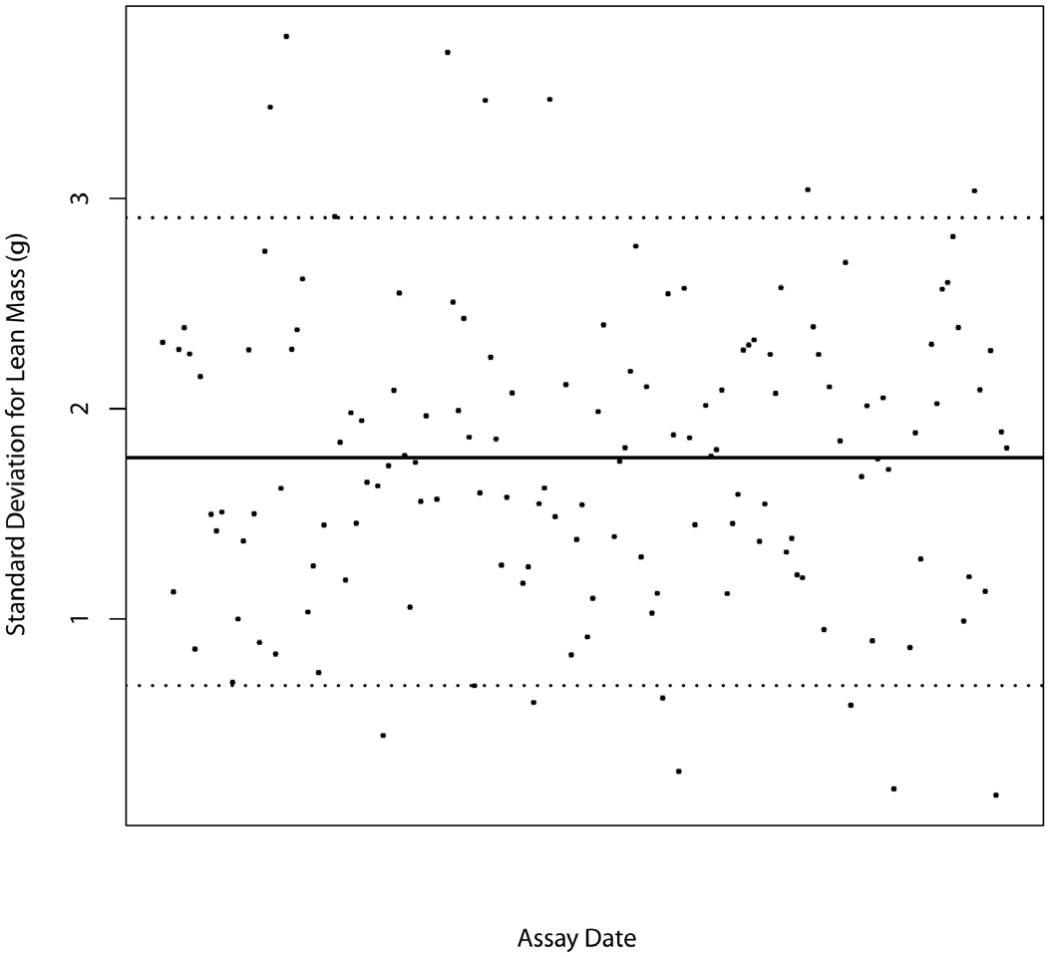
Examining control data to assess variation in standard deviation with batch. Representative time course plot showing the variation in standard deviation with batch in control data for mice from a B6Brd;B6N-*Tyr^c-Brd^* genetic background. Example shown is the variation seen in the lean mass variable for male mice measured in grams. The global median is shown with a black solid line, and the 95% confidence interval is shown with dotted lines.

Mixed models can be applied in multiple ways, and we have implemented a top-down methodology which starts with a fully loaded model (Eq [1] or Eq [2]) and steps through an iterative process to fit the best model to the data prior to asking whether genotype is significant or not (Figure 1) [31]. The mixed model makes a number of assumptions about the data (Table 4). To test whether these assumptions are valid, control data was explored with a variety of tools. To assess the assumptions associated with batch, a boxplot of control data from the B6Brd;B6N-*Tyr^c-Brd^* genetic background against time illustrates its temporal distribution. The distribution resembles samples drawn from a stationary Normal distribution with a common variance (Figure 2). Normal Q-Q plots of the distribution of the batch mean for each variable showed the means were normally distributed (Figure 3). A time course plot showed that variation in the standard deviation for an individual batch was randomly distributed around a mean, with a few outliers which tend to be small, which indicates the assumption of homogeneity of variance could be made (Figure 4). A runs test for randomness was used to test for autocorrelation, and for all variables no correlation was found in the distribution of the mean signal for batch (Table S2), suggesting that batch was independently distributed. This exploration indicates that the underlying assumptions for batch were appropriate.

**Table 4:** Assumptions associated with the mixed model.

Parameter	Assumption
Batch *u*	• Independent identically distributed N(0, σ_u_^2^)
Error *e*	• Independent identically distributed N(0,σ_e_^2^)
Covariate – weight* ^1^	• Linear relationship with dependent variable
	• Homogeneity of regression

* 1 when weight included in the model as a covariate (Eq. [2]).

To test the quality of model fit, a number of graphical diagnostic plots were generated for each gene and trait (File S2, S3, S4, S5). To test the assumption of normality, a normal Q-Q plot was used to compare the variable distribution with a normal distribution. If the variable was normally distributed it would be randomly distributed around the equivalence line on the graph. A normal Q-Q plot was used to test the distribution of the best linear unbiased prediction of random effects (BLUPS) of the final model (provided a mixed model was appropriate after the five step model fitting process) and this checked the assumption that batch was normally distributed. The assumptions associated with error, of a Normal distribution with a common variance, can be assessed using a variety of graphical plots. A Normal Q-Q plot was also used to assess whether the conditional raw residuals had a Normal distribution for each treatment group. A plot of conditional raw residual versus predicted values for each treatment group was used to assess whether the model fitted each treatment group equally well and no dependencies on the range were seen. When body weight was included in the model, the assumptions associated with body weight were assessed with a body weight versus variable scatter plot for each dependent variable examined. Taken together, these diagnostic plots all suggest the mixed models were a good fit to the data.

The MM approach not only generates p-values but a precise estimate of genotype effect. With this p-value, multiple testing correction methods can be applied. We chose a relatively simple set of explanatory variables to model the data based on prior knowledge. The law of parsimony argues that, simpler explanations are, other things being equal, generally better than more complex ones. Moreover, with only 7 male and 7 female readings for the knockout lines we were limited on the number of variables we couldninclude in the model. A large-scale study of heterogeneous mice identified numerous covariates (e.g. age, cage density, litter, and weight) that were significant effects in the variation of phenotypic characteristics [17]. Many of the variables identified in that study are controlled within this pipeline (e.g. age), and thus are not relevant. Batch, we anticipate is a composite effect arising from cage density, parental origin, technician and assay date. With our limited data, we cannot decompose it further and need a model that is a pragmatic compromise. We investigated using litter membership as an alternative to batch in the mixed model, but whilst the genotype estimates had the same precision and accuracy, the model diagnostics were poor (data not shown). With larger datasets, greater sensitivity can be obtained and additional covariates could be considered to increase the precision of the estimates.

### Comparison of RR and MM Approach

To compare the value of the MM and RR methodologies, we analysed four randomly-selected mutant colonies (*Ppp3ca^tm2e(EUCOMM)Wtsi,^ Slc25a21^tm1a(KOMP)Wtsi,^ Cenpj^tm1a(EUCOMM)Wtsi^ and Sparc^tm1a(EUCOMM)Wtsi^*), from the Sanger Mouse Genetics Project with a focus on seven traits from the Dual-energy X-ray absorptiometry (DEXA) screen [29] and compared the output of the MM with the output of the RR (Files S2, S3, S4, S5 give detailed output for each gene). A comparison of the genotype calls made for the four genes by the two methods is in Table 5.

**Table 5:** A comparison of phenotypic calls.

Allele	Phenotypes	RR	MM	
			Eq.1	Eq.2
*Ppp3ca*	Weight	–	↑	NA
	Nose to tail length	–	–	–
	Bone mineral density	–	–	–
	Bone mineral content	–	↑	–
	Lean mass	–	↑	–
	Fat mass	–	↑	–
	Fat percentage	–	↑	–
*Slc25a21*	Weight	↓	↓	NA
	Nose to tail length	–	↓	–
	Bone mineral density	↓ ♀	↓	–
	Bone mineral content	↓ ♀	↓	–
	Lean mass	–	↓	–
	Fat mass	↓	↓	–
	Fat percentage	↓ ♂	↓	–
*Sparc*	Weight	–	↓	NA
	Nose to tail length	–	↓	↓
	Bone mineral density	↓♀	↓	↓
	Bone mineral content	↓♀	↓	↓
	Lean mass	–	↓	–
	Fat mass	–	↓	–
	Fat percentage	–	–	–
*Cenpj*	Weight	↓	↓	NA
	Nose to tail length	↓	↓	↓
	Bone mineral density	–	↓	–
	Bone mineral content	–	↓	↓
	Lean mass	↓	↓	↓
	Fat mass	↓	↓	↑
	Fat percentage	–	↓	↑

A comparison of calls between the reference range (RR) and mixed model (MM) methodologies for four mutant colonies for genotype calls of significance. Where a call of significance is made, (↑) indicates that the genotype effect gave an increase in the variable, whilst (↓) indicates a decrease. A dash indicates that genotype was not significant. NA is used to indicate that weight as the variable of interest cannot be fitted with Eq.2 as this includes weight as a covariate. For the RR, if a call arises from only one sex, the standard gender symbols are used to indicate which. Significant MM calls were controlled to have a false discovery rate of 0.05.

Since the RR analysis cannot take into account changes in body weight, we first compared it to the MM equation [1]. MM showed an apparent increase in sensitivity over RR, calling 93% of phenotypes as significant at a false discovery rate (FDR) of 5%, compared to 39% with RR. As expected, the RR calls were a subset of the MM calls, at first sight reflecting the RR’s greater stringency. However, most of the RR calls were due to body weight effects; using the MM equation [2], which tests whether a mutant affects a trait after accounting for sex and body weight, only 33% of traits were called at FDR 5%. Importantly, some RR calls were no longer significant, some negative RR calls became significant, and some positive calls reversed direction of effect. Thus the difference between the MM and RR is not simply a question of differing false positive rates.

MM analyses of *Ppp3ca* and *Slc25a21* showed these mice have a statistically significant weight phenotype and when weight is excluded from the model (Eq. 1) many of the body composition variables were statistically significant. However, application of a MM with weight included in the model finds that these body composition changes were as expected, as these changes are in line with the change in body weight observed with this mutation (Table 5). *Sparc* is a mouse model for human osteoporosis [35], and the MM excluding weight (Eq. 1) called multiple phenotypes whilst the RR only called a decrease in bone mineralisation in female mice (Table 5). By including weight in the MM we found that the changes in length, fat mass, and lean mass were as expected with the reduced weight, whilst the changes in bone mineralisation were actually greater than expected. The remaining example, *Cenpj* which has a reduced body weight phenotype, highlights an interesting effect of adjusting for weight: fat mass and fat percentage were significantly lower in mutants relative to controls (FDR 5%), but after adjusting for weight these variables had increased relative to controls as their change was less than expected given the decrease in weight. These results demonstrate the value of being able to compare the call of significance with and without weight included in the model to understand the observed phenotype.

The example above, with a focus on DEXA variables, includes variables which strongly correlate to body weight and hence there was clear value in including body weight in the MM. We have found that weight is a significant variable in many areas, and as body weight is a common phenotype it is valuable to consider all phenotypic traits with a MM including and then excluding body weight to assess whether the effect was mediated by body weight. For example, *Slc25a21* had 3 clinical chemistry variables (alanine aminotransferase, albumin and aspartate aminotransferase) identified by the RR as being abnormal. Application of the mixed model approach with both Eq.1 and Eq.2, identifies that whilst these variables are statistically significantly lower in the knockout compared to the wildtype mice as tested for with Eq.1, once weight is included in the model (Eq.2) they are no longer statistically significant. We therefore conclude that the difference in these variables was mediated by the body weight change (Table S3).

## Discussion and Conclusions

The manuscript demonstrates that batch is a significant source of variability for continuous phenotypic traits and without management can be a confounding factor preventing the isolation of the effect of interest (e.g. genotype). The use of traditional statistical methods, with batch confounding the experiment, will lead to flawed conclusions and could go some way to explain the concerns of phenotypic reproducibility where a phenotype could not be reproduced in an independent laboratory or related to a human disease. As a consequence, either the experiment should be designed to avoid batch issues or an analysis method, such as the MM, which is appropriate for datasets with multiple batches, should be used.

Embracing a workflow with multiple small batches was an essential component of realizing a high throughput pipeline and allowing production of 160 lines/year. However, breeding issues and hence batch issues are not restricted to high throughput programs. For small scale phenotyping experiments, resources can be applied to ensure the traditional methods are appropriate by randomising appropriately or the use of a one batch approach. We would argue that the use of a MM methodology has value for large and small scale experiments, not only in terms of resources, and thus cost, but ethically as less breeding is needed to achieve the same goal. A one-batch approach could be argued to be a form of standardisation and thus reduce the variance and increase sensitivity. However, multiple papers have argued that identifying phenotypes in overly standardised environments results in false calls of significance and that there is value in a design that encompasses variation as this increases the external validity (i.e. the generality of the finding) and makes the finding more reproducible [6], [7], [36].

In the presence of multiple batches and asynchronous controls, both the RR and MM make phenotypic calls with high internal validity, however, we conclude that applying MM to high throughput phenotyping data improves phenotype calling. Compared to the RR, MM is more sensitive, controls for power and lets us separate out the effects of bodyweight, sex and batch. The MM approach brings significant value, which merits the additional analysis complexity. The ability to separate effects of covariates, particularly weight, will significantly improve the precision in the identification of targets for secondary phenotyping. For example, consider the identification of bone mineralisation phenotypes, with body weight being a common phenotype and bone mineral density correlating with body weight; up to 35% of the knockout lines will automatically have a bone density phenotype unless the call is adjusted for weight. This was seen in the four alleles considered in detail within this manuscript. Until weight was considered in the equation all four had a significant bone mineral density phenotype, but after including weight as a covariate we would only call *Sparc* as phenodeviant. The greater sensitivity of the MM allows the detection of more subtle phenotypic abnormalities than the traditional methodologies. This will lead to an increase in both the phenotype annotations for each mouse line and the ability to use the results to further our understanding of a gene’s basic function and roles in human disease. In particular, computational approaches that look to identify lines as models for particular human diseases [37] will benefit massively as similar diseases that share some common clinical phenotypes can be distinguished on the basis of these extra annotations. Given the costs of mouse production and phenotyping, maximising the scientific knowledge gained through optimal use of statistical analysis is especially important. Whilst MM has significant advantages, there is value in maintaining both analysis pipelines as the RR is well established within the community, easy to access and interpret, and is ideal for the identification of large robust phenotypic changes.

Ensuring design and analysis match is an essential step to address the short coming identified in the Kilkenny review [10]. This manuscript demonstrates that currently methodologies used in high throughput phenotyping environments are lacking and that MM gives important improvements. These improvements will allow the benefits of these centralised programs to be fully realised, as subtle reproducible phenotypes can now be identified. We plan to apply MM to our historic data and with the greater sensitivity enhance the knowledge gained from those experiments. Looking to the future, we expect further refinement of the method as we apply the data globally within Sanger Mouse Genetics Project and across institutes via the International Mouse Phenotyping Consortium Statistics working group. The challenge will arise from balancing the needs of a common statistics pipeline to many variables and data from many sources. Further expansion of the mixed modelling technique will allow meta-analysis across genes and centres.

## Supporting Information

Table S1 Impact of sex, weight and batch on a variety of variables. Legend: The proportion of variance explained by sex, weight and batch for a variety of phenotypic traits in control B6Brd;B6N-*Tyr^c-Brd^* mice from the Mouse GP pipeline.
(DOCX)

Table S2 Temporal effects on batch means in controls. Legend: A runs test statistics of the mean of each control batch in a time series from the B6Brd;B6N-*Tyr^c-Brd^* for each sex from the Mouse GP pipeline. There was no evidence for autocorrelation after adjusting for multiple testing (method: Bonferroni).
(DOCX)

Table S3 A comparison of phenotypic calls. Legend: A comparison between genotype calls of significance when the mixed model excludes (Eq.1) or includes weight (Eq.2). Presented are body weight and three clinical chemistry variables for *Slc25a21^tm1a(KOMP)Wtsi^* on the B6Brd;B6N-*Tyr^c-Brd^* genetic background. Where a call of significance is made, (↑) indicates that the genotype effect gave an increase in the variable, whilst (↓) indicates a decrease. NA is used to indicate that weight as the variable of interest cannot be fitted with Eq.2 as this includes weight as a covariate. A dash indicates that genotype was not significant. Significant MM calls were controlled to have a false discovery rate of 0.05.
(DOCX)

File S1 The mixed model implementation code.
(TXT)

File S2 Detailed mixed model output for the allele *Ppp3ca^tm2e(EUCOMM)Wtsi^* and associated DEXA data. Legend: For each trait studied, for each model fitting procedures, the final model output was captured and the data visualised with a boxplot. Furthermore, to test the quality of model fit, a number of graphical diagnostic plots were generated for each gene and trait.
(PDF)

File S3 Detailed mixed model output for the allele *Slc25a21^tm1a(KOMP)Wtsi^* and associated DEXA data. Legend: For each trait studied, for each model fitting procedures, the final model output was captured and the data visualised with a boxplot. Furthermore, to test the quality of model fit, a number of graphical diagnostic plots were generated for each gene and trait.
(PDF)

File S4 Detailed mixed model output for the allele *Cenpj^tm1a(EUCOMM)Wtsi^* and associated DEXA data. Legend: For each trait studied, for each model fitting procedures, the final model output was captured and the data visualised with a boxplot. Furthermore, to test the quality of model fit, a number of graphical diagnostic plots were generated for each gene and trait.
(PDF)

File S5 Detailed mixed model output for the allele *Sparc^tm1a(EUCOMM)Wtsi^* and associated DEXA data. Legend: For each trait studied, for each model fitting procedures, the final model output was captured and the data visualised with a boxplot. Furthermore, to test the quality of model fit, a number of graphical diagnostic plots were generated for each gene and trait.
(PDF)
