# Supplementary material for: Robust and Sensitive Analysis of Mouse Knockout Phenotypes
Source: PLoS One. 2012 Dec 26;7(12):e52410. doi: 10.1371/journal.pone.0052410 (PMC3530558; doi:10.1371/journal.pone.0052410)
Supplement: Table S1 — Impact of sex, weight and batch on a variety of variables. Legend: The proportion of variance explained by sex, weight and batch for a variety of phenotypic traits in control B6Brd;B6N-Tyrc-Brd mice from the Mouse GP pipeline. (DOCX) [file pone.0052410.s001.docx]

Supplementary Table 1: Impact of sex, weight and batch on a variety of variables.

| Screen | Variable | Percentage variance explained by variable (%) | | |
| --- | --- | --- | --- | --- |
|  |  | Sex | Weight | Batch |
| Body composition (DEXA) | Bone mineral Content | 28.78 | 24.71 | 8.48 |
| Body composition (DEXA) | Bone mineral Density | 8.20 | 8.31 | 27.64 |
| Body composition (DEXA) | Fat percentage | 0.00 | 34.23 | 18.51 |
| Body composition (DEXA) | Fat Mass | 16.86 | 59.41 | 7.20 |
| Body composition (DEXA) | Nose to tail length | 26.79 | 18.38 | 30.22 |
| Body composition (DEXA) | Lean Mass | 54.90 | 16.09 | 8.37 |
| Body temperature | Stress Induced Hyperthermia Change in Temperature | 26.46 |  | 11.48 |
| Body temperature | Stress Induced Hyperthermia Post Stress Temperature | 0.89 |  | 27.29 |
| Body temperature | Stress Induced Hyperthermia Basal Body Temperature | 23.78 |  | 16.01 |
| Grip Strength | All paws 1 | 3.70 | 3.06 | 28.40 |
| Grip Strength | All paws 2 | 2.48 | 2.85 | 28.43 |
| Grip Strength | All paws 3 | 2.96 | 2.26 | 30.49 |
| Grip Strength | Fore paws 3 | 1.12 | 1.67 | 20.80 |
| Grip Strength | Fore paws 1 | 4.43 | 1.49 | 21.48 |
| Grip Strength | Fore paws 2 | 3.04 | 1.57 | 22.24 |
| Haematology (CBC) | Haematocrit | 11.41 |  | 31.30 |
| Haematology (CBC) | Haemoglobin | 1.62 |  | 35.11 |
| Haematology (CBC) | Mean corpuscular haemoglobin | 6.78 |  | 49.48 |
| Haematology (CBC) | Mean corpuscular haemglobin conc. | 6.24 |  | 60.26 |
| Haematology (CBC) | Mean corpuscular volume | 0.04 |  | 65.11 |
| Haematology (CBC) | Mean platelet volume | 0.10 |  | 54.73 |
| Haematology (CBC) | Platelet count | 6.09 |  | 19.31 |
| Haematology (CBC) | Red blood cells | 14.60 |  | 17.44 |
| Haematology (CBC) | Red blood cell distribution width | 0.05 |  | 62.34 |
| Haematology (CBC) | White blood cells | 22.27 |  | 16.70 |
| Heart weight | Heart weight | 22.58 |  | 74.84 |
| Hot Plate | Latency to respond | 0.36 | 0.02 | 30.16 |
| Open Field | Distance moved in centre - total | 21.98 | 4.50 | 10.50 |
| Open Field | Average speed at centre | 22.69 | 0.31 | 10.44 |
| Open Field | Time spent in centre - total | 7.42 | 2.94 | 13.25 |
| Open Field | Centre total resting | 1.33 | 0.51 | 12.19 |
| Open Field | Total distance travelled - pheriphery | 33.12 | 1.46 | 11.14 |
| Open Field | Latency to enter centre | 3.98 | 0.94 | 13.43 |
| Open Field | Number entries to centre | 30.67 | 4.47 | 10.66 |
| Open Field | Total rears | 10.11 | 0.46 | 15.63 |
| Open Field | total rest time at periphery | 6.06 | 5.36 | 14.63 |
| Open Field | Average speed at periphery | 56.34 | 1.38 | 7.41 |
| Open Field | Total time at periphery | 7.42 | 2.94 | 13.25 |
| Open Field | Total time in centre | 7.42 | 2.94 | 13.25 |
| Open Field | Whole arena total distance | 38.15 | 3.44 | 9.16 |
| Open Field | Whole arena resting time | 4.41 | 4.52 | 15.25 |
| Open Field | Whole arena average speed | 53.33 | 1.80 | 7.25 |
| Peripheral blood (FACS) | Total B cell | 7.47 | 0.09 | 14.85 |
| Peripheral blood (FACS) | T Activated T helper | 3.24 | 0.27 | 52.85 |
| Peripheral blood (FACS) | Activated  T cytotoxic | 0.00 | 1.68 | 49.69 |
| Peripheral blood (FACS) | Granulocyte | 0.20 | 0.34 | 14.01 |
| Peripheral blood (FACS) | Mature Bcell | 2.57 | 0.52 | 46.85 |
| Peripheral blood (FACS) | Monocyte | 0.94 | 13.00 | 20.86 |
| Peripheral blood (FACS) | Nkcell | 3.41 | 2.46 | 30.31 |
| Peripheral blood (FACS) | NKTcell | 13.95 | 1.78 | 44.62 |
| Peripheral blood (FACS) | Peripheral blood lymphocyte FACS T regulatory | 0.16 | 1.83 | 46.87 |
| Peripheral blood (FACS) | Peripheral blood lymphocyte FACS Total T cell | 19.02 | 5.10 | 18.21 |
| Peripheral blood (FACS) | Peripheral blood lymphocyte FACS T helper | 15.26 | 3.15 | 23.95 |
| Peripheral blood (FACS) | Peripheral blood lymphocyte FACS T cytotoxic | 10.90 | 5.61 | 27.06 |
| Plasma chemistry | Albumin | 4.39 | 18.04 | 25.47 |
| Plasma chemistry | Alkaline Phosphatase | 10.31 | 7.97 | 15.74 |
| Plasma chemistry | Alanine Aminotransferase | 5.38 | 23.80 | 13.07 |
| Plasma chemistry | Aspartate Aminotransferase | 1.40 | 17.85 | 16.32 |
| Plasma chemistry | Calcium | 0.24 | 20.25 | 32.26 |
| Plasma chemistry | Cholesterol | 41.52 | 14.31 | 7.96 |
| Plasma chemistry | Creatine Kinase | 1.81 | 0.76 | 23.25 |
| Plasma chemistry | chloride | 2.54 | 3.84 | 49.48 |
| Plasma chemistry | Iron | 8.10 | 5.16 | 8.86 |
| Plasma chemistry | Fructosamine | 7.13 | 0.57 | 49.87 |
| Plasma chemistry | Glucose | 9.53 | 0.37 | 21.31 |
| Plasma chemistry | Glycerol | 8.65 | 0.37 | 24.60 |
| Plasma chemistry | High Density Lipoprotein | 40.79 | 13.07 | 10.00 |
| Plasma chemistry | Potassium | 17.75 | 1.62 | 27.68 |
| Plasma chemistry | Low Density Lipoprotein | 31.90 | 13.74 | 13.50 |
| Plasma chemistry | Magnesium | 8.38 | 1.42 | 26.07 |
| Plasma chemistry | Sodium | 0.26 | 0.22 | 55.02 |
| Plasma chemistry | Non-esterified free fatty acids | 3.44 | 0.01 | 27.22 |
| Plasma chemistry | Inorganic Phosphorus | 2.66 | 0.08 | 29.13 |
| Plasma chemistry | Total Bilirubin | 0.27 | 0.04 | 19.82 |
| Plasma chemistry | Triglycerides | 2.40 | 3.27 | 26.82 |
| Plasma chemistry | Uric Acid | 12.02 | 0.02 | 14.44 |
| Plasma chemistry | Amylase | 43.23 | 3.51 | 15.27 |
| Plasma chemistry | Creatinine | 0.47 | 0.00 | 45.76 |
| Plasma chemistry | Lactate dehydrogenase | 11.55 | 17.24 | 13.44 |
| Plasma chemistry | Thyroxine | 38.52 | 0.00 | 21.49 |
| Plasma chemistry | Total protein | 6.84 | 22.11 | 26.65 |
| Plasma chemistry | Urea | 0.05 | 0.90 | 20.54 |
| Weight curve | Weight wk 14 | 46.68 |  | 12.10 |
| Weight curve | Weight wk 16 | 45.87 |  | 9.85 |
| Weight curve | Weight wk 9 | 51.97 |  | 11.26 |
| Weight curve | Weight wk 10 | 49.00 |  | 11.13 |
| Weight curve | Weight wk 16 | 42.82 |  | 11.02 |
