# Supplementary material for: Robust and Sensitive Analysis of Mouse Knockout Phenotypes
Source: PLoS One. 2012 Dec 26;7(12):e52410. doi: 10.1371/journal.pone.0052410 (PMC3530558; doi:10.1371/journal.pone.0052410)
Supplement: Table S2 — Temporal effects on batch means in controls. Legend: A runs test statistics of the mean of each control batch in a time series from the B6Brd;B6N-Tyrc-Brd for each sex from the Mouse GP pipeline. There was no evidence for autocorrelation after adjusting for multiple testing (method: Bonferroni). (DOCX) [file pone.0052410.s002.docx]

Supplementary Table 3: Temporal effects on batch means in controls.

| Variable | Males | | Females | |
| --- | --- | --- | --- | --- |
|  | Standardised run statistic | *p*-value | Standardised run statistic | *p*-value |
| Fat mass | 0.7475 | 0.4547 | 0.6433 | 0.52 |
| Lean mass | -0.1054 | 0.916 | -0.1054 | 0.916 |
| Weight | -0.9373 | 0.3486 | 1.014 | 0.3106 |
| Length | 2.4625 | 0.0138 | 0.8394 | 0.4012 |
| Bone mineral density | 0.4289 | 0.668 | -1.2866 | 0.1982 |
| Bine mineral content | 0.4289 | 0.668 | -0.8577 | 0.391 |
| Fat percentage | 0.1078 | 0.9141 | 0.2144 | 0.8302 |
