# Supplementary material for: Robust and Sensitive Analysis of Mouse Knockout Phenotypes
Source: PLoS One. 2012 Dec 26;7(12):e52410. doi: 10.1371/journal.pone.0052410 (PMC3530558; doi:10.1371/journal.pone.0052410)
Supplement: Table S3 — A comparison of phenotypic calls. Legend: A comparison between genotype calls of significance when the mixed model excludes (Eq.1) or includes weight (Eq.2). Presented are body weight and three clinical chemistry variables for Slc25a21tm1a(KOMP)Wtsi on the B6Brd;B6N-Tyrc-Brd genetic background. Where a call of significance is made, (↑) indicates that the genotype effect gave an increase in the variable, whilst (↓) indicates a decrease. NA is used to indicate that weight as the variable of interest cannot be fitted with Eq.2 as this includes weight as a covariate. A dash indicates that genotype was not significant. Significant MM calls were controlled to have a false discovery rate of 0.05. (DOCX) [file pone.0052410.s003.docx]

Supplementary Table 8: A comparison of phenotypic calls.

| Phenotypes | MM | |
| --- | --- | --- |
|  | Eq.1 | Eq.2 |
| Body weight | ⇩ | **NA** |
| Alanine aminotransferase | ⇩ | - |
| Albumin | ⇩ | - |
| Aspartate aminotransferanse | ⇩ | - |
