# Supplementary material for: Robust and Sensitive Analysis of Mouse Knockout Phenotypes
Source: PLoS One. 2012 Dec 26;7(12):e52410. doi: 10.1371/journal.pone.0052410 (PMC3530558; doi:10.1371/journal.pone.0052410)
Supplement: File S2 — Detailed mixed model output for the allele Ppp3catm2e(EUCOMM)Wtsi and associated DEXA data. Legend: For each trait studied, for each model fitting procedures, the final model output was captured and the data visualised with a boxplot. Furthermore, to test the quality of model fit, a number of graphical diagnostic plots were generated for each gene and trait. (PDF) [file pone.0052410.s005.pdf]

Ppp3ca$^{tm2e(EUCOMM)Wtsi}$
DEXA mixed model analysis

Variable	Unit of analysis
Weight	g
Nose to tail length	cm
Bone mineral density	g/cm^2
Bone mineral content	g
Lean mass	g
Fat mass	g
Fat percentage	%

Abbreviations:
LRT: Likelihood ratio test
ML: Maximum likelihood
REML: Residual maximum likelihood
BMC: Bone mineral content
BMD: Bone mineral density
LM: Lean mass
FM: Fat mass
Fat %: Fat percentage
SE: Standard Error

Information on diagnostic graphs presented for each model

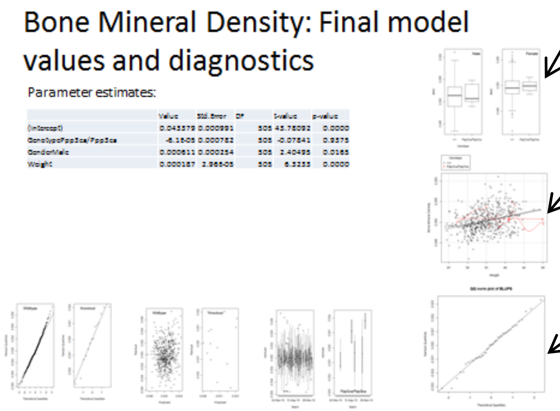

A: A boxplot comparison of the dependent variable for each genotype for each sex.

B: Weight versus dependent variable scatterplot For each genotype a regression and a Loess line is fitted. A Loess line is a locally weighted linear line.

C: Normal Q-Q plot of the best linear unbiased prediction of random effects (BLUPS).

D: For each genotype group, conditional raw residues are plotted versus batch.

E: For each genotype group, a plot of conditional raw residue versus predicted values.

F: Normal Q-Q plots of conditional raw residues for each genotype.

Mixed Model results 1

Starting model:

$$Y_{ij} = \beta_o + \beta_1 Genotype1_{ij} + \beta_2 Sex1_{ij} + \beta_3 Genotype1_{ij} Sex1_{ij} + u_j + e_{ij}.$$

Weight: Top down modelling output

Hypothesis	Model1	Model 2	Test	Estimation method	Test statistic value	*p*-value
Is batch significant?	Batch	No batch	LRT	REML	$\chi^2(0:1)=45.34242$	<.0001
Is variance homogenous?	Homogenous	Heterogeneous	LRT	REML	$\chi^2(2)=9.619678$	0.0019
Testing fixed effects – sex			Type 1 *F*-test	REML	$F(1,521)=489.52$	<.0001
Testing fixed effects – sex*genotype			Type 1 *F*-test	REML	$F(1,521)=0.106$	0.7444
Testing treatment - Is genotype significant?	With genotype	Without genotype	LRT	ML	$\chi^2(2)=9.1817$	0.0024

Weight: Final model values and diagnostics

Parameter estimates:

	Value	Std.Error	DF	t-value	p-value
(Intercept)	32.16134	0.28105	522	114.4329	0.0000
GenotypePpp3ca/Ppp3ca	5.10983	1.504202	522	3.39703	0.0007
sexMale	5.8688	0.264353	522	22.20065	0.0000

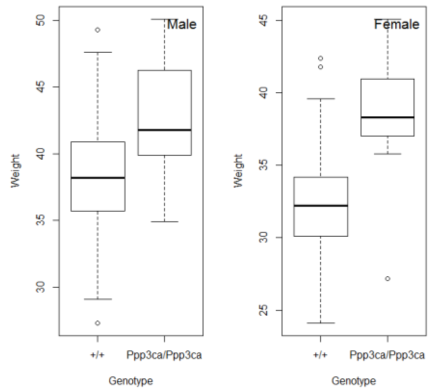

Graph B: N/A for weight as dependent variable

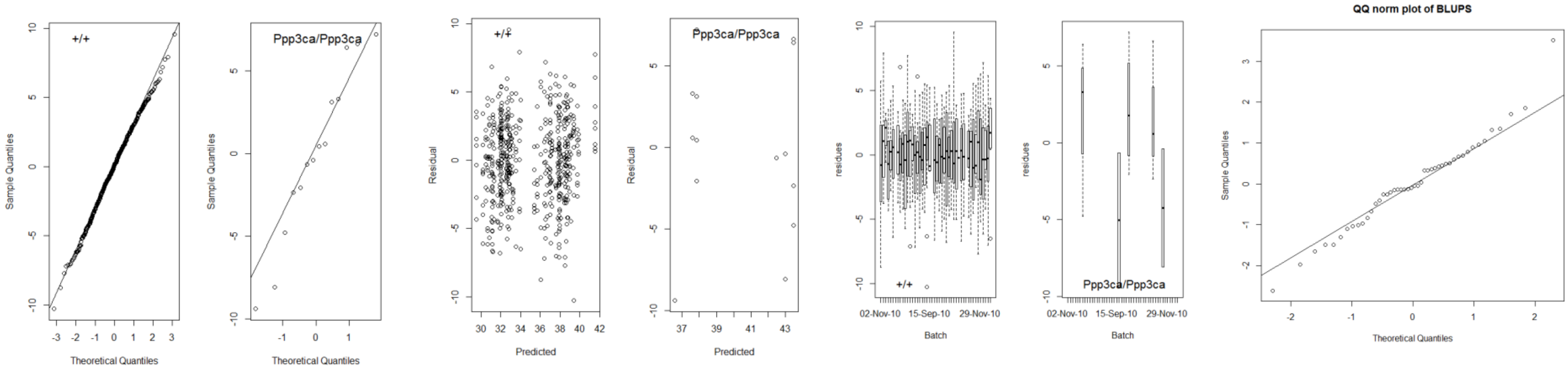

Nose to tail length:
Top down modelling output

Hypothesis	Model1	Model 2	Test	Estimation method	Test statistic value	*p*-value
Is batch significant?	Batch	No batch	LRT	REML	$\chi^2(0{:}1)=364.037$	<0.0001
Is variance homogenous?	Homogenous	Heterogeneous	LRT	REML	$\chi^2(2)=0.94$	0.3322
Testing fixed effects – sex			Type 1 *F*-test	REML	$F(1,521)=17.76$	0.000
Testing fixed effects – sex*genotype			Type 1 *F*-test	REML	$F(1,521)=0.57$	0.5659
Testing treatment - Is genotype significant?	With genotype	Without genotype	LRT	ML	$\chi^2(2)= 1.56$	0.211

Nose to tail length: Final model values and diagnostics

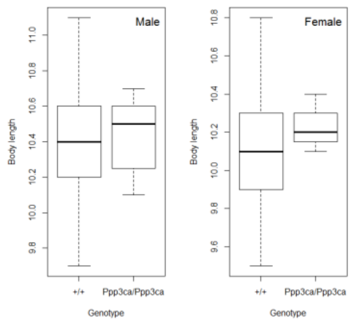

Parameter estimates:

	Value	Std.Error	DF	t-value	p-value
(Intercept)	10.1177	0.0323	522	313.34	0.0000
GenotypePpp3ca/Ppp3ca	0.0877	0.0705	522	1.24	0.2143
GenderMale	0.2708	0.0151	522	17.93	0.0000

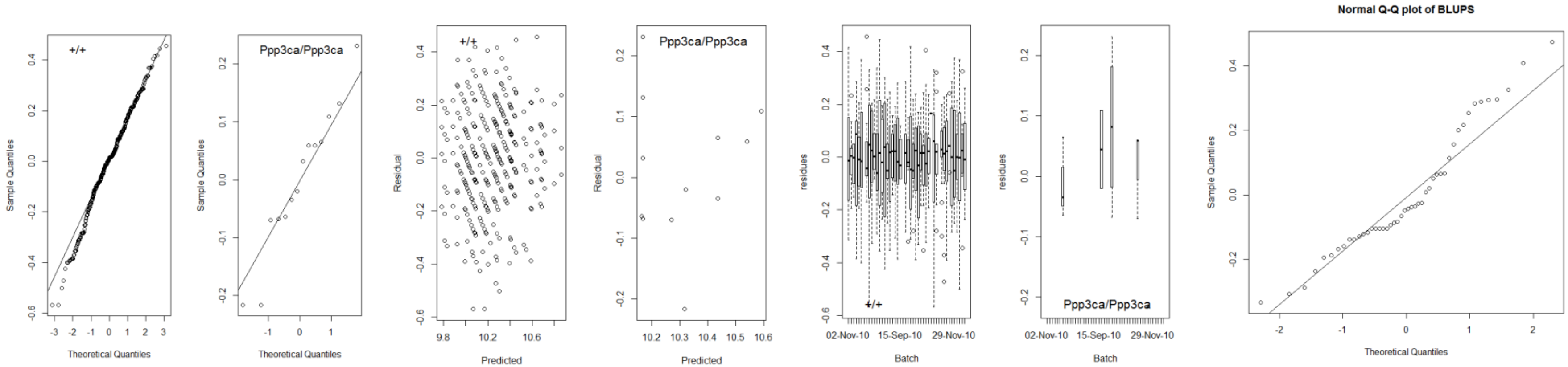

Bone mineral Density: Top down modelling output

Hypothesis	Model1	Model 2	Test	Estimation method	Test statistic value	*p*-value
Is batch significant?	Batch	No batch	LRT	REML	$\chi^2(0:1)= 98.11$	<0.000 1
Is variance homogenous?	Homogenous	Heterogeneous	LRT	REML	$\chi^2(2)=2.059$	0.1513
Testing fixed effects – sex			Type 1 F-test	REML	F(1,507)=8.67	0.000
Testing fixed effect – genotype*sex			Type 1 F-test	REML	F(1,507)=-0.25	0.7973
Testing treatment - Is genotype significant?	With genotype	Without genotype	LRT	ML	$\chi^2(2)=1.172$	0.279

Bone Mineral Density: Final model values and diagnostics

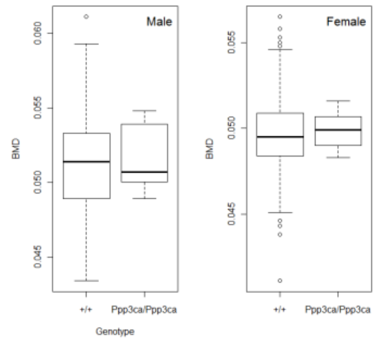

Parameter estimates:

	Value	Std.Error	DF	t-value	p-value
(Intercept)	0.0494	0.0003	508	192.23	0.0000
GenotypePpp3ca/Ppp3ca	0.0008	0.0008	508	1.08	0.2794
GenderMale	0.0017	0.0002	508	8.76	0.0000

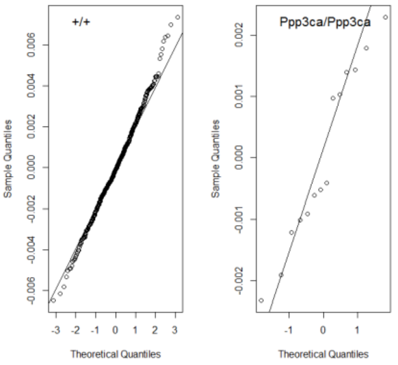

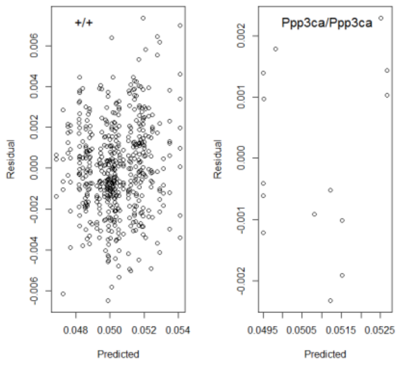

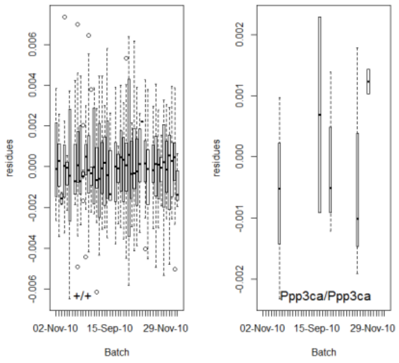

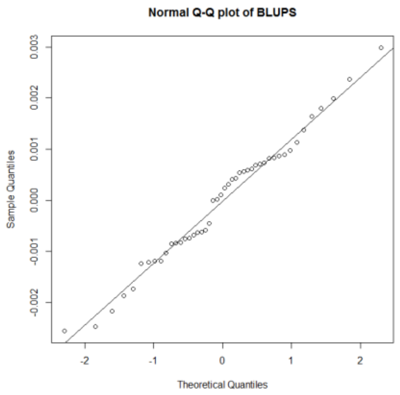

Bone Mineral Content: Top down modelling output

Hypothesis	Model1	Model 2	Test	Estimation method	Test statistic value	*p*-value
Is batch significant?	Batch	No batch	LRT	REML	$\chi^2(0:1)=20.26$	<0.0001
Is variance homogenous?	Homogenous	Heterogeneo us	LRT	REML	$\chi^2(2)=0.112$	0.7376
Testing fixed effects – sex			Type 1 *F*-test	REML	F(1,507)=16.40	0.0000
Testing fixed effect – genotype*sex			Type 1 *F*-test	REML	F(1,507)=-2.0296	0.0429
Testing treatment - Is genotype significant?	With genotype	Without genotype	LRT	ML	$\chi^2(2)=11.70$	0.00287

Bone Mineral Content: Final model values and diagnostics

Parameter estimates:

	Value	Std.Error	DF	t-value	p-value
(Intercept)	0.4527	0.0032	507	143.26	0.0000
GenotypePpp3ca/Ppp3ca	0.0548	0.0161	507	3.41	0.0007
GenderMale	0.0561	0.0034	507	16.41	0.0000
GenotypePpp3ca/Ppp3ca:GenderMale	-0.0450	0.0222	507	-2.03	0.0429

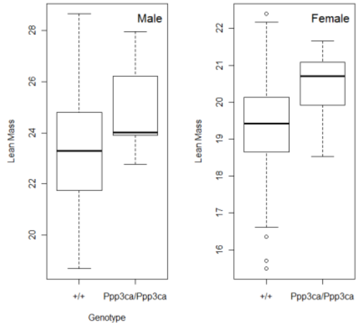

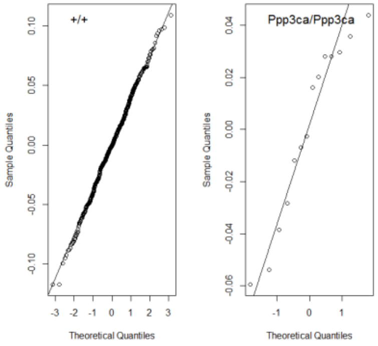

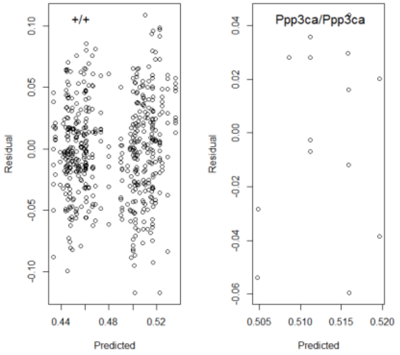

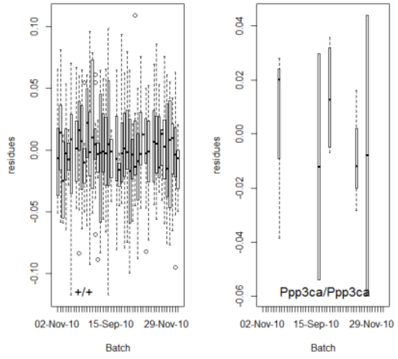

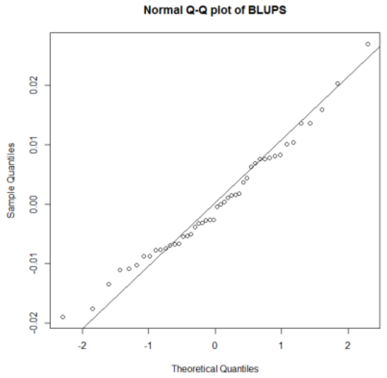

Lean Mass:
Top down modelling output

Hypothesis	Model1	Model 2	Test	Estimation method	Test statistic value	*p*-value
Is batch significant?	Batch	No batch	LRT	REML	$\chi^2(0:1)$=23.29	<0.0001
Is variance homogenous?	Homogenous	Heterogeneous	LRT	REML	$\chi^2(2)$=0.01259	0.8996
Testing fixed effects – sex			Type 1 *F*-test	REML	F(1,507)= 29.088	0.0543
Testing fixed effect – genotype*sex			Type 1 *F*-test	REML	F(1,507)=0.607	0.5438
Testing treatment - Is genotype significant?	With genotype	Without genotype	LRT	ML	$\chi^2(2)$=9.926	0.00162

Lean Mass: Final model values and diagnostics

Parameter estimates:

	Value	Std.Error	DF	t-value	p-value
(Intercept)	19.3865	0.1231	508	157.52	0.0000
GenotypePpp3ca/Ppp3ca	1.4604	0.4629	508	3.15	0.0017
GenderMale	3.8690	0.1307	508	29.60	0.0000

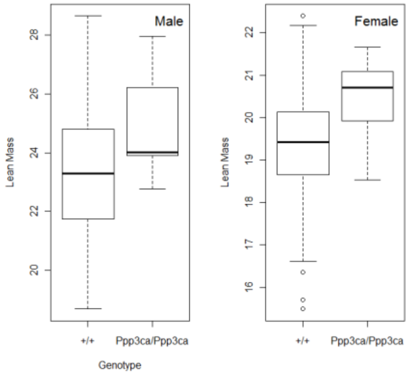

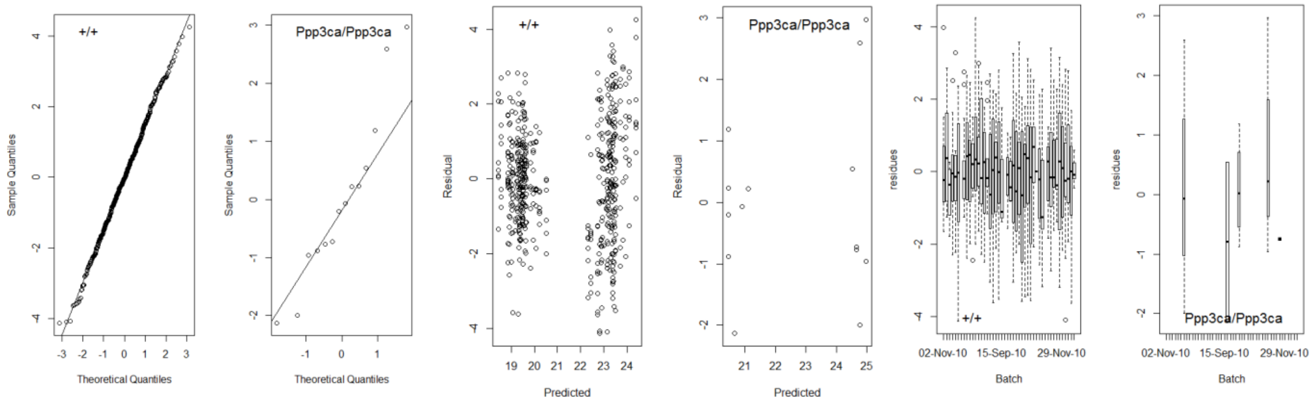

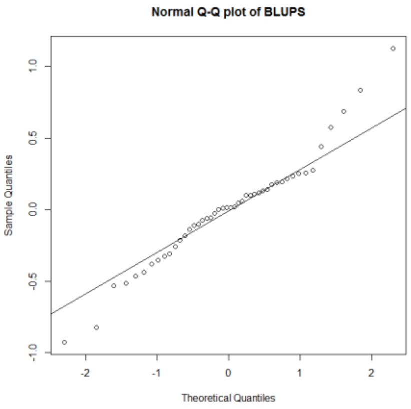

Fat Mass: Final model values and diagnostics

Hypothesis	Model1	Model 2	Test	Estimation method	Test statistic value	*p*-value
Is batch significant?	Batch	No batch	LRT	REML	$\chi^2(0:1)=47.56$	<.0001
Is variance homogenous?	Homogenous	Heterogeneous	LRT	REML	$\chi^2(2)=4.588$	0.0322
Testing fixed effects – sex			Type 1 F-test	REML	$F(1,507)= 29.08$	0.0000
Testing fixed effect – genotype*sex			Type 1 F-test	REML	$F(1,507)=0.607$	0.5438
Testing treatment - Is genotype significant?	With genotype	Without genotype	LRT	ML	$\chi^2(2)=9.14$	0.00248

Fat Mass: Final model values and diagnostics

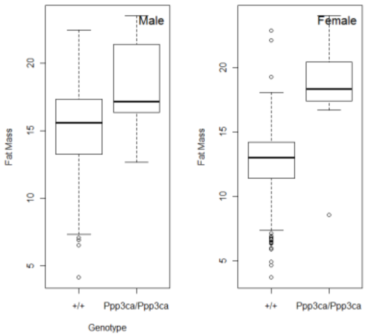

Parameter estimates:

	Value	Std.Error	DF	t-value	p-value
(Intercept)	12.84	0.26	508	49.65	0.0000
GenotypePpp3ca/Ppp3ca	4.03	1.19	508	3.38	0.0008
GenderMale	2.12	0.24	508	8.91	0.0000

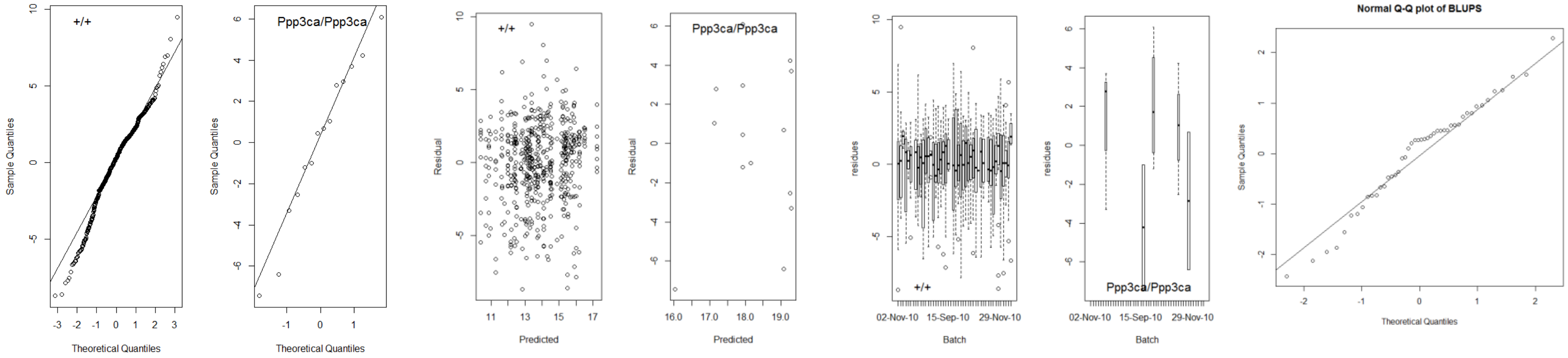

Dependent variable: Fat Percentage

Hypothesis	Model1	Model 2	Test	Estimation method	Test statistic value	*p*-value
Is batch significant?	Batch	No batch	LRT	REML	$\chi^2(0{:}1)=49.96$	<.0001
Is variance homogenous?	Homogenous variance	Heterogeneous	LRT	REML	$\chi^2(2)=0.123$	0.7256
Testing fixed effects – sex			Type 1 F-test	REML	F(1,507)=-1.18	0.2378
Testing fixed effect – genotype*sex			Type 1 F-test	REML	F(1,507)=-0.88	0.4023
Is genotype significant?	With genotype	Without genotype	LRT	ML	$\chi^2(2)=6.45$	0.0110

Fat %: Final model values and diagnostics

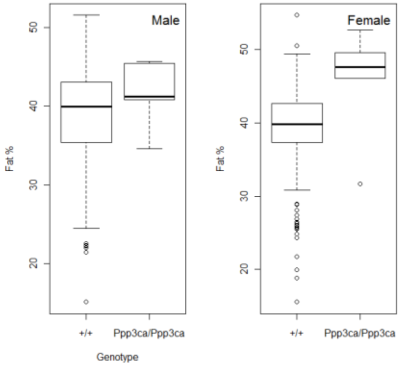

Parameter estimates:

	Value	Std.Error	DF	t-value	p-value
(Intercept)	39.0739	0.4441	509	87.99	0.0000
GenotypePpp3ca/Ppp3ca	4.2765	1.6820	509	2.54	0.0113

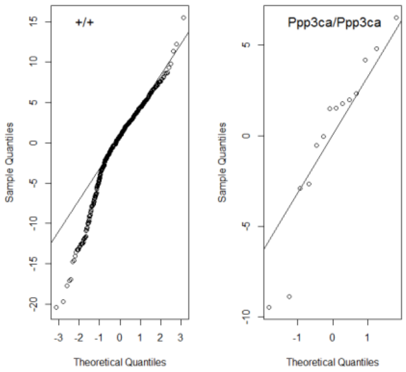

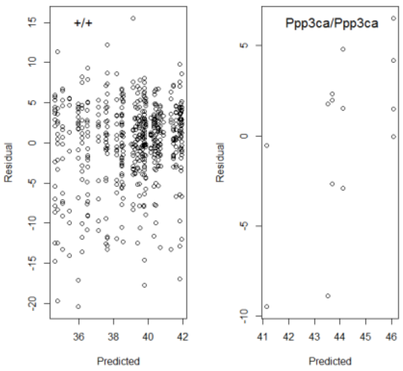

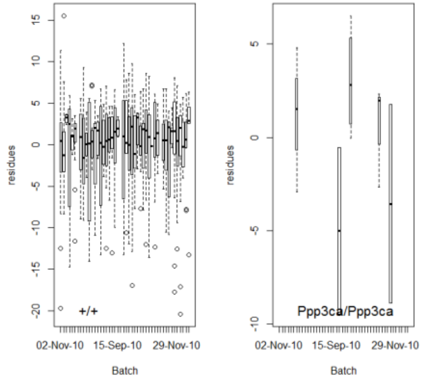

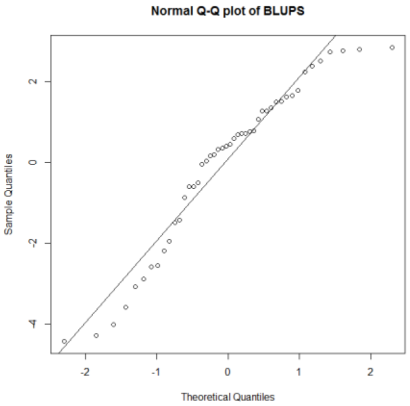

Summary

Variable	*p* value	Adjusted *p*' value	Genotype Estimate ± SE	Sex
Weight	0.0024	0.0051	Y↑ 5.109±1.504	Y↑
Nose to tail length	0.211	0.2569	N	Y↑
BMD	0.279	0.3224	N	Y↑
BMC	0.00287	0.0057	Y↑ 0.05±0.02	Y↑
LM	0.00162	0.0038	Y↑ 1.46±0.46	Y↑
FM	0.00248	0.0051	Y↑ 4.03±1.19	Y↓
Fat %	0.0110	0.0184	Y↑ 4.27±1.68	N

Y denotes a statistically significant effect and N indicates a non significant effect. The ↑ symbol indicates a positive estimated regression coefficient such that this effect leads to an increase in the dependent variable. Whilst, the ↓ symbol indicates a negative estimated regression coefficient such that this effect leads to a decrease in the dependent variable.

Mixed Model results 2

Starting model:

$$Y_{ij} = \beta_o + \beta_1 Genotype1_{ij} + \beta_2 Sex1_{ij} + \beta_3 Weight1_{ij\,+}\,\beta_4 Genotype1_{ij} Sex1_{ij} + u_j + e_{ij}.$$

Nose to tail length: Top down modelling output

Hypothesis	Model1	Model 2	Test	Estimation method	Test statistic value	*p*-value
Is batch significant?	Batch	No batch	LRT	REML	$\chi^2(0{:}1)=460.5077$	<.0001
Is variance homogenous?	Homogenous	Heterogeneous	LRT	REML	$\chi^2(2)=0.4140409$	0.5199
Testing fixed effects – sex			Type 1 *F*-test	REML	F(1,518)=33.87	<.0001
Testing fixed effects – sex*genotype			Type 1 *F*-test	REML	F(1,518)=0.16	0.6925
Testing fixed effects – Weight			Type 1 *F*-test	REML	F(1,518)=210.25	<.0001
Testing treatment - Is genotype significant?	With genotype	Without genotype	LRT	ML	$\chi^2(2)= 1.098737$	0.2945

Nose to tail length: Final model values and diagnostics

Parameter estimates:

	Value	Std.Error	DF	t-value	p-value
(Intercept)	9.16105	0.073262	519	125.0459	0.0000
GenotypePpp3ca/Ppp3ca	-0.0651	0.062133	519	-1.04768	0.2953
sexMale	0.101091	0.017354	519	5.82526	0.0000
Weight	0.029709	0.002046	519	14.52403	0.0000

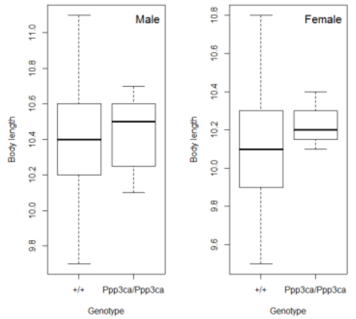

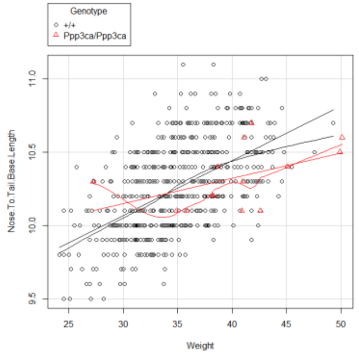

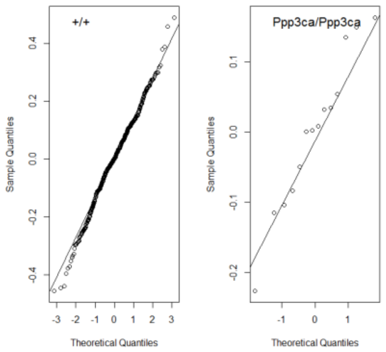

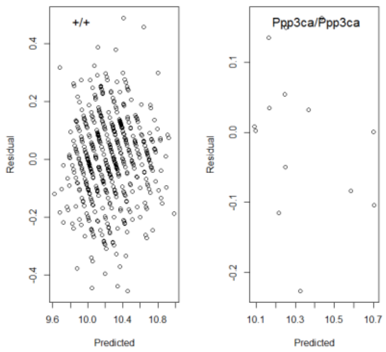

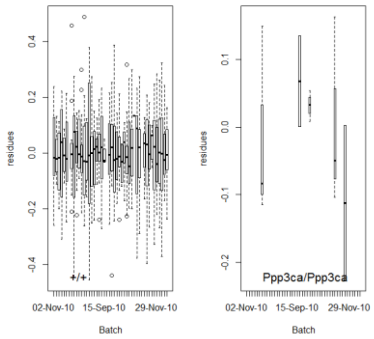

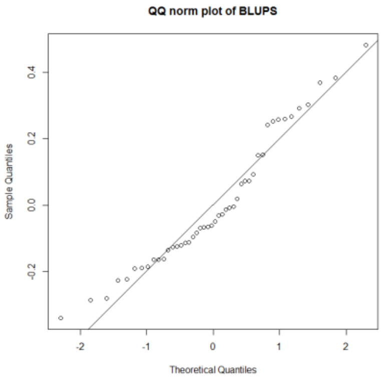

Bone mineral Density:
Top down modelling output

Hypothesis	Model1	Model 2	Test	Estimation method	Test statistic value	*p*-value
Is batch significant?	Batch	No batch	LRT	REML	$\chi^2(0:1)= 121.8778$	<.0001
Is variance homogenous?	Homogenous	Heterogeneous	LRT	REML	$\chi^2(2)=0.6221509$	0.4302
Testing fixed effects – sex			Type 1 F-test	REML	$F(1,504)=5.76$	0.0168
Testing fixed effects – weight			Type 1 F-test	REML	$F(1,504)=39.85$	<.0001
Testing fixed effect – genotype*sex			Type 1 F-test	REML	$F(1,504)=0.02$	0.8860
Testing treatment - Is genotype significant?	With genotype	Without genotype	LRT	ML	$\chi^2(2)=0.006628$	0.9351

Bone Mineral Density: Final model values and diagnostics

Parameter estimates:

	Value	Std.Error	DF	t-value	p-value
(Intercept)	0.043379	0.000991	505	43.78092	0.0000
GenotypePpp3ca/Ppp3ca	-6.1E-05	0.000782	505	-0.07841	0.9375
sexMale	0.000611	0.000254	505	2.40495	0.0165
Weight	0.000187	2.96E-05	505	6.3233	0.0000

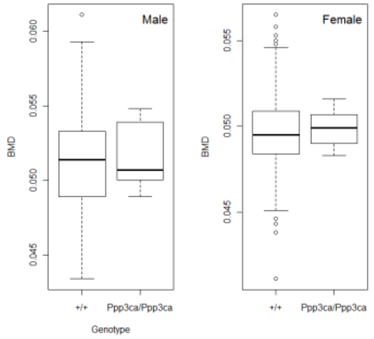

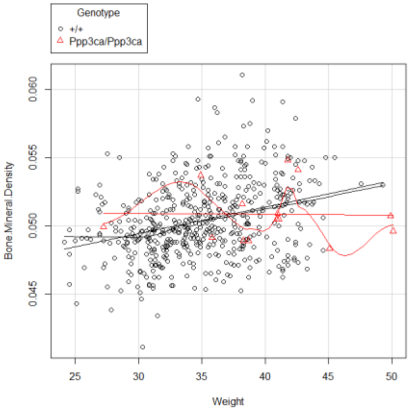

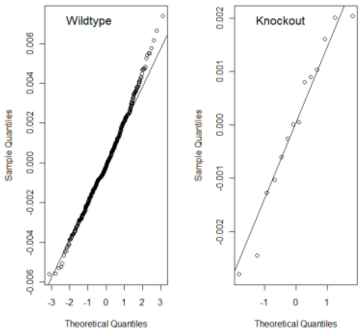

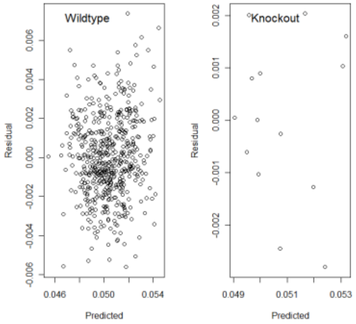

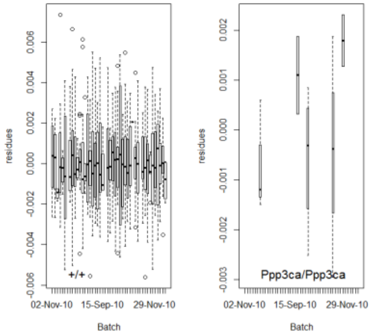

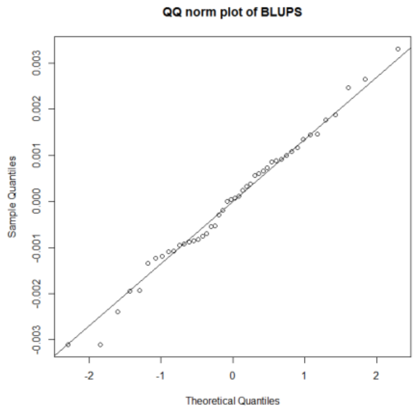

Bone Mineral Content:
Top down modelling output

Hypothesis	Model1	Model 2	Test	Estimation method	Test statistic value	*p*-value
Is batch significant?	Batch	No batch	LRT	REML	$\chi^2(0:1)=37.100$	<.0001
Is variance homogenous?	Homogenous	Heterogeneous	LRT	REML	$\chi^2(2)=0.715$	0.3975
Testing fixed effects – sex			Type 1 *F*-test	REML	$F(1,504)=20.34$	<.0001
Testing fixed effects – weight			Type 1 *F*-test	REML	$F(1,504)=219.99$	<.0001
Testing fixed effect – genotype*sex			Type 1 *F*-test	REML	$F(1,504)=4.25$	0.0397
Testing treatment - Is genotype significant?	With genotype	Without genotype	LRT	ML	$\chi^2(2)=4.2895$	0.117

Bone Mineral Content: Final model values and diagnostics

Parameter estimates:

	Value	Std.Error	DF	t-value	p-value
(Intercept)	0.24082	0.014562	504	16.53746	0.0000
GenotypePpp3ca/Ppp3ca	0.019172	0.01394	504	1.375339	0.1696
sexMale	0.017501	0.00388	504	4.510771	0.0000
Weight	0.006577	0.000443	504	14.83205	0.0000
GenotypePpp3ca/Ppp3ca:sexMale	-0.03862	0.018728	504	-2.06234	0.0397

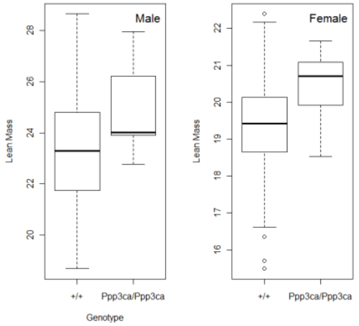

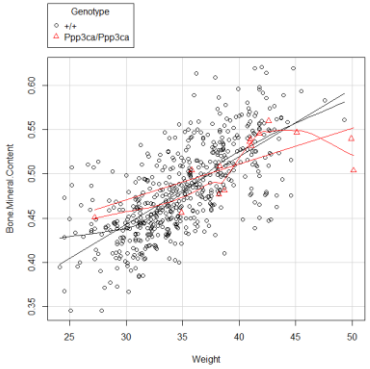

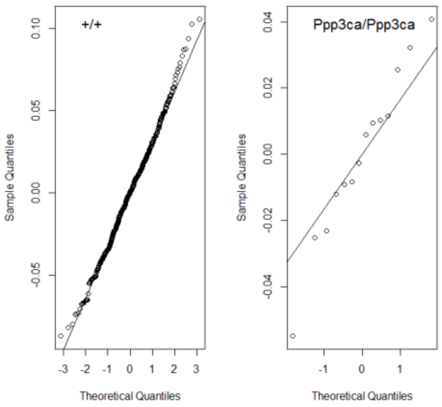

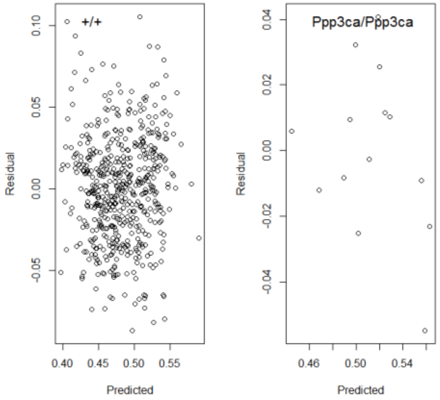

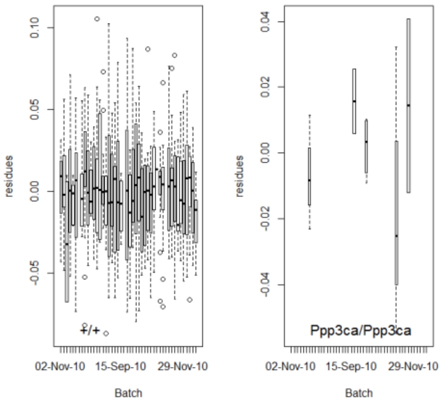

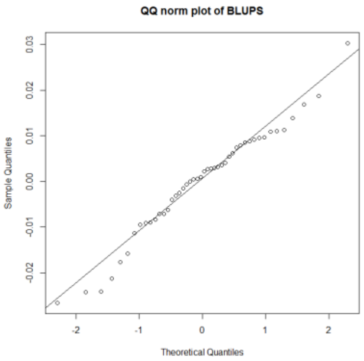

Lean Mass:
Top down modelling output

Hypothesis	Model1	Model 2	Test	Estimation method	Test statistic value	*p*-value
Is batch significant?	Batch	No batch	LRT	REML	$\chi^2(0:1)=38.03007$	<0.0001
Is variance homogenous?	Homogenous	Heterogeneous	LRT	REML	$\chi^2(2)=4.872048$	0.0273
Testing fixed effects – sex			Type 1 *F*-test	REML	F(1,504)= 248.64	<0.0001
Testing fixed effects – weight			Type 1 *F*-test	REML	F(1,504)=253.87	<0.0001
Testing fixed effect – genotype*sex			Type 1 *F*-test	REML	F(1,504)=2.50	0.1147
Testing treatment - Is genotype significant?	With genotype	Without genotype	LRT	ML	$\chi^2(2)=0.1692225$	0.6808

Lean Mass: Final model values and diagnostics

Parameter estimates:

	Value	Std.Error	DF	t-value	p-value
(Intercept)	11.02164	0.540301	505	20.39907	0.0000
GenotypePpp3ca/Ppp3ca	0.121513	0.300137	505	0.404859	0.6858
sexMale	2.368817	0.14466	505	16.37505	0.0000
Weight	0.259955	0.016452	505	15.80078	0.0000

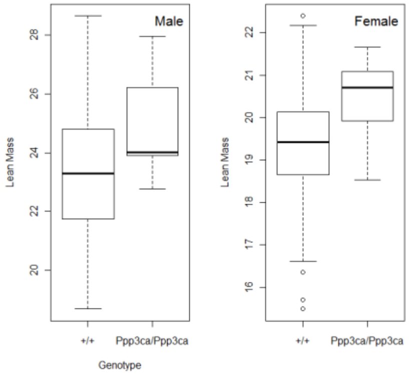

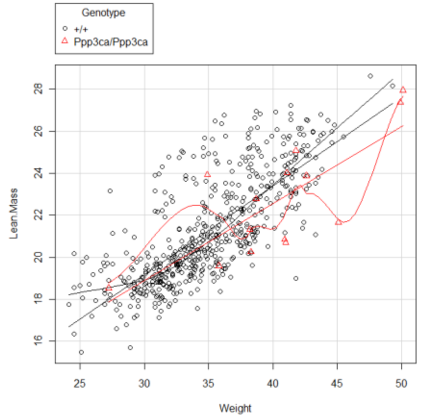

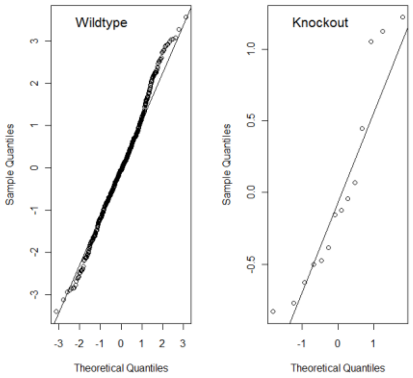

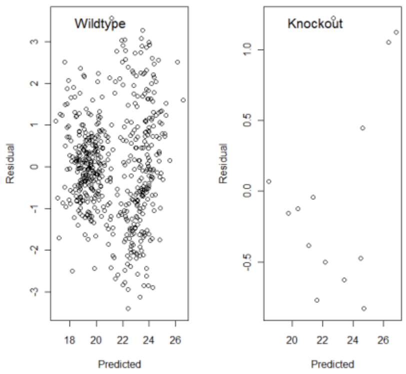

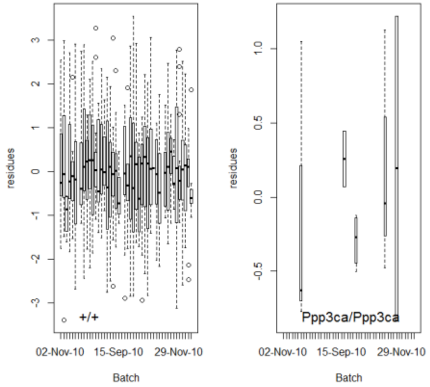

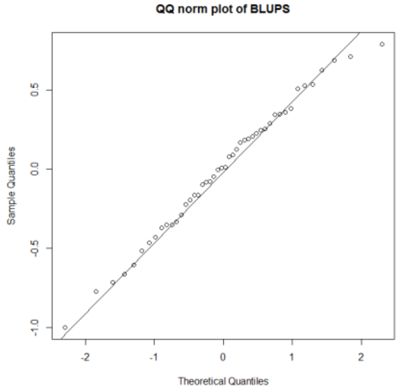

Fat Mass: Final model values and diagnostics

Hypothesis	Model1	Model 2	Test	Estimation method	Test statistic value	*p*-value
Is batch significant?	Batch	No batch	LRT	REML	$\chi^2(0:1)=29.37313$	<.0001
Is variance homogenous?	Homogenous	Heterogeneous	LRT	REML	$\chi^2(2)=10.05999$	0.0015
Testing fixed effects – sex			Type 1 F-test	REML	F(1,504)= 234.69	<.0001
Testing fixed effects – weight			Type 1 F-test	REML	F(1,504)=2133.41	<.0001
Testing fixed effect – genotype*sex			Type 1 F-test	REML	F(1,504)=3.881	0.0494
Testing treatment - Is genotype significant?	With genotype	Without genotype	LRT	ML	$\chi^2(2)=4.404697$	0.1105

Fat Mass: Final model values and diagnostics

Parameter estimates:

	Value	Std.Error	DF	t-value	p-value
(Intercept)	-11.8357	0.545822	504	-21.6841	0.0000
GenotypePpp3ca/Ppp3ca	0.542815	0.313922	504	1.72914	0.0844
sexMale	-2.38563	0.155723	504	-15.3197	0.0000
Weight	0.767172	0.01661	504	46.18881	0.0000
GenotypePpp3ca/Ppp3ca:sexMale	-0.72318	0.367111	504	-1.96992	0.0494

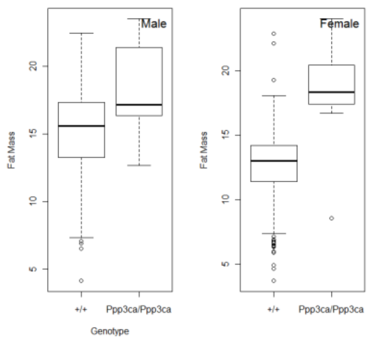

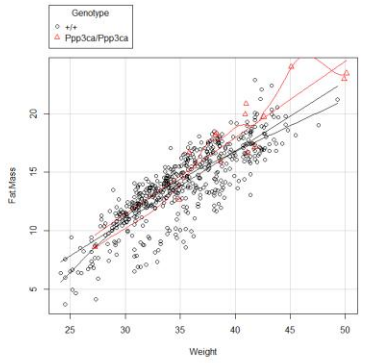

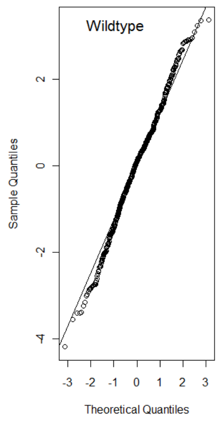

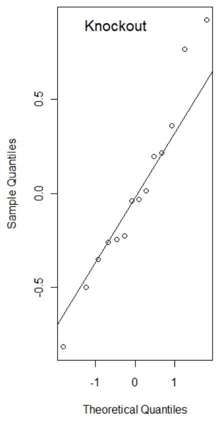

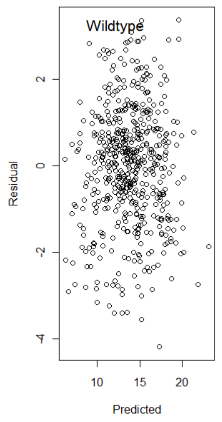

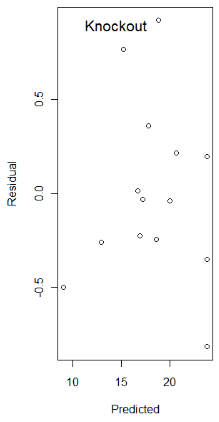

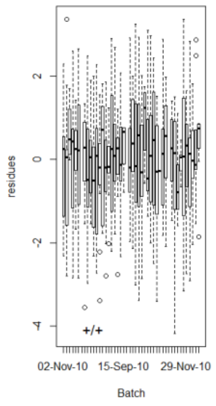

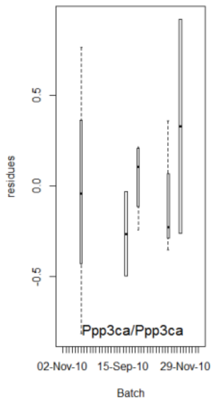

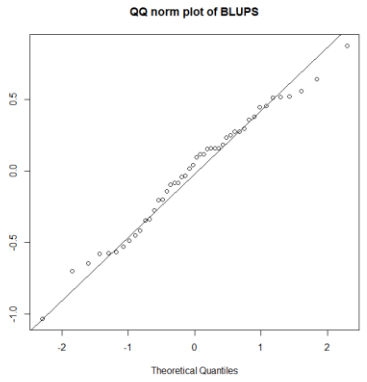

Dependent variable: Fat Percentage

Hypothesis	Model1	Model 2	Test	Estimation method	Test statistic value	*p*-value
Is batch significant?	Batch	No batch	LRT	REML	$\chi^2(0:1)=29.69792$	<.0001
Is variance homogenous?	Homogenous variance	Heterogeneous	LRT	REML	$\chi^2(2)=3.791177$	0.0515
Testing fixed effects – sex			Type 1 F-test	REML	$F(1,504)=229.20$	<.0001
Testing fixed effects – weight			Type 1 F-test	REML	$F(1,504)=434.17$	<.0001
Testing fixed effect – genotype*sex			Type 1 F-test	REML	$F(1,504)=0.491$	0.4837
Is genotype significant?	With genotype	Without genotype	LRT	ML	$\chi^2(2)=1.048$	0.3059

Fat %: Final model values and diagnostics

Parameter estimates:

	Value	Std.Error	DF	t-value	p-value
(Intercept)	3.862608	1.733795	505	2.227835	0.0263
GenotypePpp3ca/Ppp3ca	-1.27778	1.253292	505	-1.01954	0.3084
sexMale	-7.09533	0.461284	505	-15.3817	0.0000
Weight	1.104836	0.052953	505	20.86467	0.0000

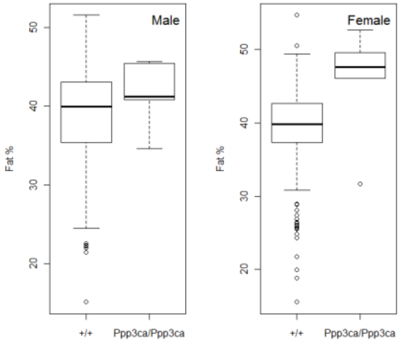

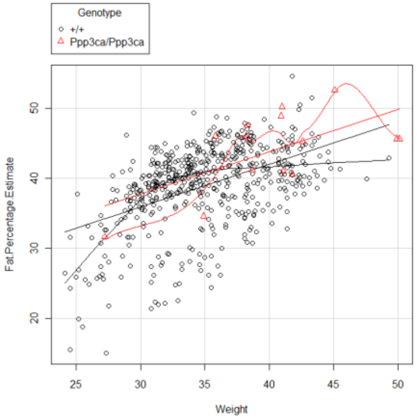

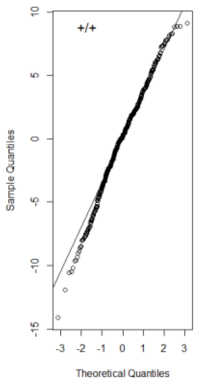

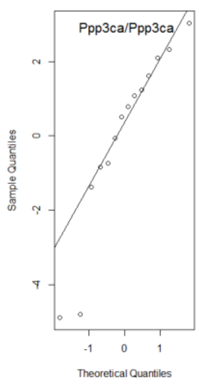

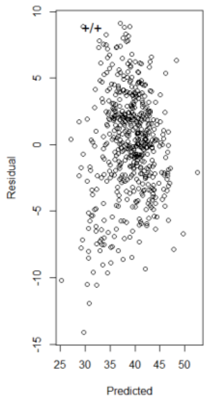

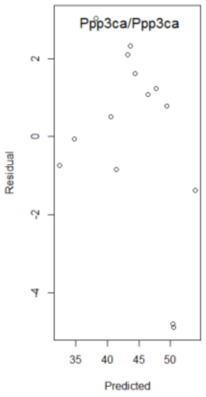

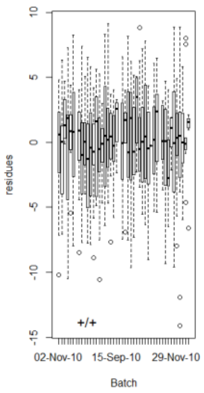

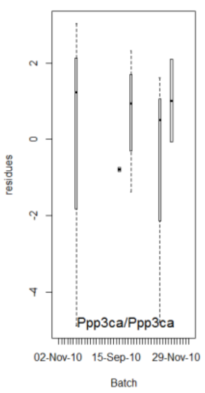

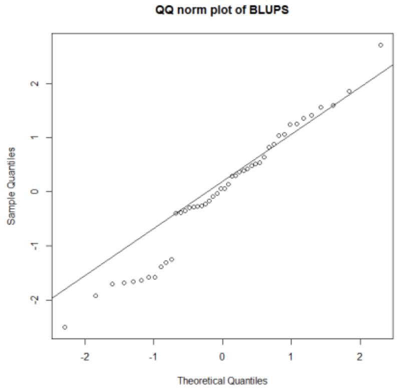

Summary

Variable	*p* value	Adjusted *p*' value	Genotype Estimate ± SE	Sex	Weight
Nose to tail length	0.2945	0.3329	N	Y↑	Y↑
BMD	0.9351	0.9351	N	Y↑	Y↑
BMC	0.117	0.1560	N	Y↑	Y↑
LM	0.6808	0.6983	N	Y↑	Y↑
FM	0.1105	0.1512	N	Y↓	Y↑
Fat %	0.3059	0.3384	N	Y↓	Y↑

Y denotes a statistically significant effect and N indicates a non significant effect. The ↑ symbol indicates a positive estimated regression coefficient such that this effect leads to an increase in the dependent variable. Whilst, the ↓ symbol indicates a negative estimated regression coefficient such that this effect leads to a decrease in the dependent variable.
