# Supplementary material for: Robust and Sensitive Analysis of Mouse Knockout Phenotypes
Source: PLoS One. 2012 Dec 26;7(12):e52410. doi: 10.1371/journal.pone.0052410 (PMC3530558; doi:10.1371/journal.pone.0052410)
Supplement: File S3 — Detailed mixed model output for the allele Slc25a21tm1a(KOMP)Wtsi and associated DEXA data. Legend: For each trait studied, for each model fitting procedures, the final model output was captured and the data visualised with a boxplot. Furthermore, to test the quality of model fit, a number of graphical diagnostic plots were generated for each gene and trait. (PDF) [file pone.0052410.s006.pdf]

# *Slc25a21*$^{tm1a(KOMP)Wtsi}$

## DEXA mixed model analysis

| Variable | Unit of analysis |
|---|---|
| Weight | g |
| Nose to tail length | cm |
| Bone mineral density | $g/cm^2$ |
| Bone mineral content | g |
| Lean mass | g |
| Fat mass | g |
| Fat percentage | % |

Abbreviations:
LRT: Likelihood ratio test
ML: Maximum likelihood
REML: Residual maximum likelihood
BMC: Bone mineral content
BMD: Bone mineral density
LM: Lean mass
FM: Fat mass
Fat %: Fat percentage
SE: Standard Error

# Information on diagnostic graphs presented for each model

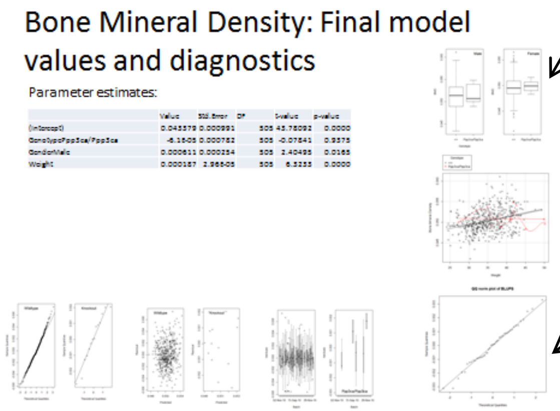

Bone Mineral Density: Final model values and diagnostics

Parameter estimates:

| | Value | Std.Error | DF | t-value | p-value |
|---|---|---|---|---|---|
| (Intercept) | 0.043379 | 0.000991 | 505 | 43.78092 | 0.0000 |
| GenotypePpp3ca/Ppp3ca | -6.1E-05 | 0.000782 | 505 | -0.07841 | 0.9375 |
| GenderMale | 0.000611 | 0.000254 | 505 | 2.40495 | 0.0165 |
| Weight | 0.000187 | 2.96E-05 | 505 | 6.3233 | 0.0000 |

A: A boxplot comparison of the dependent variable for each genotype for each sex.

B: Weight versus dependent variable scatterplot For each genotype a regression and a Loess line is fitted. A Loess line is a locally weighted linear line. Graph only relevant for models that include weight as a fixed effect.

C: Normal Q-Q plot of the best linear unbiased prediction of random effects (BLUPS).

D: For each genotype group, conditional raw residues are plotted versus batch.

E: For each genotype group, a plot of conditional raw residue versus predicted values.

F: Normal Q-Q plots of conditional raw residues for each genotype.

# Mixed Model results 1

Starting model:

$Y_{ij} = \beta_o + \beta_1 Genotype1_{ij} + \beta_2 Sex1_{ij} + \beta_3 Genotype1_{ij} Sex1_{ij} + u_j + e_{ij}$.

# Weight: Top down modelling output

| Hypothesis | Model1 | Model 2 | Test | Estimation method | Test statistic value | *p*-value |
|---|---|---|---|---|---|---|
| Is batch significant? | Batch | No batch | LRT | REML | $\chi^2(0:1)=2.14$ | 0.1434 |
| Is variance homogenous? | Homogenous | Heterogeneous | LRT | REML | $\chi^2(2)=0.01425$ | 0.8366 |
| Testing fixed effects – sex | | | Type 1 *F*-test | REML | $F(1,88)=93.31$ | <0.0001 |
| Testing fixed effects – sex*genotype | | | Type 1 *F*-test | REML | $F(1,88)=0.03$ | 0.8638 |
| Testing treatment - Is genotype significant? | With genotype | Without genotype | LRT | ML | $\chi^2(2)=56.379$ | 5.972e-14 |

# Weight: Final model values and diagnostics

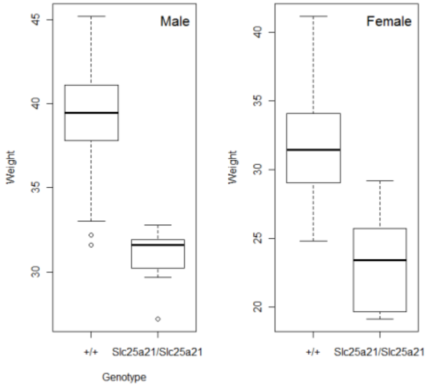


Parameter estimates:

| | Value | Std.Error | t-value | p-value |
|---|---|---|---|---|
| (Intercept) | 31.653 | 0.505 | 62.62795 | 0.000 |
| GenotypeSlc25a21/Slc25a21 | -8.311 | 0.964 | -8.61913 | 0.000 |
| GenderMale | 7.360 | 0.693 | 10.6223 | 0.000 |

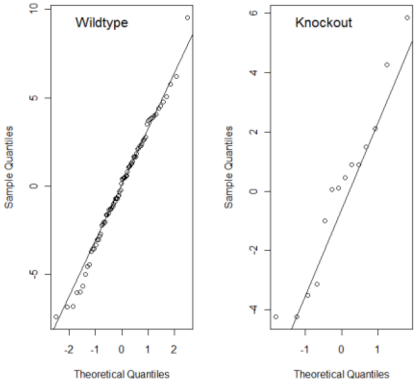


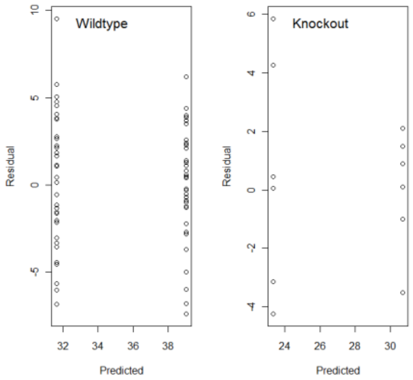


Graph D: N/A as Batch is not significant and thus fitted a linear model

Graph C: N/A as Batch is not significant and thus fitted a linear model

# Nose to tail length: Top down modelling output

| Hypothesis | Model1 | Model 2 | Test | Estimation method | Test statistic value | *p*-value |
|---|---|---|---|---|---|---|
| Is batch significant? | Batch | No batch | LRT | REML | $\chi^2(0:1)= 37.864$ | <.0001 |
| Is variance homogenous? | Homogenous | Heterogeneous | LRT | REML | $\chi^2(2)=0.1713$ | 0.6789 |
| Testing fixed effects – sex | | | Type 1 *F*-test | REML | F(1,73)=5.099 | 0.0000 |
| Testing fixed effects – sex*genotype | | | Type 1 *F*-test | REML | F(1,73)=1.94336 | 0.0558 |
| Testing treatment - Is genotype significant? | With genotype | Without genotype | LRT | ML | $\chi^2(2)=11.467$ | 0.0007083 22 |

# Nose to tail length: Final model values and diagnostics

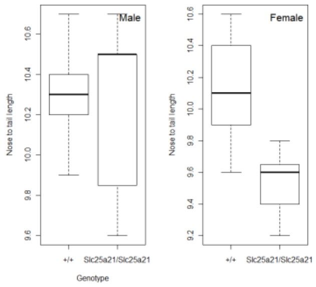


Parameter estimates:

| | Value | Std.Error | DF | t-value | p-value |
|---|---|---|---|---|---|
| (Intercept) | 10.078 | 0.071 | 74 | 141.744 | 0.0000 |
| GenotypeSlc25a21/Slc25a21 | -0.363 | 0.105 | 74 | -3.473 | 0.0009 |
| GenderMale | 0.262 | 0.044 | 74 | 5.912 | 0.0000 |

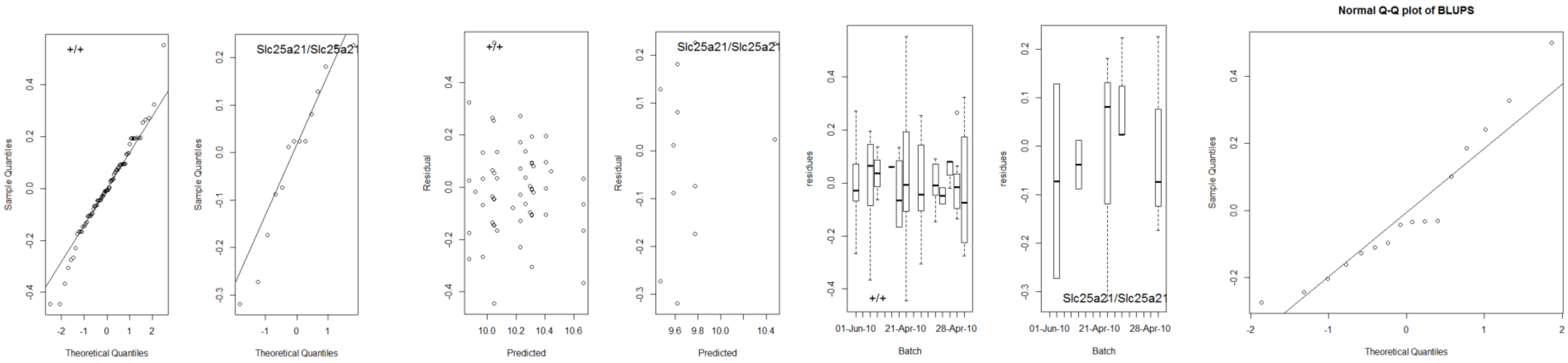

# Bone mineral Density:
# Top down modelling output

| Hypothesis | Model1 | Model 2 | Test | Estimation method | Test statistic value | *p*-value |
|---|---|---|---|---|---|---|
| Is batch significant? | Batch | No batch | LRT | REML | $\chi^2(0:1)=14.8039$ | 1e-4 |
| Is variance homogenous? | Homogenous | Heterogeneous | LRT | REML | $\chi^2(2)=0.3329$ | 0.5639 |
| Testing fixed effects – sex | | | Type 1 F-test | REML | F(1,65)=1.774 | 0.0807 |
| Testing fixed effect – genotype*sex | | | Type 1 F-test | REML | F(1,65)= 1.653 | 0.103 |
| Testing treatment - Is genotype significant? | With genotype | Without genotype | LRT | ML | $\chi^2(2)=8.202$ | 0.0041 82 |

# Bone Mineral Density: Final model values and diagnostics

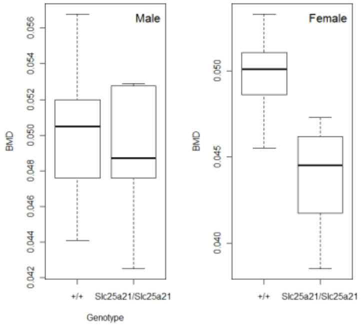


Parameter estimates:

| | Value | Std.Error | DF | t-value | p-value |
|---|---|---|---|---|---|
| (Intercept) | 0.050 | 0.001 | 67 | 72.492 | 0.0000 |
| GenotypeSlc25a21/Slc25a21 | -0.003 | 0.001 | 67 | -2.938 | 0.0045 |

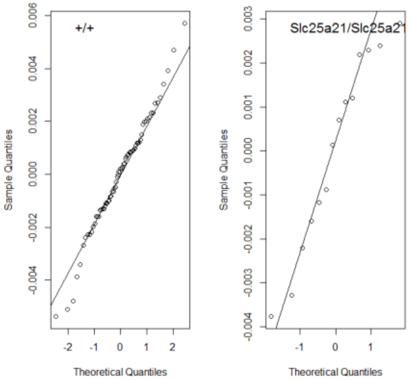


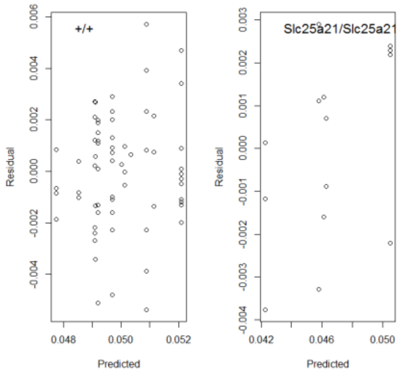


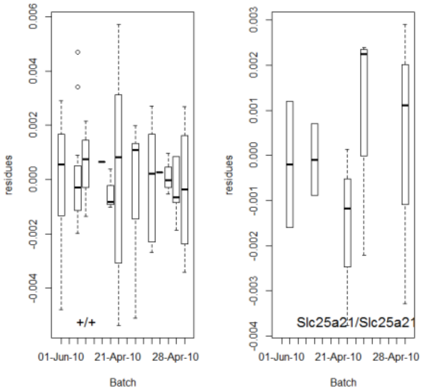


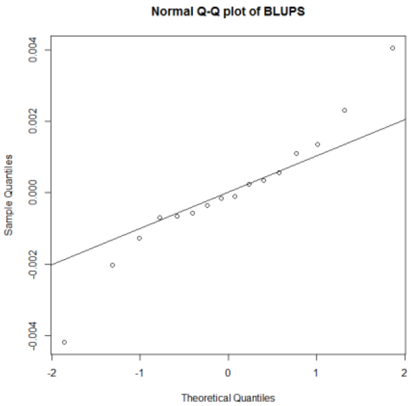

# Bone Mineral Content: Top down modelling output

| Hypothesis | Model1 | Model 2 | Test | Estimation method | Test statistic value | *p*-value |
|---|---|---|---|---|---|---|
| Is batch significant? | Batch | No batch | LRT | REML | $\chi^2(0{:}1)=5.630$ | 0.0177 |
| Is variance homogenous? | Homogenous | Heterogeneous | LRT | REML | $\chi^2(2)=1.407$ | 0.2354 |
| Testing fixed effects – sex | | | Type 1 *F*-test | REML | F(1,65)=4.452 | 0.0000 |
| Testing fixed effect – genotype*sex | | | Type 1 *F*-test | REML | F(1,65)=1.955 | 0.0548 |
| Testing treatment - Is genotype significant? | With genotype | Without genotype | LRT | ML | $\chi^2(2)=12.92$ | 0.00032 428 |

# Bone Mineral Content: Final model values and diagnostics

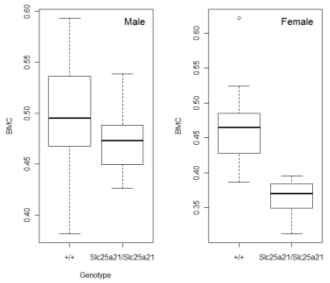


Parameter estimates:

| | Value | Std.Error | DF | t-value | p-value |
|---|---|---|---|---|---|
| (Intercept) | 0.454 | 0.010 | 66 | 46.005 | 0.0000 |
| GenotypeSlc25a21/Slc25a21 | -0.066 | 0.017 | 66 | -3.842 | 0.0003 |
| GenderMale | 0.057 | 0.010 | 66 | 5.592 | 0.0000 |

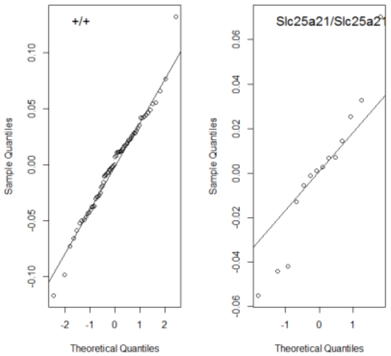


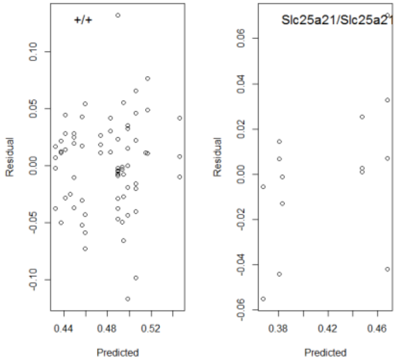


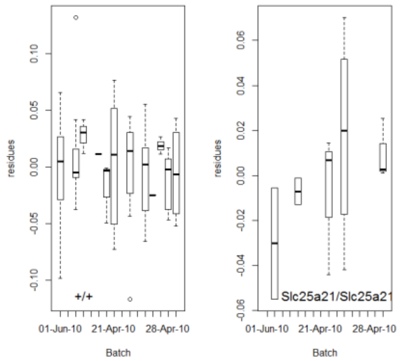


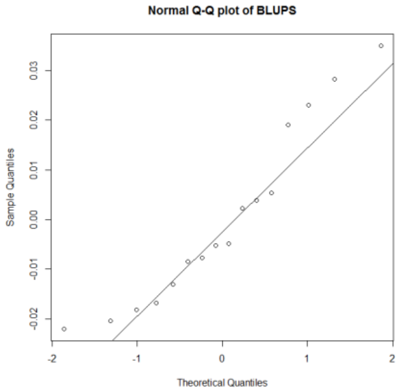

# Lean Mass:
# Top down modelling output

| Hypothesis | Model1 | Model 2 | Test | Estimation method | Test statistic value | *p*-value |
|---|---|---|---|---|---|---|
| Is batch significant? | Batch | No batch | LRT | REML | $\chi^2(0{:}1)=6.044$ | 0.014 |
| Is variance homogenous? | Homogenous | Heterogeneous | LRT | REML | $\chi^2(2)=3.128$ | 0.077 |
| Testing fixed effects – sex | | | Type 1 *F*-test | REML | F(1,65)=10.9555 | 0.0000 |
| Testing fixed effect – genotype*sex | | | Type 1 *F*-test | REML | F(1,65)=0.879 | 0.3826 |
| Testing treatment - Is genotype significant? | With genotype | Without genotype | LRT | ML | $\chi^2(2)=16.823$ | 4.10e-05 |

# Lean Mass: Final model values and diagnostics

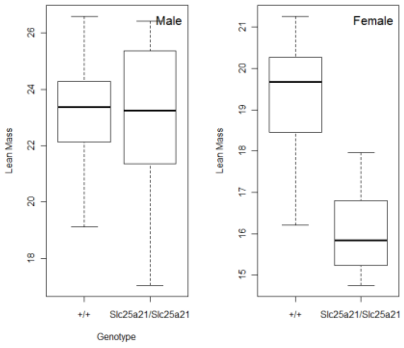


Parameter estimates:

| | Value | Std.Error | DF | t-value | p-value |
|---|---|---|---|---|---|
| (Intercept) | 19.528 | 0.438 | 66 | 44.573 | 0.0000 |
| GenotypeSlc25a21/Slc25a21 | -3.041 | 0.709 | 66 | -4.288 | 0.0001 |
| GenderMale | 4.355 | 0.360 | 66 | 12.081 | 0.0000 |

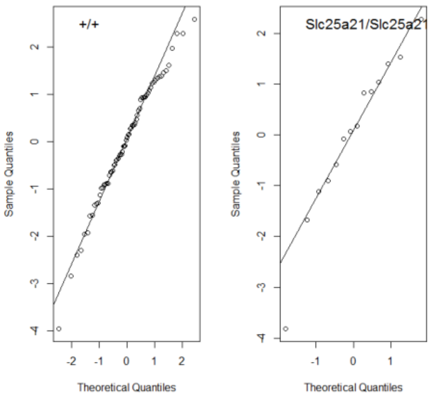


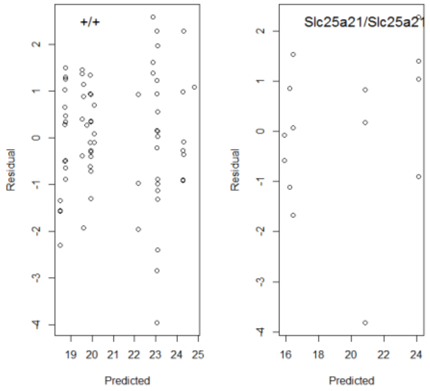


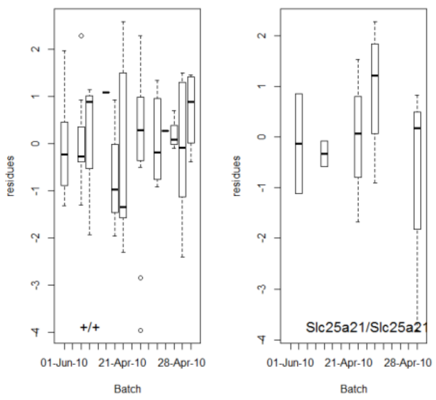


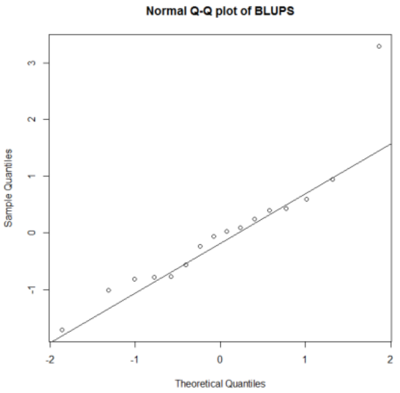

# Fat Mass: Final model values and diagnostics

| Hypothesis | Model1 | Model 2 | Test | Estimation method | Test statistic value | *p*-value |
|---|---|---|---|---|---|---|
| Is batch significant? | Batch | No batch | LRT | REML | $\chi^2(0:1)=1.3965$ | 0.2373 |
| Is variance homogenous? | Homogenous | Heterogeneous | LRT | REML | $\chi^2(2)=0.148$ | 0.7001 |
| Testing fixed effects – sex | | | Type 1 F-test | REML | F(1,65)=4.77 | 0.0000 |
| Testing fixed effect – genotype*sex | | | Type 1 F-test | REML | F(1,65)=-1.4633 | 0.1473 |
| Testing treatment - Is genotype significant? | With genotype | Without genotype | LRT | ML | $\chi^2(2)=43.555$ | 4.12e-11 |

# Fat Mass: Final model values and diagnostics

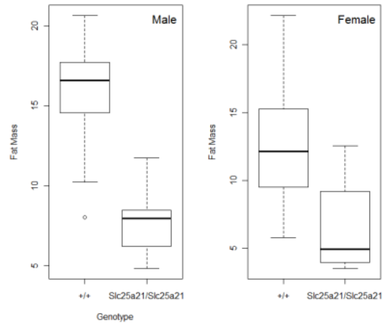


Parameter estimates:

| | Value | Std.Error | t-value | p-value |
|---|---|---|---|---|
| (Intercept) | 12.558 | 0.485 | 25.92002 | 0.000 |
| GenotypeSlc25a21/Slc25a21 | -6.922 | 0.933 | -7.41908 | 0.000 |
| GenderMale | 3.185 | 0.700 | 4.547962 | 0.000 |

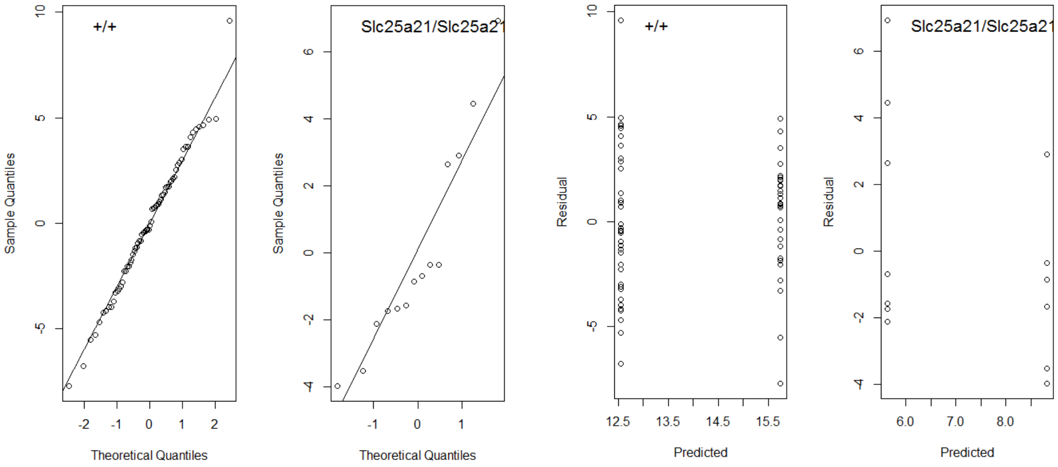


Graph C: N/A as Batch is not significant and thus fitted a linear model

Graph D: N/A as Batch is not significant and thus fitted a linear model

# Dependent variable: Fat Percentage (Fat %)

| Hypothesis | Model1 | Model 2 | Test | Estimation method | Test statistic value | *p*-value |
|---|---|---|---|---|---|---|
| Is batch significant? | Batch | No batch | LRT | REML | $\chi^2$(0:1)=3.3895 | 0.0656 |
| Is variance homogenous? | Homogenous variance | Heterogeneous | LRT | REML | $\chi^2$(2)=2.417 | 0.12 |
| Testing fixed effects – sex | | | Type 1 F-test | REML | F(1,65)=4.776 | 0.0000 |
| Testing fixed effect – genotype*sex | | | Type 1 F-test | REML | F(1,65)=-1.463 | 0.1473 |
| Is genotype significant? | With genotype | Without genotype | LRT | ML | $\chi^2$(2)=9.6035 | 0.00194 |

# Fat %: Final model values and diagnostics

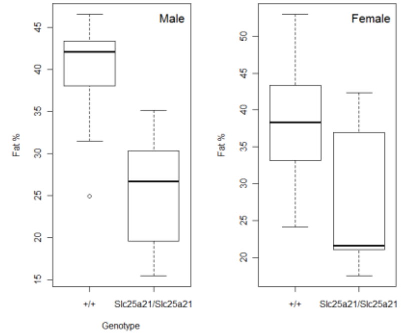


Parameter estimates:

| | Value | Std.Error | DF | t-value | p-value |
|---|---|---|---|---|---|
| (Intercept) | 39.008 | 1.346 | 67 | 28.974 | 0.0000 |
| GenotypeSlc25a21/Slc25a21 | -9.983 | 2.450 | 67 | -4.075 | 0.0001 |

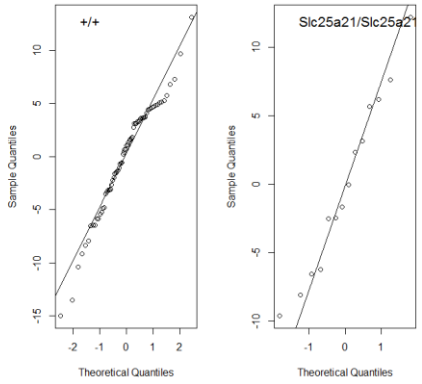


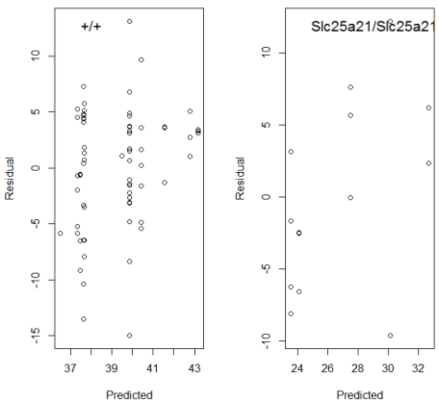


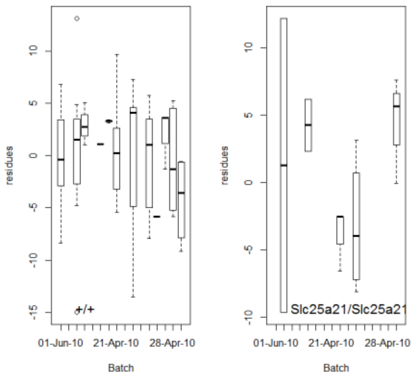


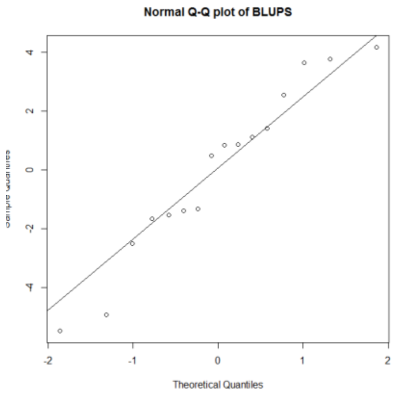

# Summary

| Variable | *p* value | Adjusted *p*' value | Genotype Estimate ± SE | Sex |
|---|---|---|---|---|
| Weight | 5.9e-14 | 6.14E-13 | Y↓ -8.31±0.96 | Y↑ |
| Nose to tail length | 0.0007083 | 0.001754 | Y↓ -0.315±0.1464 | Y↑ |
| BMD | 0.004182 | 0.007531 | Y↓ -0.003±0.001 | N |
| BMC | 0.004182 | 0.007531 | Y↓ -0.066±0.0017 | Y↑ |
| LM | 4.1e-5 | 0.000119 | Y↓ -3.041±0.709 | Y↑ |
| FM | 4.12e-11 | 3.06E-10 | Y↓ -6.922±0.933 | Y↑ |
| Fat % | 0.00194 | 0.004386 | Y↓ -9.983±2.450 | N |

Y denotes a statistically significant effect and N indicates a non significant effect. The ↑ symbol indicates a positive estimated regression coefficient such that this effect leads to an increase in the dependent variable. Whilst, the ↓ symbol indicates a negative estimated regression coefficient such that this effect leads to a decrease in the dependent variable.

# Mixed Model results 2

Starting model:

$$Y_{ij} = \beta_o + \beta_1 Genotype1_{ij} + \beta_2 Sex1_{ij} + \beta_3 Weight1_{ij\,+}\, \beta_4 Genotype1_{ij} Sex1_{ij} + u_j + e_{ij}.$$

# Nose to tail length:
# Top down modelling output

| Hypothesis | Model1 | Model 2 | Test | Estimation method | Test statistic value | *p*-value |
|---|---|---|---|---|---|---|
| Is batch significant? | Batch | No batch | LRT | REML | $\chi^2(0:1)= 41.44$ | <0.0001 |
| Is variance homogenous? | Homogenous | Heterogeneous | LRT | REML | $\chi^2(2)=8.54e\text{-}6$ | 0.9977 |
| Testing fixed effects – sex | | | Type 1 *F*-test | REML | F(1,72)=0.1581 | 0.6921 |
| Testing fixed effects – sex*genotype | | | Type 1 *F*-test | REML | F(1,72)=5.14 | 0.0265 |
| Testing fixed effects – Weight | | | Type 1 *F*-test | REML | F(1,72)=34.62 | <0.0001 |
| Testing treatment - Is genotype significant? | With genotype | Without genotype | LRT | ML | $\chi^2(2)=5.52$ | 0.0631 |

# Nose to tail length: Final model values and diagnostics

Parameter estimates:

| | Value | Std.Error | DF | t-value | p-value |
|---|---|---|---|---|---|
| (Intercept) | 9.059862 | 0.189361 | 72 | 47.84442 | 0.0000 |
| GenotypeSlc25a21/Slc25a21 | -0.31514 | 0.146466 | 72 | -2.15166 | 0.0348 |
| sexMale | -0.02349 | 0.059063 | 72 | -0.39767 | 0.6921 |
| Weight | 0.032972 | 0.005603 | 72 | 5.8842 | 0.0000 |
| GenotypeSlc25a21/Slc25a21:sexMale | 0.389204 | 0.171751 | 72 | 2.2661 | 0.0265 |

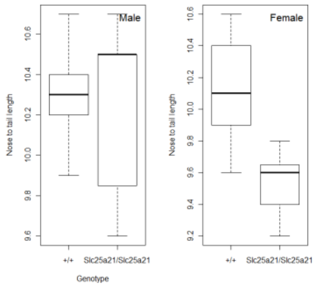


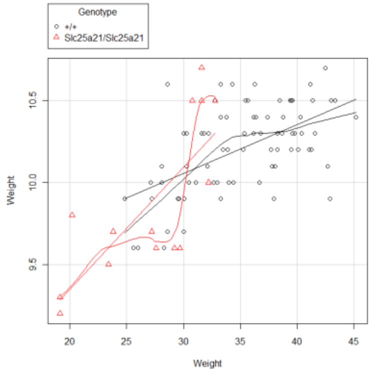


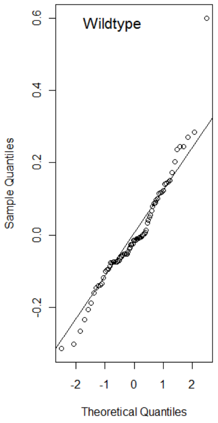


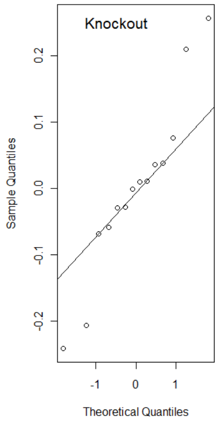


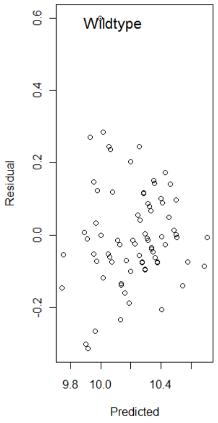


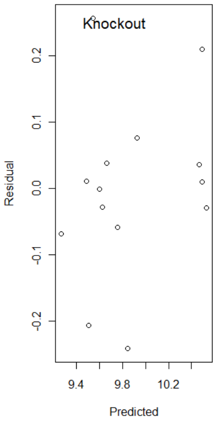


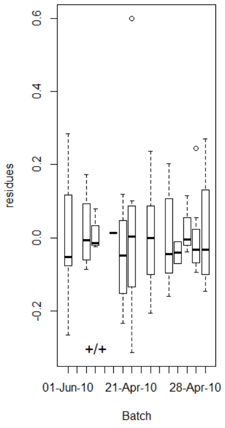


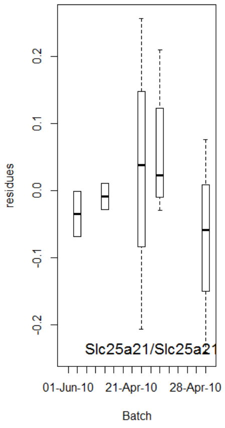


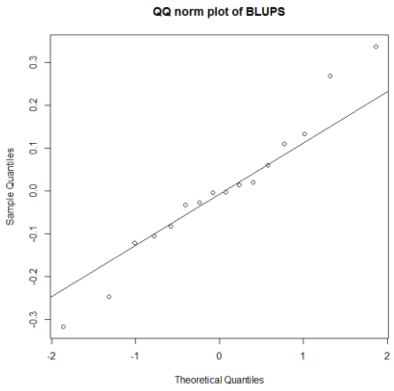

# Bone mineral Density: Top down modelling output

| Hypothesis | Model1 | Model 2 | Test | Estimation method | Test statistic value | *p*-value |
|---|---|---|---|---|---|---|
| Is batch significant? | Batch | No batch | LRT | REML | $\chi^2(0:1)=10.142$ | 0.0014 |
| Is variance homogenous? | Homogenous | Heterogeneous | LRT | REML | $\chi^2(2)=0.04425$ | 0.8334 |
| Testing fixed effects – sex | | | Type 1 F-test | REML | F(1,64)=0.6274 | 0.4312 |
| Testing fixed effects – weight | | | Type 1 F-test | REML | F(1,64)=7.197 | 0.0093 |
| Testing fixed effect – genotype*sex | | | Type 1 F-test | REML | F(1,64)= 3.913 | 0.0522 |
| Testing treatment - Is genotype significant? | With genotype | Without genotype | LRT | ML | $\chi^2(2)=3.929$ | 0.0474 |

# Bone Mineral Density: Final model values and diagnostics

Parameter estimates:

| | Value | Std.Error | DF | t-value | p-value |
|---|---|---|---|---|---|
| (Intercept) | 0.04332 | 0.001898 | 66 | 22.82316 | 0.0000 |
| GenotypeSlc25a21/Slc25a21 | -0.00215 | 0.001092 | 66 | -1.96615 | 0.0535 |
| Weight | 0.000187 | 5.2E-05 | 66 | 3.602649 | 0.0006 |

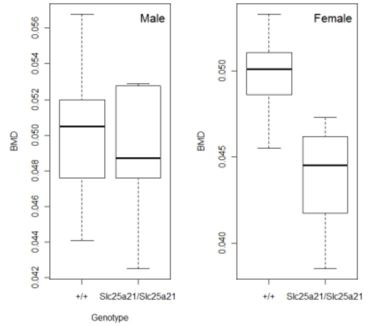


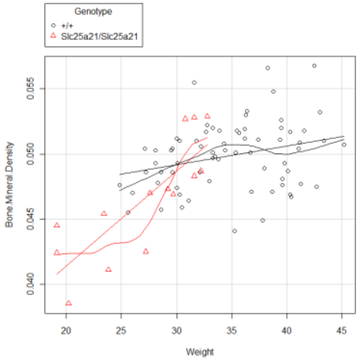


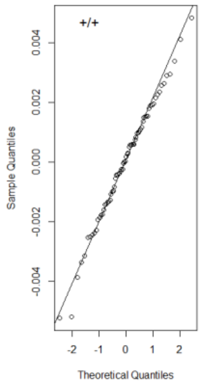


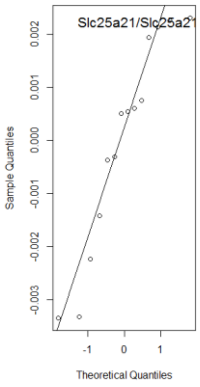


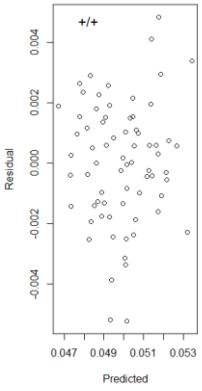


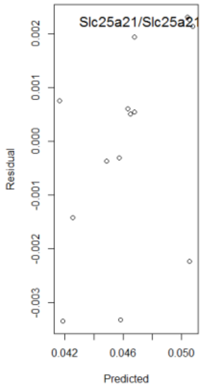


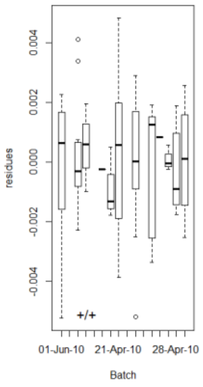


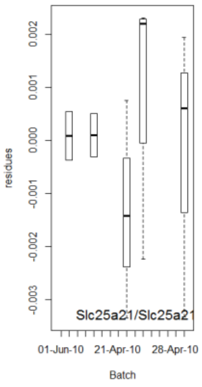


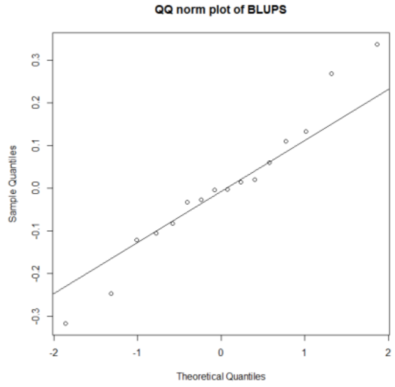

# Bone Mineral Content: Top down modelling output

| Hypothesis | Model1 | Model 2 | Test | Estimation method | Test statistic value | *p*-value |
|---|---|---|---|---|---|---|
| Is batch significant? | Batch | No batch | LRT | REML | $\chi^2(0:1)=2.9729$ | 0.0847 |
| Is variance homogenous? | Homogenous | Heterogeneous | LRT | REML | $\chi^2(2)=0.4284$ | 0.5128 |
| Testing fixed effects – sex | | | Type 1 *F*-test | REML | F(1,64)=0.2382 | 0.6272 |
| Testing fixed effects – weight | | | Type 1 *F*-test | REML | F(1,64)=29.26 | <0.0001 |
| Testing fixed effect – genotype*sex | | | Type 1 *F*-test | REML | F(1,64)=6.22 | 0.0152 |
| Testing treatment - Is genotype significant? | With genotype | Without genotype | LRT | ML | $\chi^2(2)=6.55$ | 0.0377 |

# Bone Mineral Content: Final model values and diagnostics

Parameter estimates:

| | Value | Std.Error | DF | t-value | p-value |
|---|---|---|---|---|---|
| (Intercept) | 0.238608 | 0.041487 | 64 | 5.751442 | 0.0000 |
| GenotypeSlc25a21/Slc25a21 | -0.03743 | 0.02113 | 64 | -1.77151 | 0.0812 |
| sexMale | -0.00669 | 0.013714 | 64 | -0.48808 | 0.6272 |
| Weight | 0.006947 | 0.001284 | 64 | 5.409243 | 0.0000 |
| GenotypeSlc25a21/Slc25a21:sexMale | 0.064314 | 0.025783 | 64 | 2.494472 | 0.0152 |

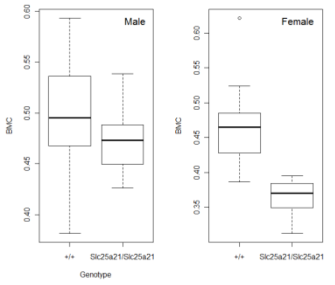


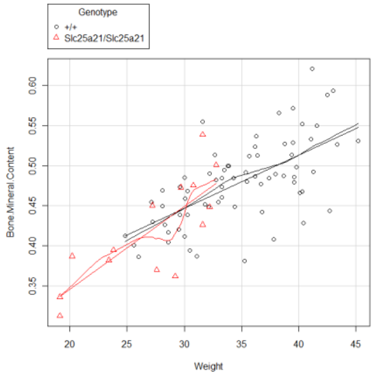


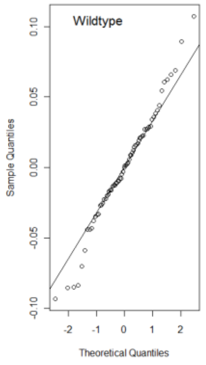


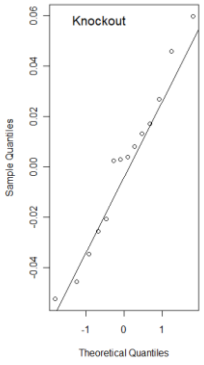


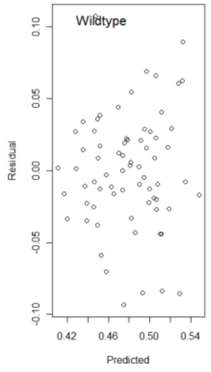


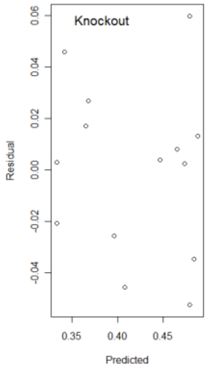


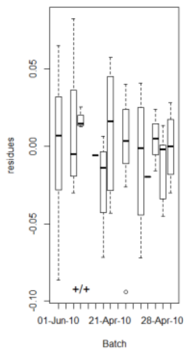


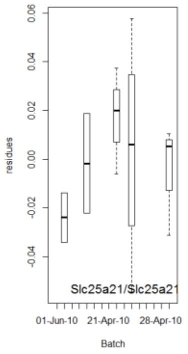


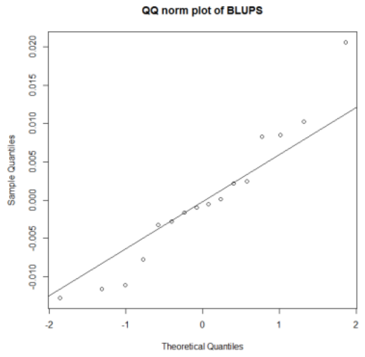

# Lean Mass:
# Top down modelling output

| Hypothesis | Model1 | Model 2 | Test | Estimation method | Test statistic value | *p*-value |
|---|---|---|---|---|---|---|
| Is batch significant? | Batch | No batch | LRT | REML | $\chi^2(0{:}1)=5.8455$ | 0.0156 |
| Is variance homogenous? | Homogenous | Heterogeneous | LRT | REML | $\chi^2(2)=2.557$ | 0.1098 |
| Testing fixed effects – sex | | | Type 1 *F*-test | REML | F(1,64)=24.07 | <0.0001 |
| Testing fixed effects – weight | | | Type 1 *F*-test | REML | F(1,64)=25.19 | <0.0001 |
| Testing fixed effect – genotype*sex | | | Type 1 *F*-test | REML | F(1,64)=0.595 | 0.4435 |
| Testing treatment - Is genotype significant? | With genotype | Without genotype | LRT | ML | $\chi^2(2)=1.979$ | 0.1595 |

# Lean Mass: Final model values and diagnostics

Parameter estimates:

| | Value | Std.Error | DF | t-value | p-value |
|---|---|---|---|---|---|
| (Intercept) | 12.25745 | 1.508994 | 65 | 8.122927 | 0.0000 |
| GenotypeSlc25a21/Slc25a21 | -1.18743 | 0.766222 | 65 | -1.54972 | 0.1261 |
| sexMale | 2.529931 | 0.478779 | 65 | 5.284128 | 0.0000 |
| Weight | 0.228928 | 0.045295 | 65 | 5.054133 | 0.0000 |

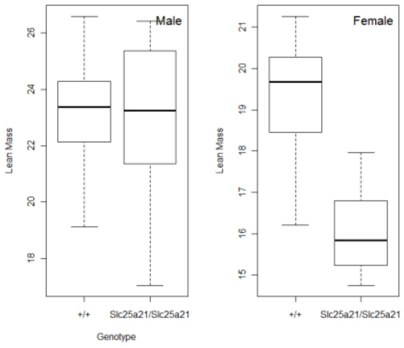


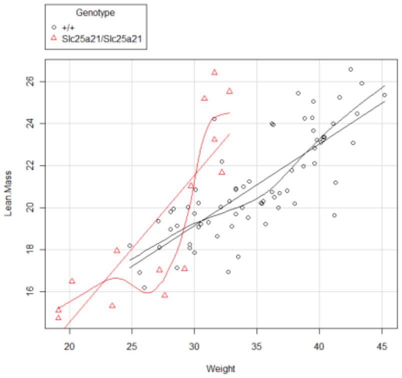


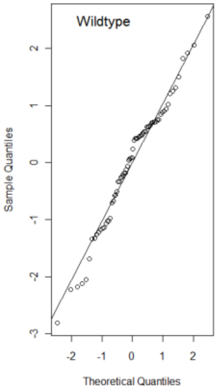


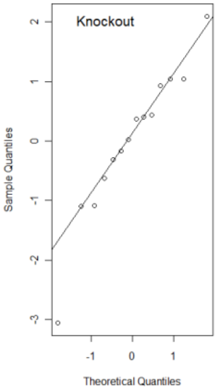


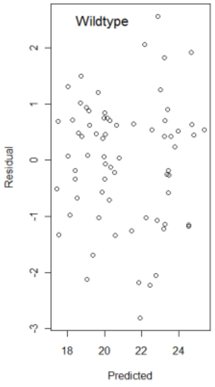


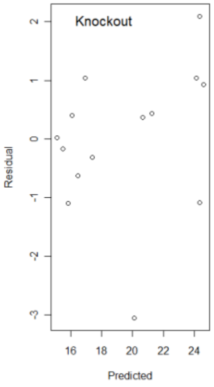


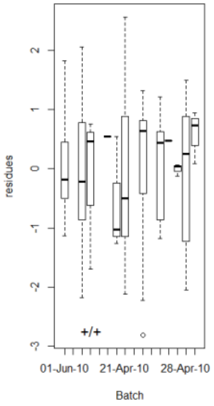


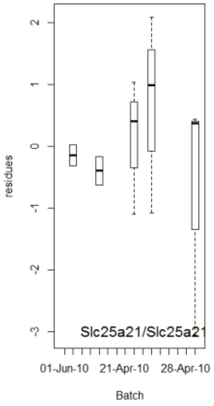


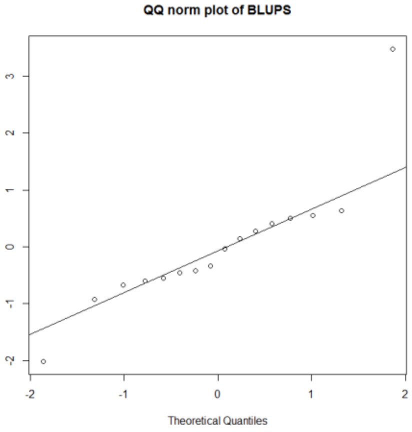

# Fat Mass: Final model values and diagnostics

| Hypothesis | Model1 | Model 2 | Test | Estimation method | Test statistic value | *p*-value |
|---|---|---|---|---|---|---|
| Is batch significant? | Batch | No batch | LRT | REML | $\chi^2(0{:}1)=6.51$ | 0.0107 |
| Is variance homogenous? | Homogenous | Heterogeneous | LRT | REML | $\chi^2(2)=1.45$ | 0.2284 |
| Testing fixed effects – sex | | | Type 1 F-test | REML | $F(1,64)=23.97$ | $<0.0001$ |
| Testing fixed effects – weight | | | Type 1 F-test | REML | $F(1,64)=290.50$ | $<0.0001$ |
| Testing fixed effect – genotype*sex | | | Type 1 F-test | REML | $F(1,64)=0.397$ | 0.5308 |
| Testing treatment - Is genotype significant? | With genotype | Without genotype | LRT | ML | $\chi^2(2)=2.10$ | 0.1472 |

# Fat Mass: Final model values and diagnostics

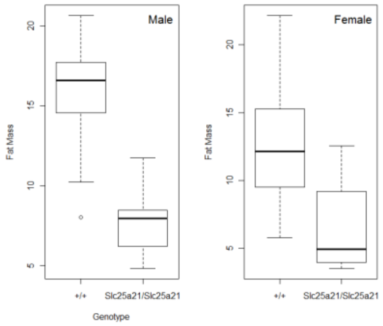


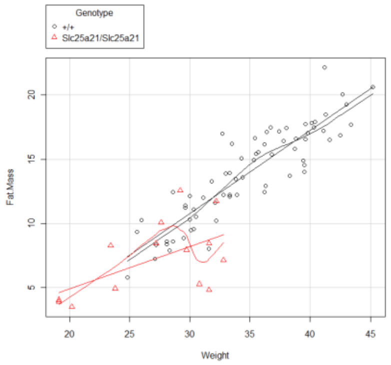


## Parameter estimates:

| | Value | Std.Error | DF | t-value | p-value |
|---|---|---|---|---|---|
| (Intercept) | -13.6538 | 1.574434 | 65 | -8.67223 | 0.0000 |
| GenotypeSlc25a21/Slc25a21 | 1.290189 | 0.801025 | 65 | 1.610673 | 0.1121 |
| sexMale | -2.60884 | 0.499432 | 65 | -5.22362 | 0.0000 |
| Weight | 0.813766 | 0.047237 | 65 | 17.22727 | 0.0000 |

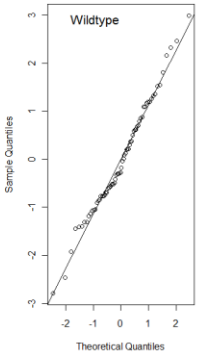


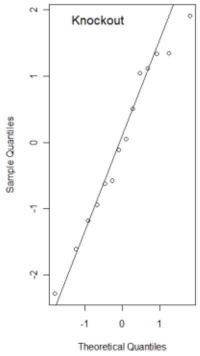


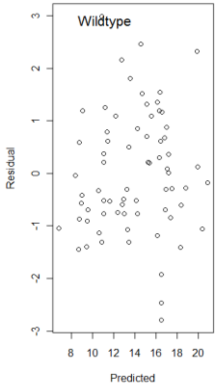


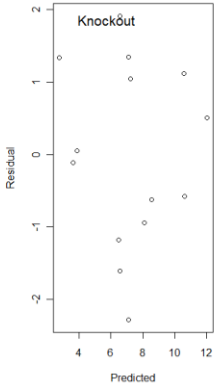


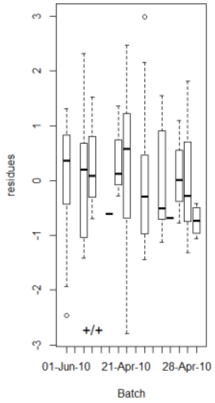


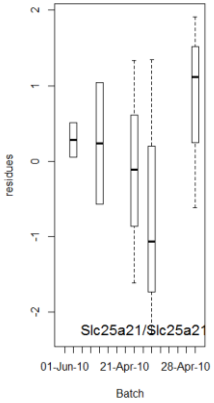


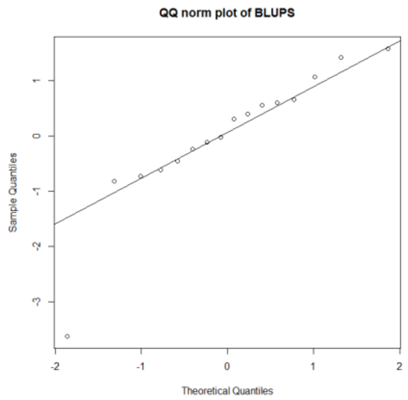

# Dependent variable: Fat Percentage (Fat %)

| Hypothesis | Model1 | Model 2 | Test | Estimation method | Test statistic value | *p*-value |
|---|---|---|---|---|---|---|
| Is batch significant? | Batch | No batch | LRT | REML | $\chi^2(0:1)=8.414$ | 0.0037 |
| Is variance homogenous? | Homogenous variance | Heterogeneous | LRT | REML | $\chi^2(2)=3.89$ | 0.0486 |
| Testing fixed effects – sex | | | Type 1 F-test | REML | F(1,64)=25.58 | 0.012 |
| Testing fixed effects – weight | | | Type 1 F-test | REML | F(1,64)= 82.31 | <0.0001 |
| Testing fixed effect – genotype*sex | | | Type 1 F-test | REML | F(1,64)=0.781 | 0.3800 |
| Is genotype significant? | With genotype | Without genotype | LRT | ML | $\chi^2(2)=0.1646$ | 0.6849 |

# Fat %: Final model values and diagnostics

Parameter estimates:

| | Value | Std.Error | DF | t-value | p-value |
|---|---|---|---|---|---|
| (Intercept) | -1.60846 | 4.495582 | 65 | -0.35779 | 0.7217 |
| GenotypeSlc25a21/Slc25a21 | -0.74944 | 2.510662 | 65 | -0.2985 | 0.7663 |
| sexMale | -7.60777 | 1.439781 | 65 | -5.28398 | 0.0000 |
| Weight | 1.257946 | 0.13849 | 65 | 9.083269 | 0.0000 |

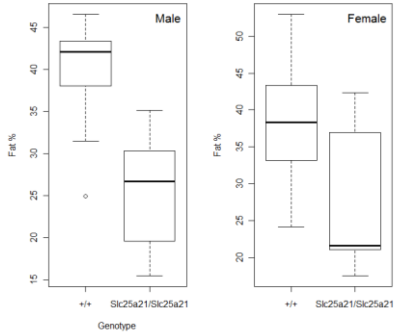


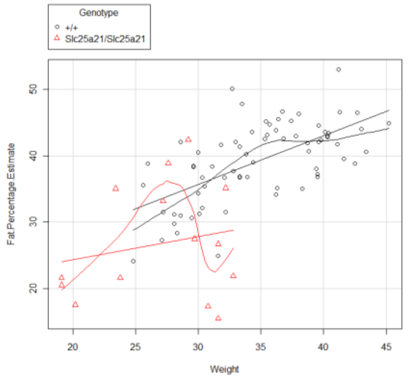


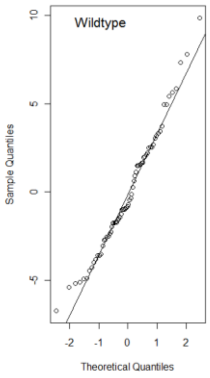


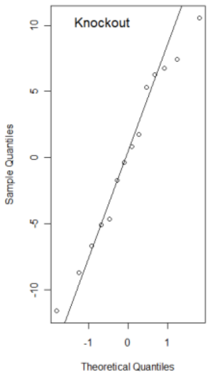


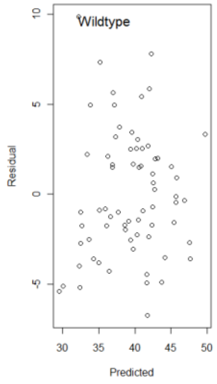


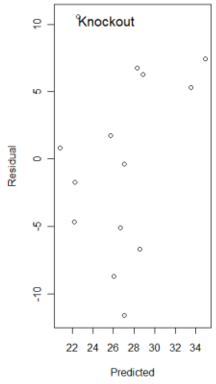


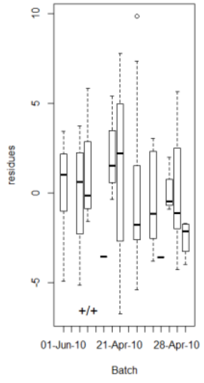


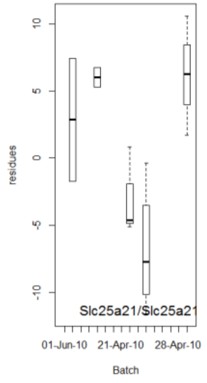


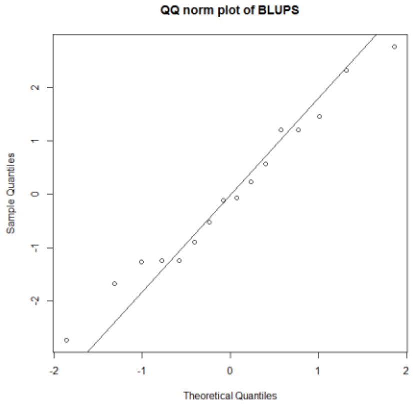

# Summary

| Variable | *p*-value | Adjusted *p*' value | Genotype Estimate ± SE | Sex | Weight |
|---|---|---|---|---|---|
| Nose to tail length | 0.0631 | 0.0911 | N | N | Y↑ |
| BMD | 0.0474 | 0.0704 | N | N | Y↑ |
| BMC | 0.0377 | 0.0576 | N | Y↓ | Y↑ |
| LM | 0.1594 | 0.2021 | N | Y↑ | Y↑ |
| FM | 0.1472 | 0.1913 | N | Y↓ | Y↑ |
| Fat % | 0.6849 | 0.6983 | N | Y↓ | Y↑ |

Y denotes a statistically significant effect and N indicates a non significant effect. The ↑ symbol indicates a positive estimated regression coefficient such that this effect leads to an increase in the dependent variable. Whilst, the ↓ symbol indicates a negative estimated regression coefficient such that this effect leads to a decrease in the dependent variable.
