# Supplementary material for: Robust and Sensitive Analysis of Mouse Knockout Phenotypes
Source: PLoS One. 2012 Dec 26;7(12):e52410. doi: 10.1371/journal.pone.0052410 (PMC3530558; doi:10.1371/journal.pone.0052410)
Supplement: File S4 — Detailed mixed model output for the allele Cenpjtm1a(EUCOMM)Wtsi and associated DEXA data. Legend: For each trait studied, for each model fitting procedures, the final model output was captured and the data visualised with a boxplot. Furthermore, to test the quality of model fit, a number of graphical diagnostic plots were generated for each gene and trait. (PDF) [file pone.0052410.s007.pdf]

Cenpj$^{tm1a(EUCOMM)Wtsi}$

DEXA mixed model analysis

Variable	Unit of analysis
Weight	g
Nose to tail length	cm
Bone mineral density	g/cm^2
Bone mineral content	g
Lean mass	g
Fat mass	g
Fat percentage	%

Abbreviations:
LRT: Likelihood ratio test
ML: Maximum likelihood
REML: Residual maximum likelihood
BMC: Bone mineral content
BMD: Bone mineral density
LM: Lean mass
FM: Fat mass
Fat %: Fat percentage
SE: Standard Error

Information on diagnostic graphs presented for each model

A: A boxplot comparison of the dependent variable for each genotype for each sex.

B: Weight versus dependent variable scatterplot For each genotype a regression and a Loess line is fitted. A Loess line is a locally weighted linear line.

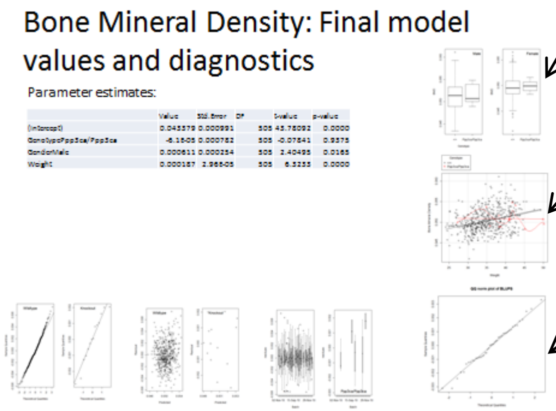
Bone Mineral Density: Final model values and diagnostics

Parameter estimates:

	Value	Std.Error	DF	t-value	p-value
(Intercept)	0.045379	0.000991	505	45.78092	0.0000
GenotypePpp3ca/Ppp3ca	-6.1E-05	0.000782	505	-0.07841	0.9375
GenderMale	0.000611	0.000254	505	2.40495	0.0165
Weight	0.000187	2.96E-05	505	6.3233	0.0000

C: Normal Q-Q plot of the best linear unbiased prediction of random effects (BLUPS).

D: For each genotype group, conditional raw residues are plotted versus batch.

E: For each genotype group, a plot of conditional raw residue versus predicted values.

F: Normal Q-Q plots of conditional raw residues for each genotype.

Mixed Model results 1

Starting model:

$$Y_{ij} = \beta_o + \beta_1 Genotype1_{ij} + \beta_2 Sex1_{ij} + \beta_3 Genotype1_{ij} Sex1_{ij} + u_j + e_{ij}.$$

Weight: Top down modelling output

Hypothesis	Model1	Model 2	Test	Estimation method	Test statistic value	*p*-value
Is batch significant?	Batch	No batch	LRT	REML	$\chi^2(0:1)=26.96$	<.0001
Is variance homogenous?	Homogenous	Heterogeneous	LRT	REML	$\chi^2(2)=0.36$	0.5496
Testing fixed effects – sex			Type 1 *F*-test	REML	$F(1,371)=349.41$	<.0001
Testing fixed effects – sex*genotype			Type 1 *F*-test	REML	$F(1,371)=0.091$	0.7628
Testing treatment - Is genotype significant?	With genotype	Without genotype	LRT	ML	$\chi^2(2)=85.133$	0.0000000

Weight: Final model values and diagnostics

Parameter estimates:

	Value	Std.Error	DF	t-value	p-value
(Intercept)	32.20305	0.333466	372	96.57072	0.0000
GenotypeCenpj/Cenpj	-12.7492	1.06218	372	-12.0029	0.0000
sexMale	6.15093	0.322663	372	19.06303	0.0000

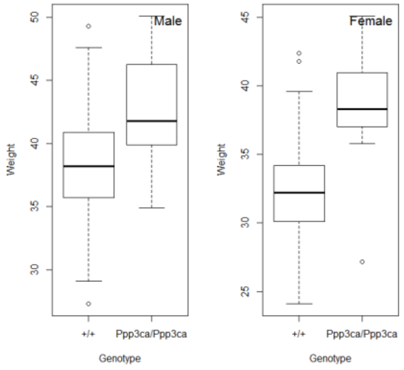

Graph B: N/A for weight as dependent variable

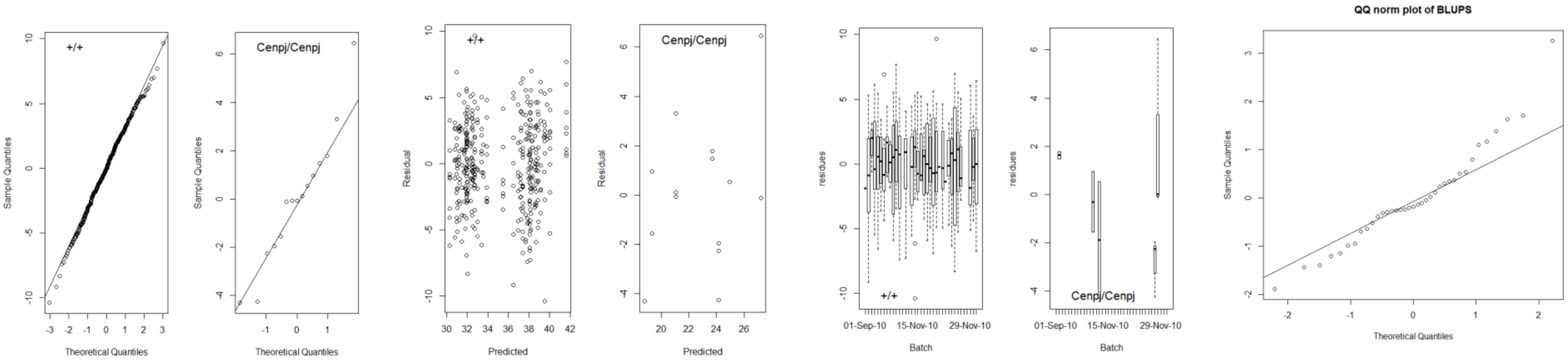

Nose to tail length:
Top down modelling output

Hypothesis	Model1	Model 2	Test	Estimation method	Test statistic value	*p*-value
Is batch significant?	Batch	No batch	LRT	REML	$\chi^2(0:1)=180.76$	<0.0001
Is variance homogenous?	Homogenous	Heterogeneous	LRT	REML	$\chi^2(2)=4.15$	0.0417
Testing fixed effects – sex			Type 1 *F*-test	REML	F(1,369)=15.34	0.0000
Testing fixed effects – sex*genotype			Type 1 *F*-test	REML	F(1,369)=1.680	0.0937
Testing treatment - Is genotype significant?	With genotype	Without genotype	LRT	ML	$\chi^2(2)=81.89$	0.000000

Nose to tail length: Final model values and diagnostics

Parameter estimates:

	Value	Std.Error	DF	t-value	p-value
(Intercept)	10.06	0.03	370	298.87	0.0000
GenotypeCenpj/Cenpj	-2.43	0.07	370	-34.17	0.0000
GenderMale	0.31	0.02	370	16.13	0.0000

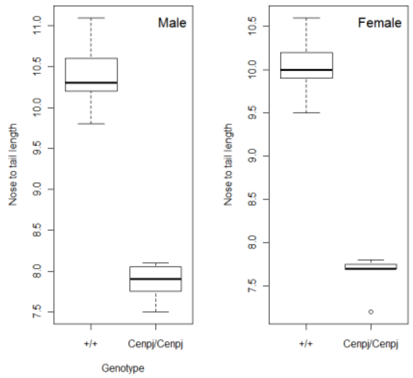

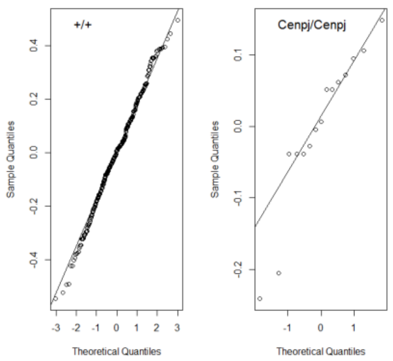

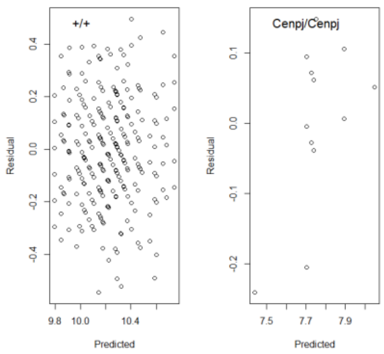

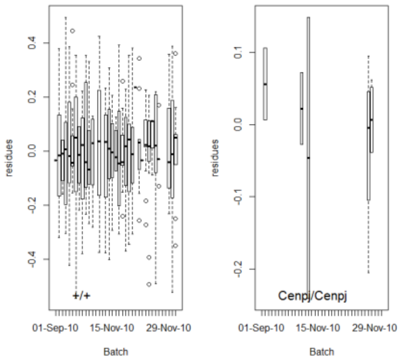

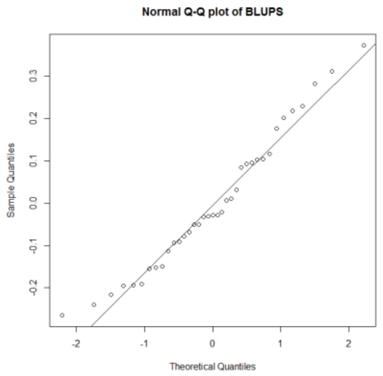

Bone mineral Density:
Top down modelling output

Hypothesis	Model1	Model 2	Test	Estimation method	Test statistic value	*p*-value
Is batch significant?	Batch	No batch	LRT	REML	$\chi^2(0:1)=64.08$	<0.0001
Is variance homogenous?	Homogenous	Heterogeneous	LRT	REML	$\chi^2(2)=0.74$	0.3894
Testing fixed effects – sex			Type 1 F-test	REML	$F(1,359)=7.52$	0.0000
Testing fixed effect – genotype*sex			Type 1 F-test	REML	$F(1,359)=1.41$	0.1592
Testing treatment - Is genotype significant?	With genotype	Without genotype	LRT	ML	$\chi^2(2)=5.109$	0.0238

Bone Mineral Density: Final model values and diagnostics

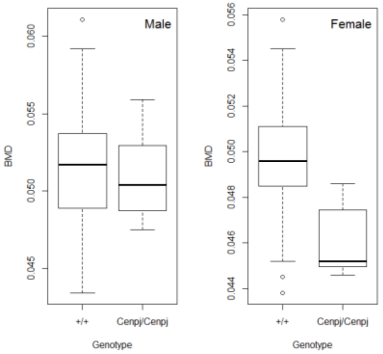

Parameter estimates:

	Value	Std.Error	DF	t-value	p-value
(Intercept)	0.05	0.00	360	149.78	0.0000
GenotypeCenpj/Cenpj	0.00	0.00	360	-2.25	0.0249
GenderMale	0.00	0.00	360	7.88	0.0000

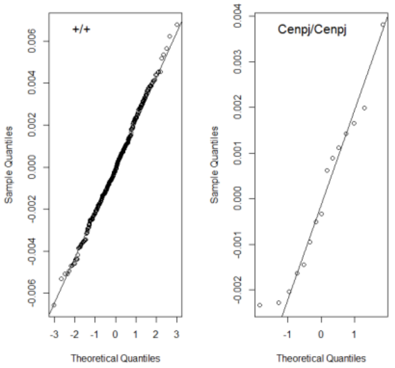

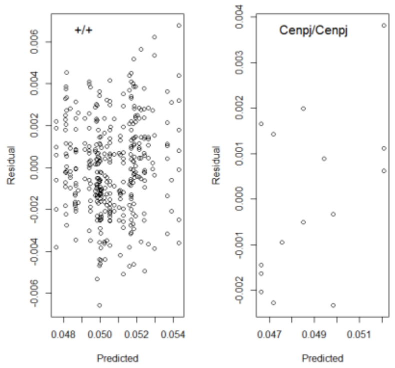

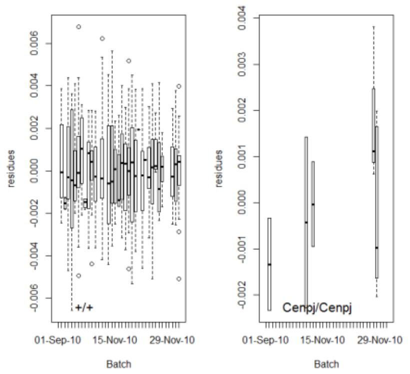

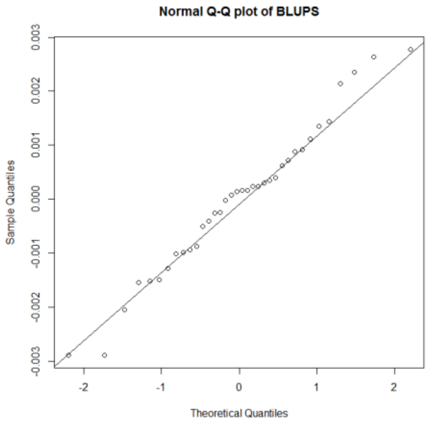

Bone Mineral Content: Top down modelling output

Hypothesis	Model1	Model 2	Test	Estimation method	Test statistic value	*p*-value
Is batch significant?	Batch	No batch	LRT	REML	$\chi^2(0:1)=16.76$	<0.0001
Is variance homogenous?	Homogenous	Heterogeneous	LRT	REML	$\chi^2(2)=0.157$	0.6911
Testing fixed effects – sex			Type 1 *F*-test	REML	F(1,359)=13.49	0.000
Testing fixed effect – genotype*sex			Type 1 *F*-test	REML	F(1,359)=0.987	0.324
Testing treatment - Is genotype significant?	With genotype	Without genotype	LRT	ML	$\chi^2(2)=69.85$	1.110223 e-16

Bone Mineral Content: Final model values and diagnostics

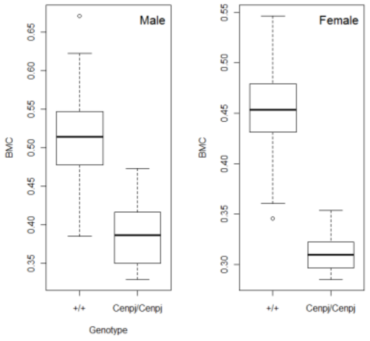

Parameter estimates:

	Value	Std.Error	DF	t-value	p-value
(Intercept)	0.45	0.00	360	111.39	0.0000
GenotypeCenpj/Cenpj	-0.13	0.01	360	-9.97	0.0000
GenderMale	0.06	0.00	360	13.90	0.0000

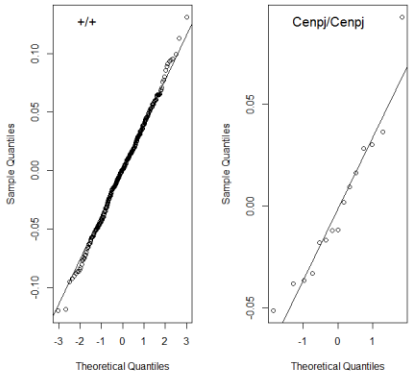

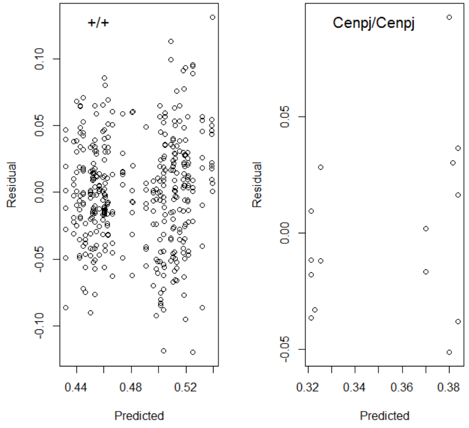

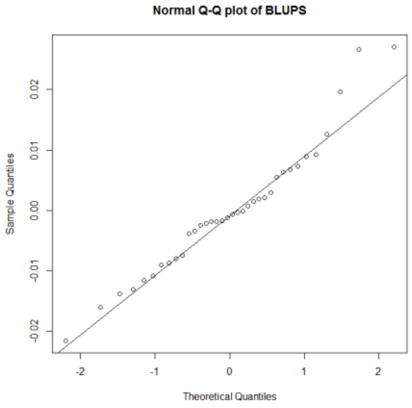

Lean Mass:
Top down modelling output

Hypothesis	Model1	Model 2	Test	Estimation method	Test statistic value	*p*-value
Is batch significant?	Batch	No batch	LRT	REML	$\chi^2(0:1)=20.85$	<0.0001
Is variance homogenous?	Homogenous	Heterogeneous	LRT	REML	$\chi^2(2)=5.40$	0.0201
Testing fixed effects – sex			Type 1 *F*-test	REML	F(1,359)=23.86	0.0000
Testing fixed effect – genotype*sex			Type 1 *F*-test	REML	F(1,359)=0.756	0.4496
Testing treatment - Is genotype significant?	With genotype	Without genotype	LRT	ML	$\chi^2(2)=51.97$	5.62e-13

Lean Mass: Final model values and diagnostics

Parameter estimates:

	Value	Std.Error	DF	t-value	p-value
(Intercept)	19.12	0.18	360	107.06	0.0000
GenotypeCenpj/Cenpj	-6.15	0.40	360	-15.33	0.0000
GenderMale	4.16	0.16	360	25.27	0.0000

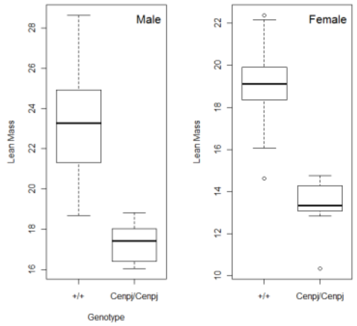

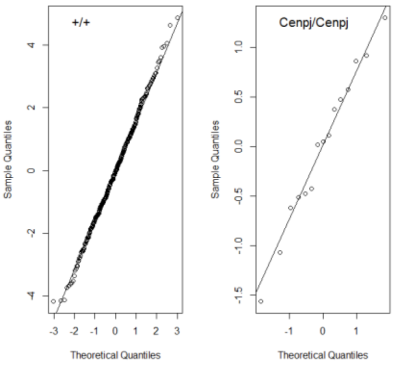

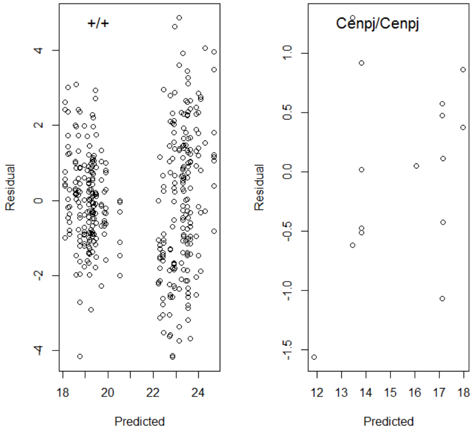

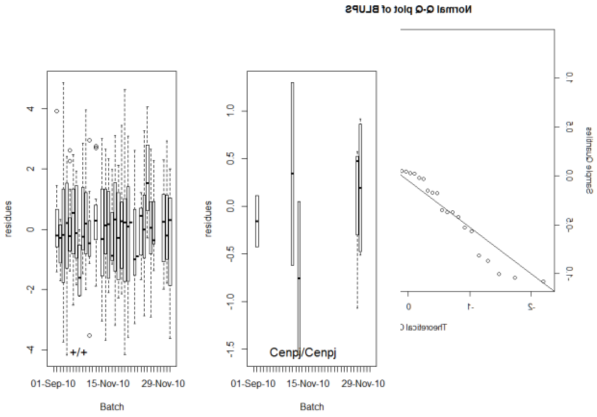

Fat Mass: Final model values and diagnostics

Hypothesis	Model1	Model 2	Test	Estimation method	Test statistic value	*p*-value
Is batch significant?	Batch	No batch	LRT	REML	$\chi^2(0:1)$= 33.87	<0.0001
Is variance homogenous?	Homogenous	Heterogeneous	LRT	REML	$\chi^2(2)$=0.305	0.5803
Testing fixed effects – sex			Type 1 F-test	REML	F(1,359)=7.087	0.0000
Testing fixed effect – genotype*sex			Type 1 F-test	REML	F(1,359)= 0.600	0.5487
Testing treatment - Is genotype significant?	With genotype	Without genotype	LRT	ML	$\chi^2(2)$=40.266	2.21e-10

Fat Mass: Final model values and diagnostics

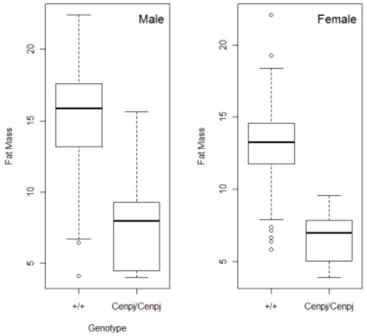

Parameter estimates:

	Value	Std.Error	DF	t-value	p-value
(Intercept)	13.27	0.31	360	42.87	0.0000
GenotypeCenpj/Cenpj	-6.88	0.94	360	-7.30	0.0000
GenderMale	2.03	0.28	360	7.31	0.0000

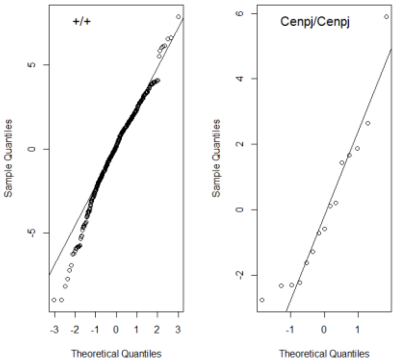

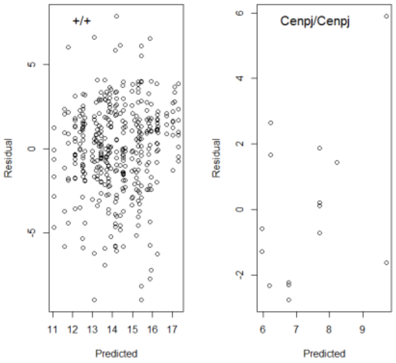

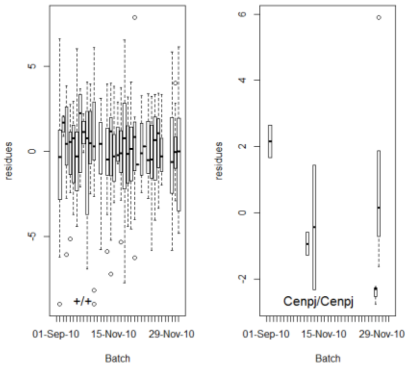

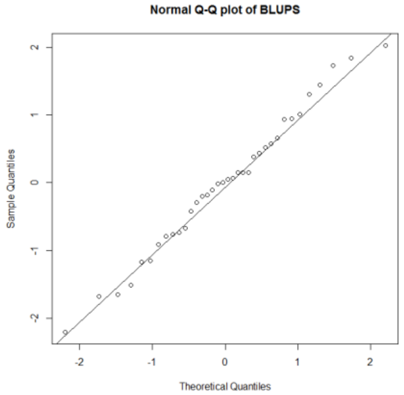

Dependent variable: Fat Percentage (Fat %)

Hypothesis	Model1	Model 2	Test	Estimation method	Test statistic value	*p*-value
Is batch significant?	Batch	No batch	LRT	REML	$\chi^2(0:1)=40.95$	<0.0001
Is variance homogenous?	Homogenous variance	Heterogeneous	LRT	REML	$\chi^2(2)=1.189$	0.2755
Testing fixed effects – sex			Type 1 F-test	REML	F(1,359)= -2.522	0.0121
Testing fixed effect – genotype*sex			Type 1 F-test	REML	F(1,359)=1.023	0.3068
Is genotype significant?	With genotype	Without genotype	LRT	ML	$\chi^2(2)= 16.81$	4.12e-05

Fat %: Final model values and diagnostics

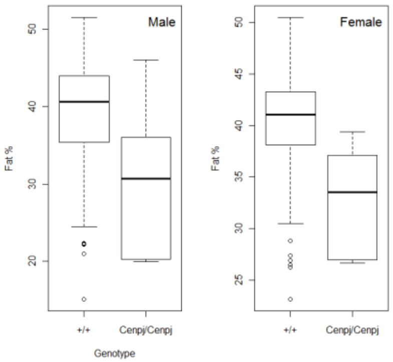

Parameter estimates:

	Value	Std.Error	DF	t-value	p-value
(Intercept)	40.53	0.62	360	65.42	0.0000
GenotypeCenpj/Cenpj	-8.15	1.83	360	-4.45	0.0000
GenderMale	-1.23	0.52	360	-2.38	0.0179

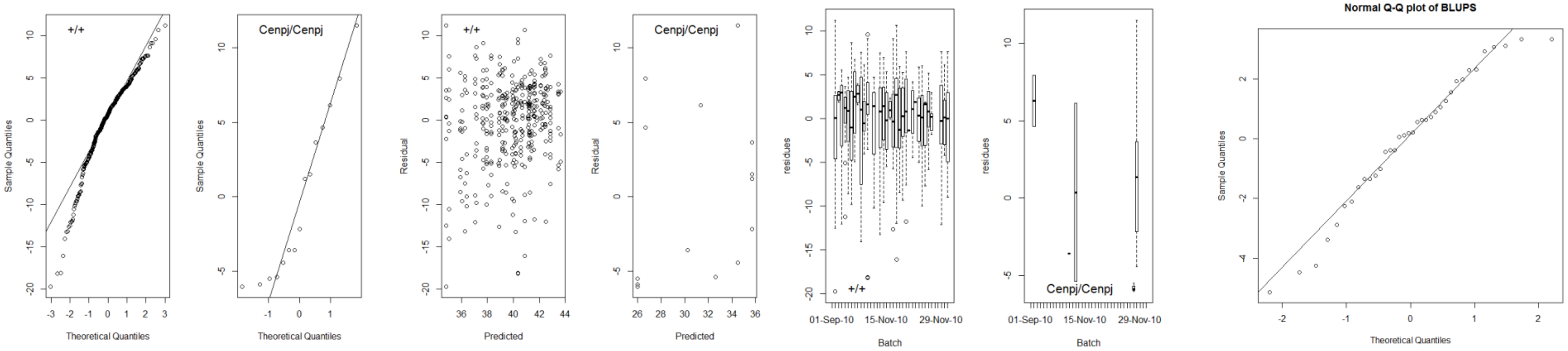

Summary

Variable	*p* value	Adjusted *p*' value	Genotype Estimate ± SE	Sex
Weight	0.0000000	0.0000	Y↓ -12.74±1.74	Y↑
Nose to tail length	0.0000000	0.0000	Y↓ -2.43±0.07	Y↑
BMD	0.0238	0.0386	N	N
BMC	1.11e-16	1.44E-15	Y↓ -0.13±0.01	Y↑
LM	5.62e-13	4.87E-12	Y↓ -6.15±0.40	Y↑
FM	2.21e-10	1.28E-09	Y↓ -6.88±0.94	Y↑
Fat %	4.12e-5	0.0001	Y↓ -8.15±1.83	Y↓

Y denotes a statistically significant effect and N indicates a non significant effect. The ↑ symbol indicates a positive estimated regression coefficient such that this effect leads to an increase in the dependent variable. Whilst, the ↓ symbol indicates a negative estimated regression coefficient such that this effect leads to a decrease in the dependent variable.

Mixed Model results 2

Starting model:

$Y_{ij} = \beta_o + \beta_1 Genotype1_{ij} + \beta_2 Sex1_{ij} + \beta_3 Weight1_{ij\,+}\, \beta_4 Genotype1_{ij} Sex1_{ij} + u_j + e_{ij}$.

Nose to tail length: Top down modelling output

Hypothesis	Model1	Model 2	Test	Estimation method	Test statistic value	*p*-value
Is batch significant?	Batch	No batch	LRT	REML	$\chi^2(0{:}1)=249.43$	<.0001
Is variance homogenous?	Homogenous	Heterogeneous	LRT	REML	$\chi^2(2)=2.48$	0.1151
Testing fixed effects – sex			Type 1 *F*-test	REML	F(1,366)=22.301	<.0001
Testing fixed effects – sex*genotype			Type 1 *F*-test	REML	F(1,366)=0.008	0.9278
Testing fixed effects – Weight			Type 1 *F*-test	REML	F(1,366)=194.36	<.0001
Testing treatment - Is genotype significant?	With genotype	Without genotype	LRT	ML	$\chi^2(2)=305.71$	0.0000

Nose to tail length: Final model values and diagnostics

Parameter estimates:

	Value	Std.Error	DF	t-value	p-value
(Intercept)	8.958876	0.085117	367	105.2532	0.0000
GenotypeCenpj/Cenpj	-2.01795	0.082112	367	-24.5756	0.0000
sexMale	0.10246	0.021583	367	4.74719	0.0000
Weight	0.0343	0.002452	367	13.98829	0.0000

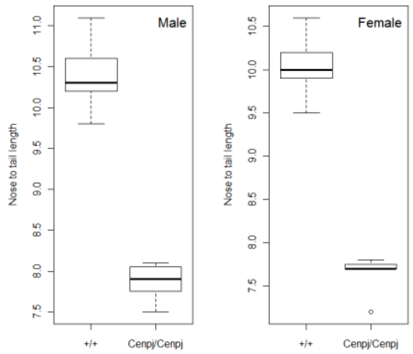

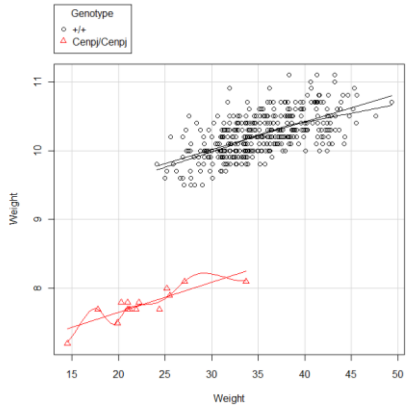

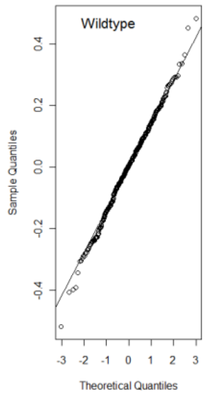

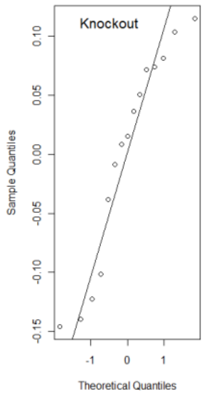

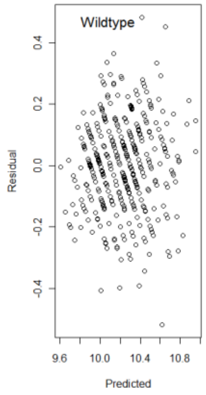

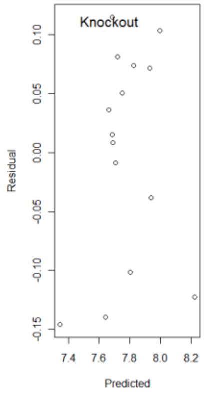

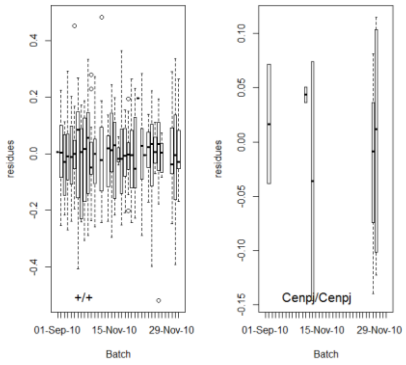

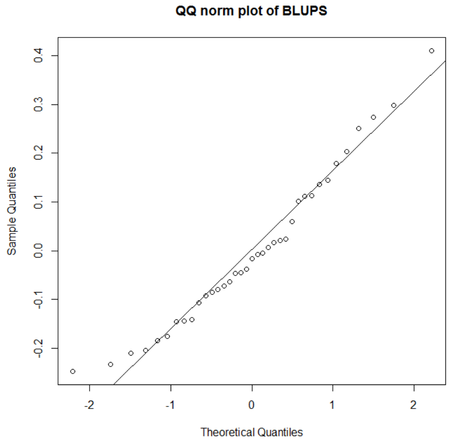

Bone mineral Density:
Top down modelling output

Hypothesis	Model1	Model 2	Test	Estimation method	Test statistic value	*p*-value
Is batch significant?	Batch	No batch	LRT	REML	$\chi^2(0:1)=84.71$	<.0001
Is variance homogenous?	Homogenous	Heterogeneous	LRT	REML	$\chi^2(2)=0.36$	0.5474
Testing fixed effects – sex			Type 1 F-test	REML	F(1,356)=2.27	0.1327
Testing fixed effects – weight			Type 1 F-test	REML	F(1,356)=40.868	<.0001
Testing fixed effect – genotype*sex			Type 1 F-test	REML	F(1,356)= 1.00	0.3167
Testing treatment - Is genotype significant?	With genotype	Without genotype	LRT	ML	$\chi^2(2)=1.4538$	0.2279

Bone Mineral Density: Final model values and diagnostics

Parameter estimates:

	Value	Std.Error	DF	t-value	p-value
(Intercept)	0.041149	0.00096	358	42.86411	0.0000
GenotypeCenpj/Cenpj	0.001182	0.000994	358	1.18914	0.2352
Weight	0.000268	2.54E-05	358	10.51291	0.0000

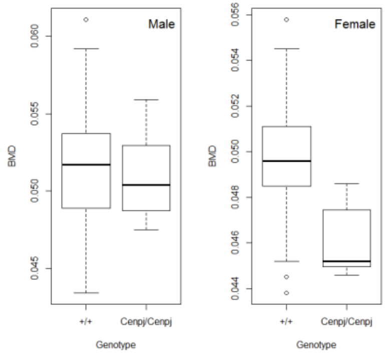

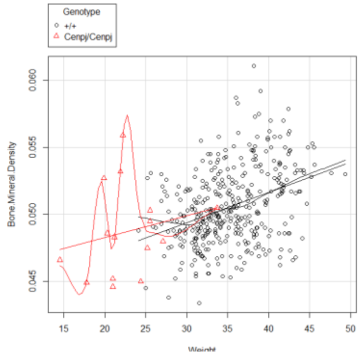

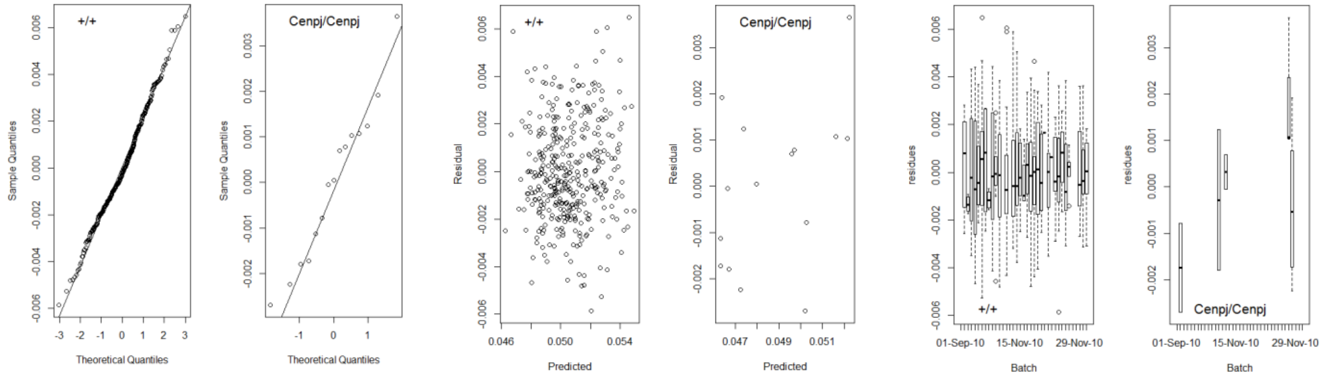

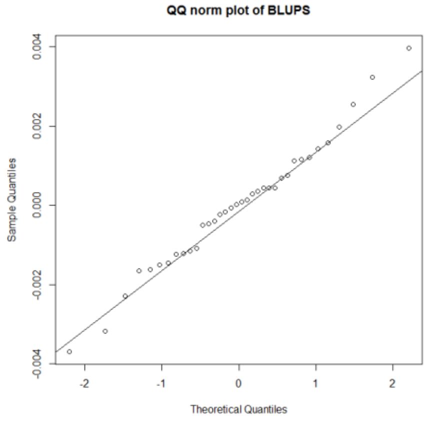

Bone Mineral Content: Top down modelling output

Hypothesis	Model1	Model 2	Test	Estimation method	Test statistic value	*p*-value
Is batch significant?	Batch	No batch	LRT	REML	$\chi^2(0:1)=28.30$	<.0001
Is variance homogenous?	Homogenous	Heterogeneous	LRT	REML	$\chi^2(2)=0.07$	0.7856
Testing fixed effects – sex			Type 1 *F*-test	REML	$F(1,356)=9.841$	0.0018
Testing fixed effects – weight			Type 1 *F*-test	REML	$F(1,356)=172.34$	<.0001
Testing fixed effect – genotype*sex			Type 1 *F*-test	REML	$F(1,356)=0.82$	0.3671
Testing treatment - Is genotype significant?	With genotype	Without genotype	LRT	ML	$\chi^2(2)=8.20$	0.00418

Bone Mineral Content: Final model values and diagnostics

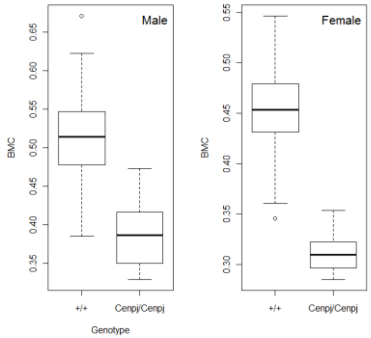

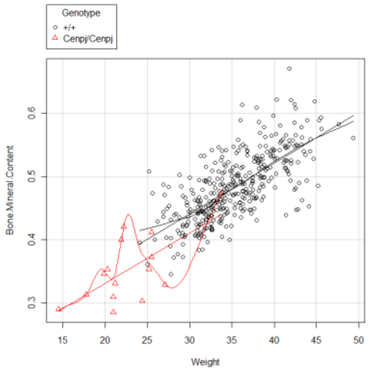

Parameter estimates:

	Value	Std.Error	DF	t-value	p-value
(Intercept)	0.223107	0.01791	357	12.45695	0.0000
GenotypeCenpj/Cenpj	-0.03914	0.013597	357	-2.87876	0.0042
sexMale	0.015644	0.0048	357	3.259352	0.0012
Weight	0.007131	0.000542	357	13.16408	0.0000

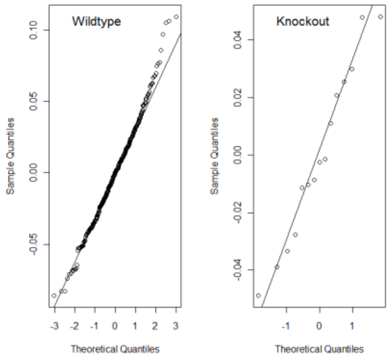

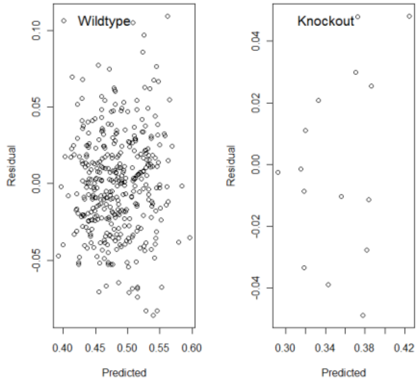

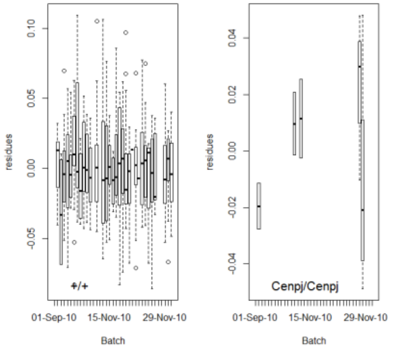

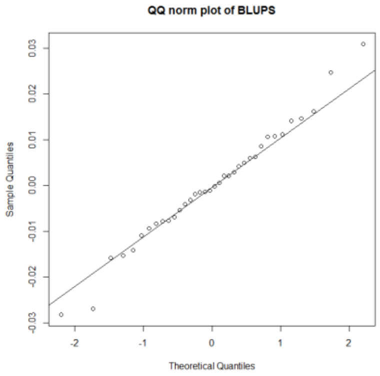

Lean Mass:
Top down modelling output

Hypothesis	Model1	Model 2	Test	Estimation method	Test statistic value	*p*-value
Is batch significant?	Batch	No batch	LRT	REML	$\chi^2(0:1)=41.67$	<.0001
Is variance homogenous?	Homogenous	Heterogeneous	LRT	REML	$\chi^2(2)=4.05$	0.0441
Testing fixed effects – sex			Type 1 *F*-test	REML	F(1,356)=151.46	<.0001
Testing fixed effects – weight			Type 1 *F*-test	REML	F(1,356)=196.22	<.0001
Testing fixed effect – genotype*sex			Type 1 *F*-test	REML	F(1,356)=0.1488	0.7027
Testing treatment - Is genotype significant?	With genotype	Without genotype	LRT	ML	$\chi^2(2)=21.52$	3.50e-06

Lean Mass: Final model values and diagnostics

Parameter estimates:

	Value	Std.Error	DF	t-value	p-value
(Intercept)	9.577604	0.698702	357	13.70772	0.0000
GenotypeCenpj/Cenpj	-2.4517	0.455325	357	-5.38451	0.0000
sexMale	2.333516	0.188149	357	12.40249	0.0000
Weight	0.296303	0.021111	357	14.03563	0.0000

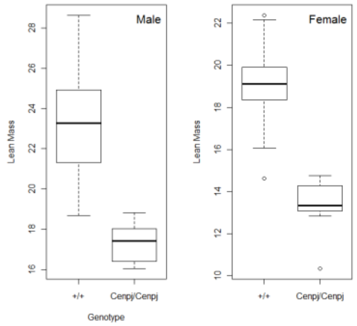

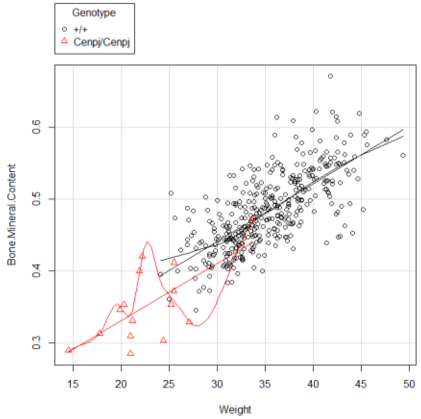

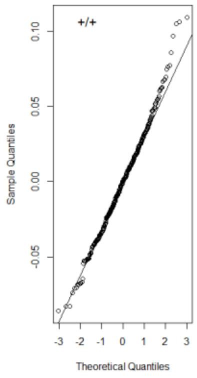

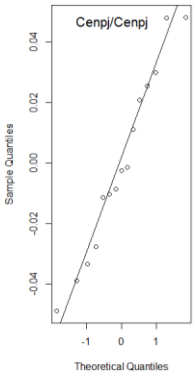

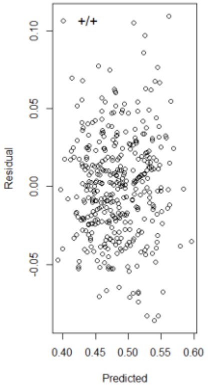

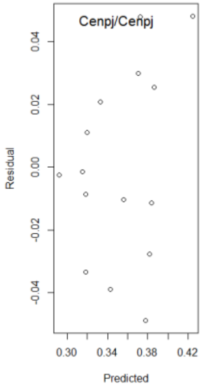

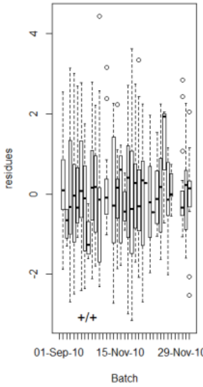

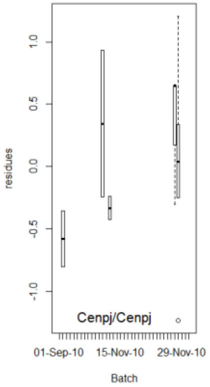

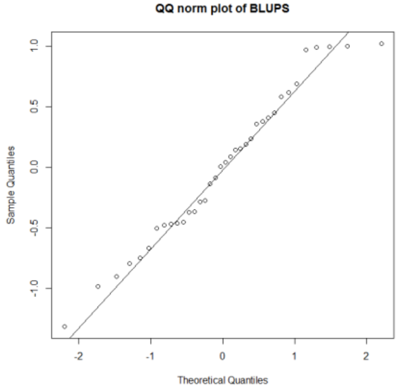

Fat Mass: Final model values and diagnostics

Hypothesis	Model1	Model 2	Test	Estimation method	Test statistic value	*p*-value
Is batch significant?	Batch	No batch	LRT	REML	$\chi^2(0{:}1)=197.9$	<.0001
Is variance homogenous?	Homogenous	Heterogeneous	LRT	REML	$\chi^2(2)=3.39$	0.0656
Testing fixed effects – sex			Type 1 F-test	REML	F(1,356)=144.88	<.0001
Testing fixed effects – weight			Type 1 F-test	REML	F(1,356)=1055.95	<.0001
Testing fixed effect – genotype*sex			Type 1 F-test	REML	F(1,356)= 0.0551	0.8145
Testing treatment - Is genotype significant?	With genotype	Without genotype	LRT	ML	$\chi^2(2)=19.82$	8.49e-06

Fat Mass: Final model values and diagnostics

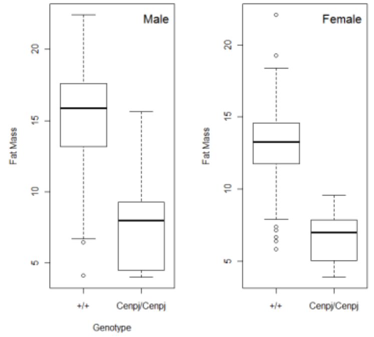

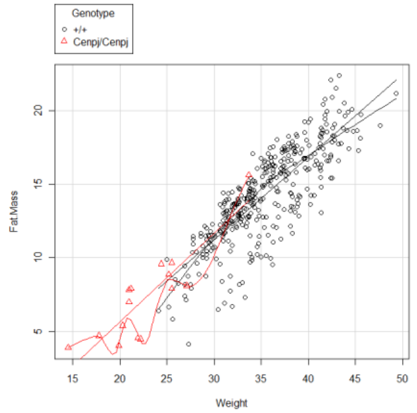

Parameter estimates:

	Value	Std.Error	DF	t-value	p-value
(Intercept)	-10.5064	0.745039	357	-14.1019	0.0000
GenotypeCenpj/Cenpj	2.480324	0.549868	357	4.51076	0.0000
sexMale	-2.43322	0.200874	357	-12.1132	0.0000
Weight	0.735736	0.022607	357	32.5454	0.0000

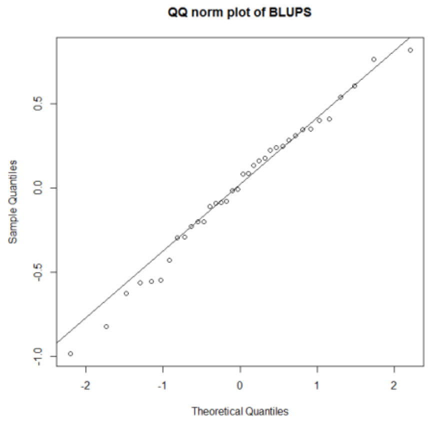

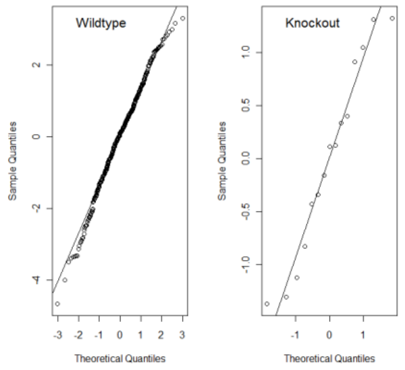

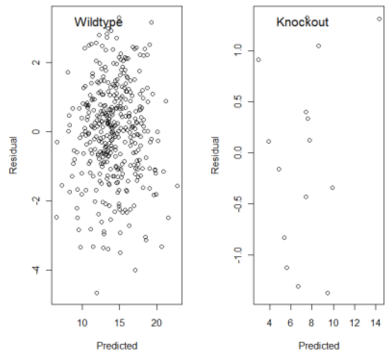

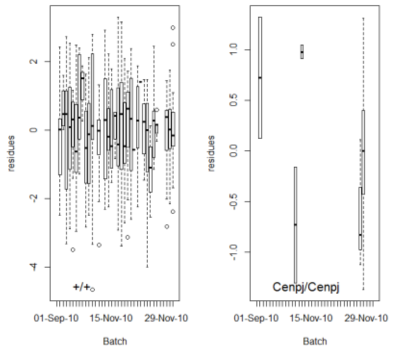

Dependent variable: Fat Percentage (Fat %)

Hypothesis	Model1	Model 2	Test	Estimation method	Test statistic value	*p*-value
Is batch significant?	Batch	No batch	LRT	REML	$\chi^2(0:1)=27.35$	<.0001
Is variance homogenous?	Homogenous variance	Heterogeneous	LRT	REML	$\chi^2(2)=0.02$	0.8846
Testing fixed effects – sex			Type 1 F-test	REML	F(1,356)= 161.95	<.0001
Testing fixed effects – weight			Type 1 F-test	REML	F(1,356)= 237.43	<.0001
Testing fixed effect – genotype*sex			Type 1 F-test	REML	F(1,356)=0.403	0.5259
Is genotype significant?	With genotype	Without genotype	LRT	ML	$\chi^2(2)= 7.17$	0.0074

Fat %: Final model values and diagnostics

Parameter estimates:

	Value	Std.Error	DF	t-value	p-value
(Intercept)	8.645307	2.117703	357	4.082399	0.0001
GenotypeCenpj/Cenpj	4.288679	1.60676	357	2.669148	0.0080
sexMale	-7.2357	0.56758	357	-12.7483	0.0000
Weight	0.987632	0.064056	357	15.4182	0.0000

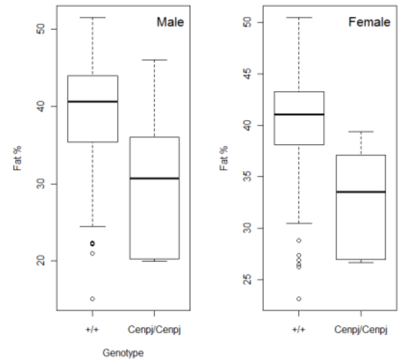

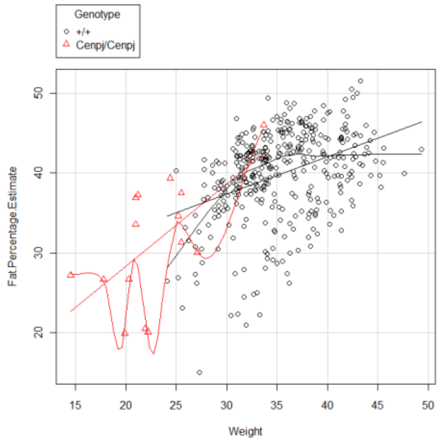

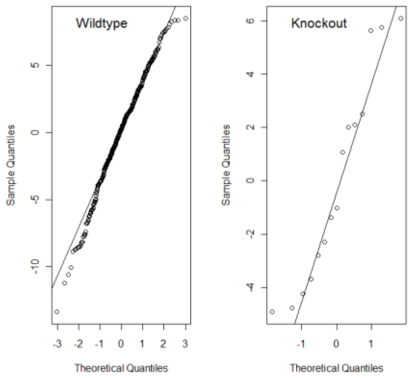

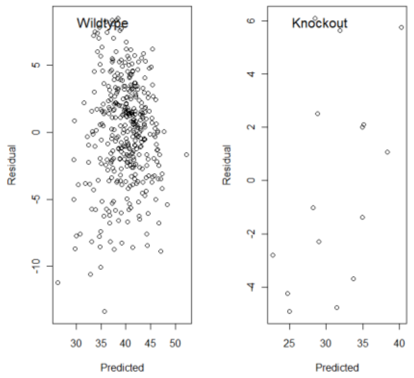

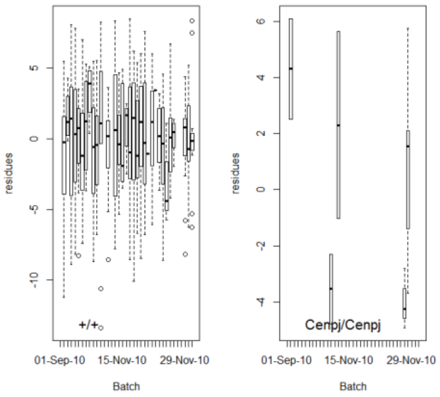

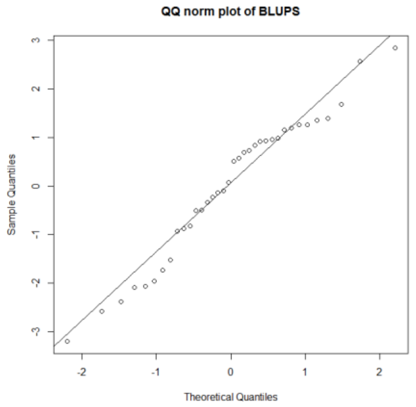

Summary

Variable	*p* value	Adjusted *p*' value	Genotype Estimate ± SE	Sex	Weight
Nose to tail length	0.000000	0.000	Y↓ -2.02±0.08	Y↑	Y↑
BMD	0.2279	0.2693	N	N	Y↑
BMC	0.0042	0.0075	Y↓ -0.039±0.01	Y↑	Y↑
LM	3.5e-6	1.30E-05	Y↓ -2.45±0.45	Y↑	Y↑
FM	8.49e-6	2.76E-05	Y↑ 2.48±0.55	Y↓	Y↑
Fat %	0.0074	0.0128	Y↑ 4.28±1.68	Y↓	Y↑

Y denotes a statistically significant effect and N indicates a non significant effect. The ↑ symbol indicates a positive estimated regression coefficient such that this effect leads to an increase in the dependent variable. Whilst, the ↓ symbol indicates a negative estimated regression coefficient such that this effect leads to a decrease in the dependent variable.
