# Supplementary material for: Robust and Sensitive Analysis of Mouse Knockout Phenotypes
Source: PLoS One. 2012 Dec 26;7(12):e52410. doi: 10.1371/journal.pone.0052410 (PMC3530558; doi:10.1371/journal.pone.0052410)
Supplement: File S5 — Detailed mixed model output for the allele Sparctm1a(EUCOMM)Wtsi and associated DEXA data. Legend: For each trait studied, for each model fitting procedures, the final model output was captured and the data visualised with a boxplot. Furthermore, to test the quality of model fit, a number of graphical diagnostic plots were generated for each gene and trait. (PDF) [file pone.0052410.s008.pdf]

# *Sparc*$^{tm1a(EUCOMM)Wtsi}$

## DEXA mixed model analysis

| Variable | Unit of analysis |
|---|---|
| Weight | g |
| Nose to tail length | cm |
| Bone mineral density | $g/cm^2$ |
| Bone mineral content | g |
| Lean mass | g |
| Fat mass | g |
| Fat percentage | % |

Abbreviations:
LRT: Likelihood ratio test
ML: Maximum likelihood
REML: Residual maximum likelihood
BMC: Bone mineral content
BMD: Bone mineral density
LM: Lean mass
FM: Fat mass
Fat %: Fat percentage
SE: Standard Error

# Information on diagnostic graphs presented for each model

A: A boxplot comparison of the dependent variable for each genotype for each sex.

B: Weight versus dependent variable scatterplot For each genotype a regression and a Loess line is fitted. A Loess line is a locally weighted linear line. Graph only relevant for models that include weight as a fixed effect.

C: Normal Q-Q plot of the best linear unbiased prediction of random effects (BLUPS).

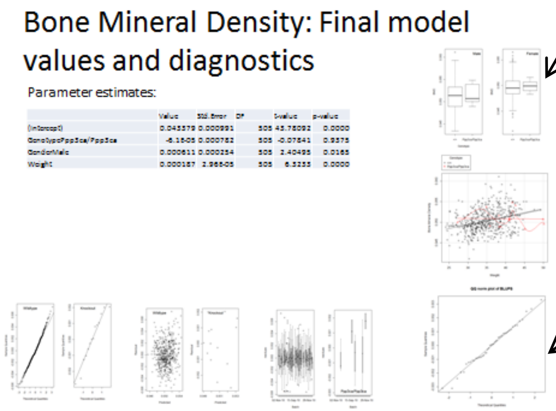


D: For each genotype group, conditional raw residues are plotted versus batch.

E: For each genotype group, a plot of conditional raw residue versus predicted values.

F: Normal Q-Q plots of conditional raw residues for each genotype.

# Mixed Model results 1

Starting model:

$Y_{ij} = \beta_o + \beta_1 Genotype1_{ij} + \beta_2 Sex1_{ij} + \beta_3 Genotype1_{ij} Sex1_{ij} + u_j + e_{ij}$.

# Weight: Top down modelling output

| Hypothesis | Model1 | Model 2 | Test | Estimation method | Test statistic value | *p*-value |
|---|---|---|---|---|---|---|
| Is batch significant? | Batch | No batch | LRT | REML | $\chi^2(0:1)=11.17$ | 8e-4 |
| Is variance homogenous? | Homogenous | Heterogeneous | LRT | REML | $\chi^2(2)=1.43$ | 0.2311 |
| Testing fixed effects – sex | | | Type 1 *F*-test | REML | F(1,417)=23.400 | 0.0000 |
| Testing fixed effects – sex*genotype | | | Type 1 *F*-test | REML | F(1,47)=0.9351 | 0.3545 |
| Testing treatment - Is genotype significant? | With genotype | Without genotype | LRT | ML | $\chi^2(2)=16.97$ | 3.792e-05 |

# Weight: Final model values and diagnostics

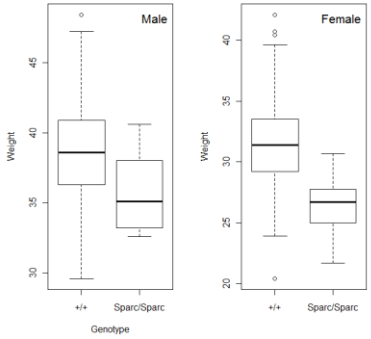


Parameter estimates:

| | Value | Std.Error | DF | t-value | p-value |
|---|---|---|---|---|---|
| (Intercept) | 31.554 | 0.296 | 417 | 106.534 | 0.0000 |
| GenotypeSparc/Sparc | -4.082 | 0.979 | 417 | -4.168 | 0.0000 |
| GenderMale | 7.430 | 0.311 | 417 | 23.888 | 0.0000 |

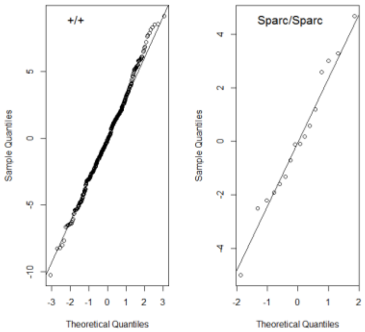


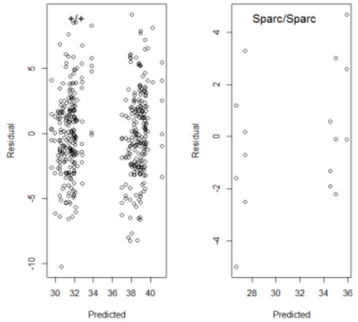


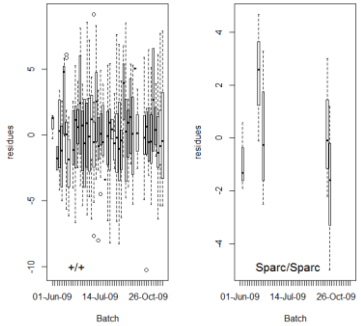


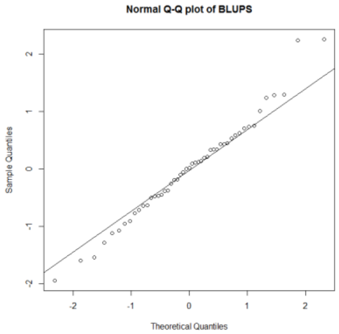

# Nose to tail length: Top down modelling output

| Hypothesis | Model1 | Model 2 | Test | Estimation method | Test statistic value | *p*-value |
|---|---|---|---|---|---|---|
| Is batch significant? | Batch | No batch | LRT | REML | $\chi^2(0:1)=91.568$ | <.0001 |
| Is variance homogenous? | Homogenous | Heterogeneous | LRT | REML | $\chi^2(2)=01.372$ | 0.2414 |
| Testing fixed effects – sex | | | Type 1 *F*-test | REML | F(1,417)=-4.9781 | 0.0000 |
| Testing fixed effects – sex*genotype | | | Type 1 *F*-test | REML | F(1,47)=-0.0900 | 0.9287 |
| Testing treatment - Is genotype significant? | With genotype | Without genotype | LRT | ML | $\chi^2(2)=41.667$ | 1.081e-10 |

# Nose to tail length: Final model values and diagnostics

Parameter estimates:

| | Value | Std.Error | DF | t-value | p-value |
|---|---|---|---|---|---|
| (Intercept) | 10.036 | 0.026 | 417 | 390.738 | 0.0000 |
| GenotypeSparc/Sparc | -0.491 | 0.073 | 417 | -6.728 | 0.0000 |
| GenderMale | 0.373 | 0.020 | 417 | 18.965 | 0.0000 |

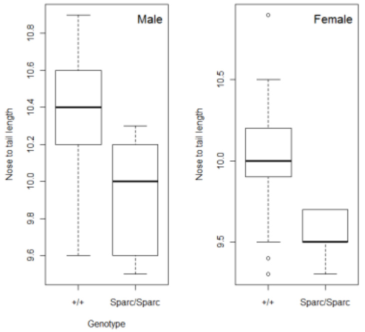


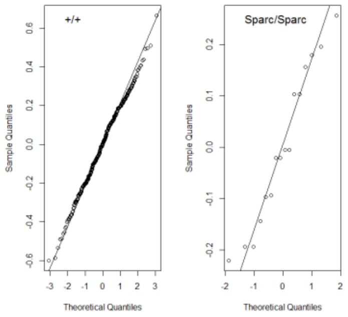


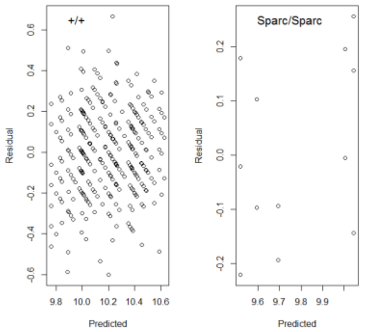


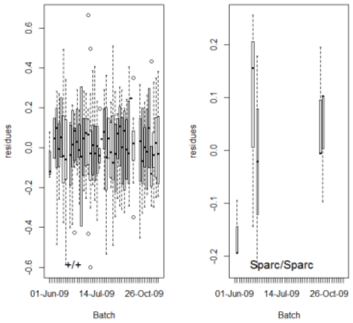


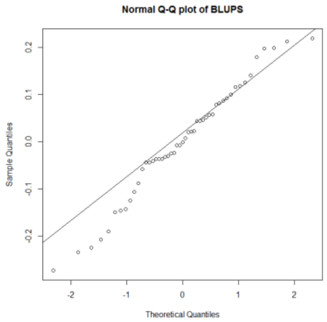

# Bone mineral Density: Top down modelling output

| Hypothesis | Model1 | Model 2 | Test | Estimation method | Test statistic value | *p*-value |
|---|---|---|---|---|---|---|
| Is batch significant? | Batch | No batch | LRT | REML | $\chi^2(0:1)=33.815$ | <0.0001 |
| Is variance homogenous? | Homogenous | Heterogeneous | LRT | REML | $\chi^2(2)=0.7423$ | 0.3889 |
| Testing fixed effects – sex | | | Type 1 F-test | REML | F(1,412)=8.744 | 0.0000 |
| Testing fixed effect – genotype*sex | | | Type 1 F-test | REML | F(1,47)=-0.129 | 0.8978 |
| Testing treatment - Is genotype significant? | With genotype | Without genotype | LRT | ML | $\chi^2(2)=26.479$ | 2.66e-7 |

# Bone Mineral Density: Final model values and diagnostics

Parameter estimates:

| | Value | Std.Error | DF | t-value | p-value |
|---|---|---|---|---|---|
| (Intercept) | 0.049 | 0.000 | 412 | 217.665 | 0.0000 |
| GenotypeSparc/Sparc | -0.004 | 0.001 | 412 | -5.377 | 0.0000 |
| GenderMale | 0.002 | 0.000 | 412 | 8.831 | 0.0000 |

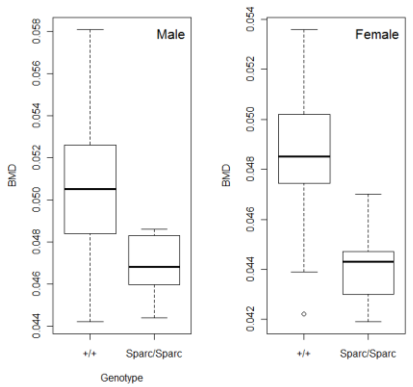


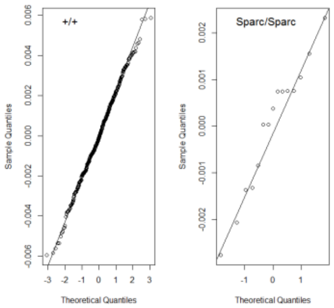


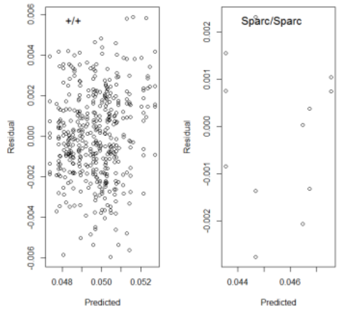


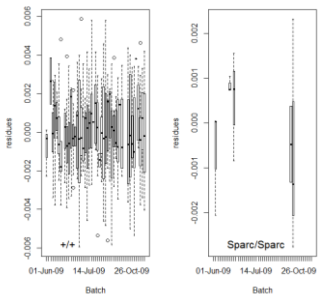


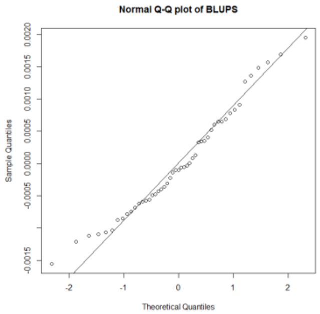

# Bone Mineral Content: Top down modelling output

| Hypothesis | Model1 | Model 2 | Test | Estimation method | Test statistic value | *p*-value |
|---|---|---|---|---|---|---|
| Is batch significant? | Batch | No batch | LRT | REML | $\chi^2(0:1)=3.49327$ | 0.0616 |
| Is variance homogenous? | Homogenous | Heterogeneous | LRT | REML | $\chi^2(2)=0.742$ | 0.3889 |
| Testing fixed effects – sex | | | Type 1 *F*-test | REML | F(1,412)=15.82 | 0.0000 |
| Testing fixed effect – genotype*sex | | | Type 1 *F*-test | REML | F(1,47)=0.8496 | 0.3998 |
| Testing treatment - Is genotype significant? | With genotype | Without genotype | LRT | ML | $\chi^2(2)=21.760$ | 3.089e-6 |

# Bone Mineral Content: Final model values and diagnostics

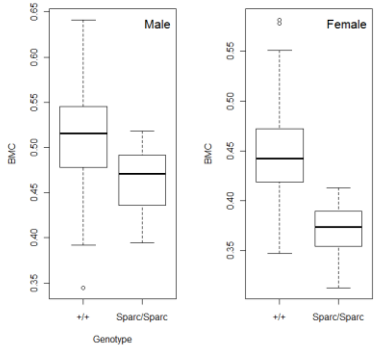


Parameter estimates:

| | Value | Std.Error | DF | t-value | p-value |
|---|---|---|---|---|---|
| (Intercept) | 0.446 | 0.003 | 412 | 130.326 | 0.0000 |
| GenotypeSparc/Sparc | -0.060 | 0.013 | 412 | -4.759 | 0.0000 |
| GenderMale | 0.068 | 0.004 | 412 | 16.222 | 0.0000 |

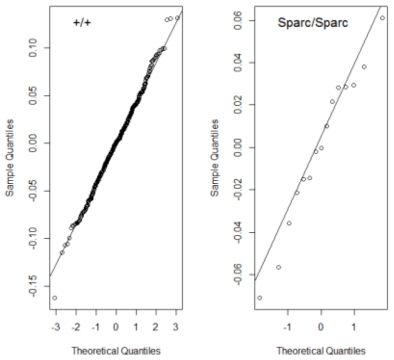


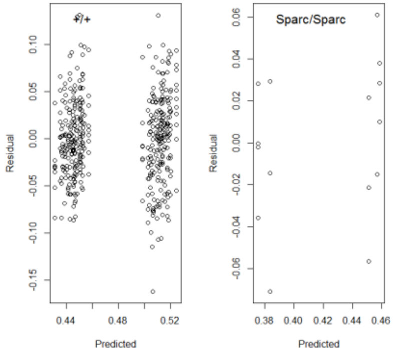


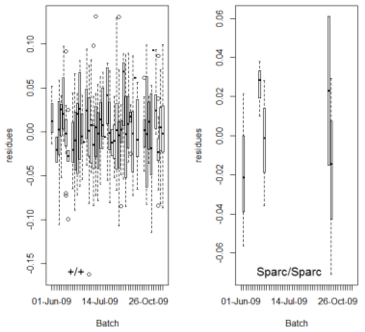


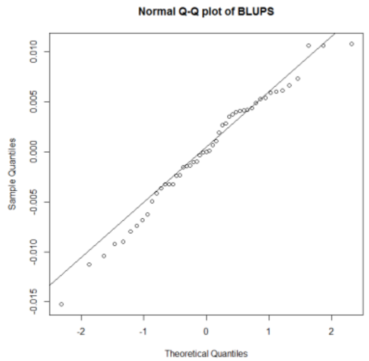

# Lean Mass: Top down modelling output

| Hypothesis | Model1 | Model 2 | Test | Estimation method | Test statistic value | *p*-value |
|---|---|---|---|---|---|---|
| Is batch significant? | Batch | No batch | LRT | REML | $\chi^2(0:1)=31.43$ | <.0001 |
| Is variance homogenous? | Homogenous | Heterogeneous | LRT | REML | $\chi^2(2)=4.052$ | 0.0441 |
| Testing fixed effects – sex | | | Type 1 *F*-test | REML | F(1,412)=25.95 | 0.0000 |
| Testing fixed effect – genotype*sex | | | Type 1 *F*-test | REML | F(1,47)=-0.480 | 0.6331 |
| Testing treatment - Is genotype significant? | With genotype | Without genotype | LRT | ML | $\chi^2(2)=11.556$ | 0.000675 |

# Lean Mass: Final model values and diagnostics

Parameter estimates:

| | Value | Std.Error | DF | t-value | p-value |
|---|---|---|---|---|---|
| (Intercept) | 18.441 | 0.170 | 412 | 108.679 | 0.0000 |
| GenotypeSparc/Sparc | -1.862 | 0.542 | 412 | -3.436 | 0.0006 |
| GenderMale | 4.210 | 0.161 | 412 | 26.190 | 0.0000 |

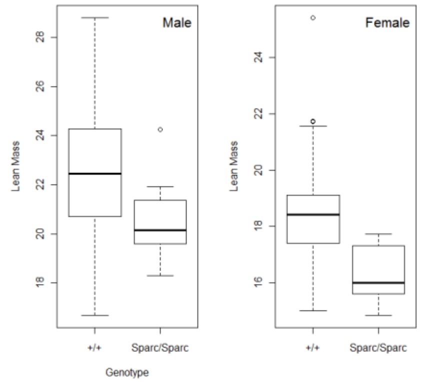


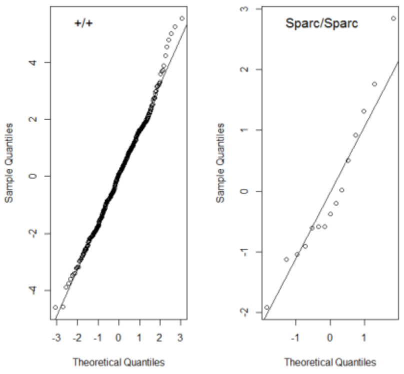


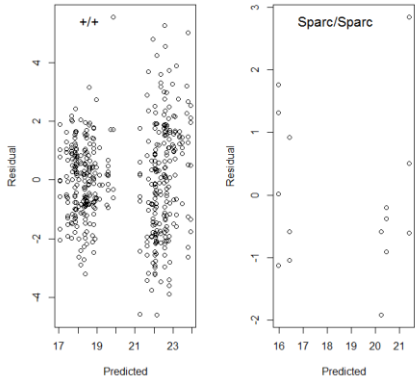


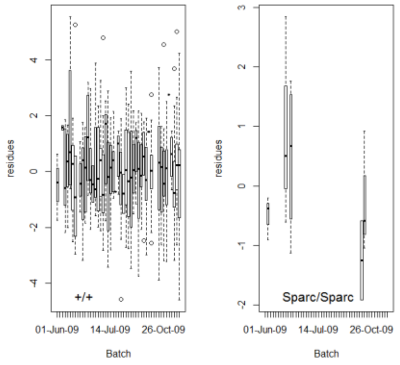


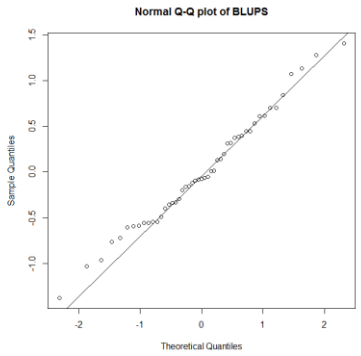

# Fat Mass: Final model values and diagnostics

| Hypothesis | Model1 | Model 2 | Test | Estimation method | Test statistic value | *p*-value |
|---|---|---|---|---|---|---|
| Is batch significant? | Batch | No batch | LRT | REML | $\chi^2(0:1)=28.715$ | <.0001 |
| Is variance homogenous? | Homogenous | Heterogeneous | LRT | REML | $\chi^2(2)=4.0524$ | 0.0441 |
| Testing fixed effects – sex | | | Type 1 F-test | REML | F(1,412)=13.048 | 0.0000 |
| Testing fixed effect – genotype*sex | | | Type 1 F-test | REML | F(1,47)=1.0322 | 0.3072 |
| Testing treatment - Is genotype significant? | With genotype | Without genotype | LRT | ML | $\chi^2(2)=13.586$ | 0.00022 77 |

# Fat Mass: Final model values and diagnostics

Parameter estimates:

| | Value | Std.Error | DF | t-value | p-value |
|---|---|---|---|---|---|
| (Intercept) | 13.411 | 0.273 | 412 | 49.122 | 0.0000 |
| GenotypeSparc/Sparc | -2.583 | 0.649 | 412 | -3.979 | 0.0001 |
| GenderMale | 3.419 | 0.252 | 412 | 13.541 | 0.0000 |

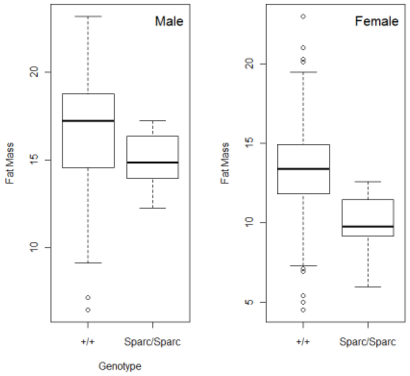


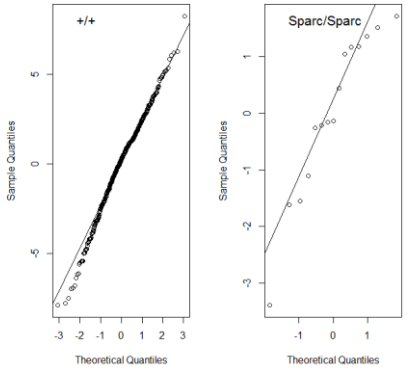


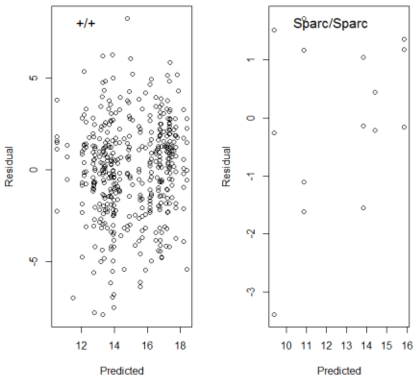


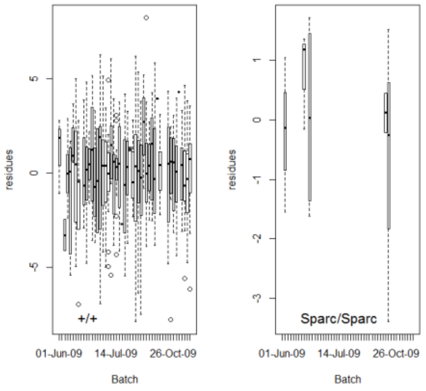


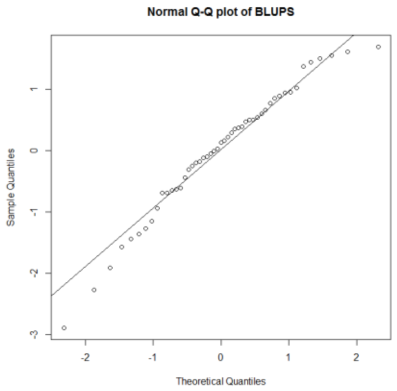

# Dependent variable: Fat Percentage (Fat %)

| Hypothesis | Model1 | Model 2 | Test | Estimation method | Test statistic value | *p*-value |
|---|---|---|---|---|---|---|
| Is batch significant? | Batch | No batch | LRT | REML | $\chi^2(0:1)=42.334$ | <.0001 |
| Is variance homogenous? | Homogenous variance | Heterogeneous | LRT | REML | $\chi^2(2)=2.6767$ | 0.1018 |
| Testing fixed effects – sex | | | Type 1 F-test | REML | $F(1,411)=1.371$ | 0.1711 |
| Testing fixed effect – genotype*sex | | | Type 1 F-test | REML | $F(1,47)=1.418$ | 0.1627 |
| Is genotype significant? | With genotype | Without genotype | LRT | ML | $\chi^2(2)=2.5998$ | 0.10687 |

# Fat %: Final model values and diagnostics

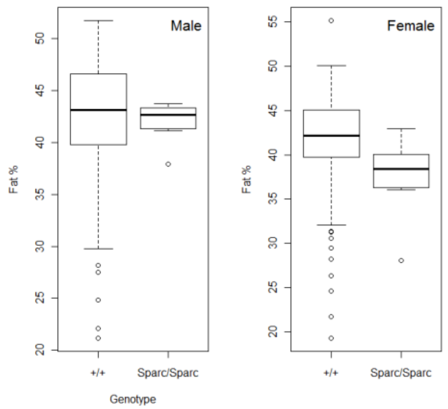


Parameter estimates:

| | Value | Std.Error | DF | t-value | p-value |
|---|---|---|---|---|---|
| (Intercept) | 42.036 | 0.430 | 413 | 97.850 | 0.0000 |
| GenotypeSparc/Sparc | -2.443 | 1.519 | 413 | -1.608 | 0.1086 |

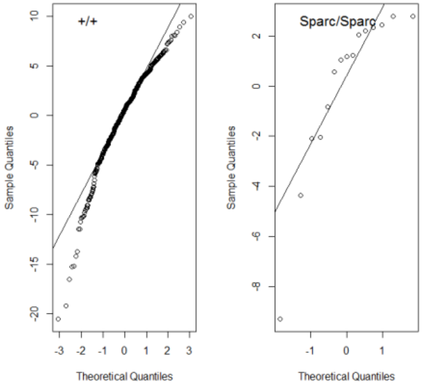


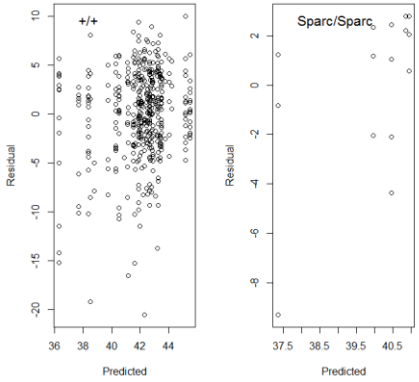


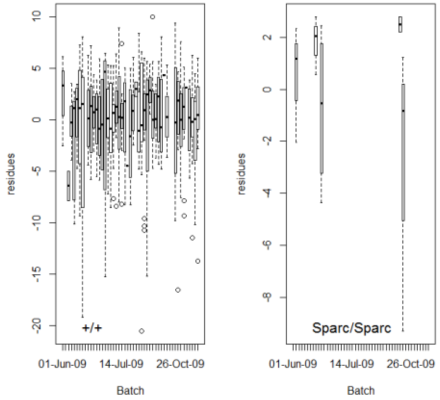


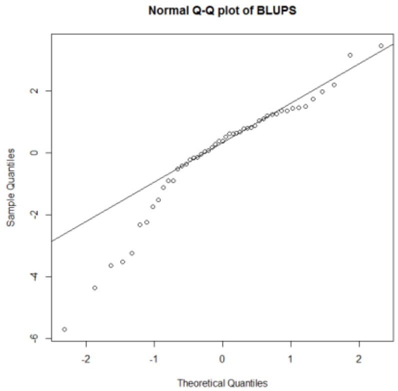

# Summary

| Variable | *p* value | Adjusted *p*' value | Genotype Estimate ± SE | Sex |
|---|---|---|---|---|
| Weight | 1.58e-7 | 7.52E-07 | Y↓ -4.08±0.979 | Y↑ |
| Nose to tail length | 1.08e-10 | 7.02E-10 | Y↓ -0.491±0.073 | Y↑ |
| BMD | 2.66e-7 | 1.15E-06 | Y↓ -0.004±0.001 | N |
| BMC | 3.09e-6 | 1.24E-05 | Y↓ -0.060±0.0013 | Y↑ |
| LM | 0.0006 | 0.0017 | Y↓ -1.862±0.542 | Y↑ |
| FM | 0.0002 | 0.0006 | Y↓ -2.583±0.649 | Y↓ |
| Fat % | 0.1068 | 0.1501 | N | Y↓ |

Y denotes a statistically significant effect and N indicates a non significant effect. The ↑ symbol indicates a positive estimated regression coefficient such that this effect leads to an increase in the dependent variable. Whilst, the ↓ symbol indicates a negative estimated regression coefficient such that this effect leads to a decrease in the dependent variable.

# Mixed Model results 2

Starting model:

$$Y_{ij} = \beta_o + \beta_1 Genotype1_{ij} + \beta_2 Sex1_{ij} + \beta_3 Weight1_{ij\,+}\, \beta_4 Genotype1_{ij} Sex1_{ij} + u_j + e_{ij}.$$

# Nose to tail length: Top down modelling output

| Hypothesis | Model1 | Model 2 | Test | Estimation method | Test statistic value | *p*-value |
|---|---|---|---|---|---|---|
| Is batch significant? | Batch | No batch | LRT | REML | $\chi^2(0:1)=170.49$ | <0.0001 |
| Is variance homogenous? | Homogenous | Heterogeneous | LRT | REML | $\chi^2(2)=0.007$ | 0.9319 |
| Testing fixed effects – sex | | | Type 1 *F*-test | REML | F(1,416)=7.981 | 0.0050 |
| Testing fixed effects – sex*genotype | | | Type 1 *F*-test | REML | F(1,47)=0.219 | 0.6418 |
| Testing fixed effects – Weight | | | Type 1 *F*-test | REML | F(1,416)=340.48 | <0.0001 |
| Testing treatment - Is genotype significant? | With genotype | Without genotype | LRT | ML | $\chi^2(2)=27.48$ | 1.587e-07 |

# Nose to tail length: Final model values and diagnostics

Parameter estimates:

| | Value | Std.Error | DF | t-value | p-value |
|---|---|---|---|---|---|
| (Intercept) | 8.728943 | 0.074709 | 416 | 116.839 | 0.0000 |
| GenotypeSparc/Sparc | -0.32514 | 0.060934 | 416 | -5.33603 | 0.0000 |
| sexMale | 0.062747 | 0.022384 | 416 | 2.80322 | 0.0053 |
| Weight | 0.041395 | 0.002243 | 416 | 18.45883 | 0.0000 |

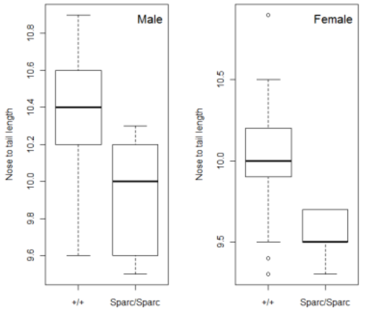


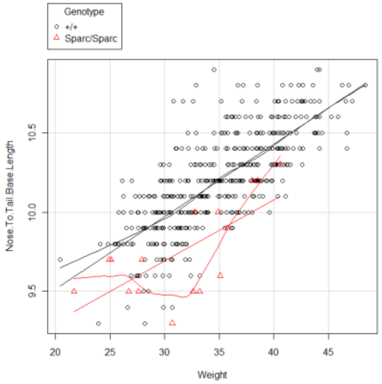


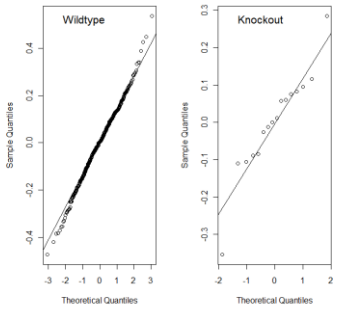


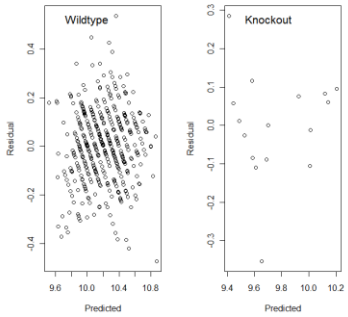


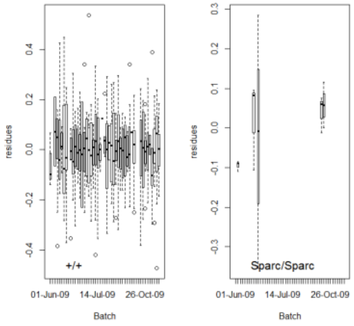


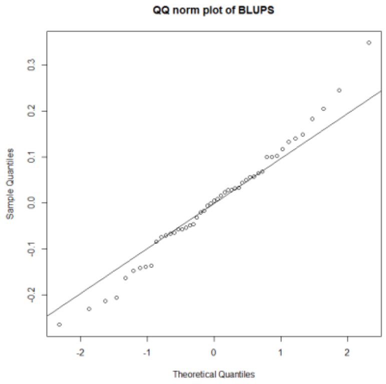

# Bone mineral Density:
# Top down modelling output

| Hypothesis | Model1 | Model 2 | Test | Estimation method | Test statistic value | *p*-value |
|---|---|---|---|---|---|---|
| Is batch significant? | Batch | No batch | LRT | REML | $\chi^2(0:1)=50.79$ | <0.0001 |
| Is variance homogenous? | Homogenous | Heterogeneous | LRT | REML | $\chi^2(2)=5.10$ | 0.0239 |
| Testing fixed effects – sex | | | Type 1 F-test | REML | F(1,411)=0.257 | 0.6122 |
| Testing fixed effects – weight | | | Type 1 F-test | REML | F(1,411)=88.00 | <0.0001 |
| Testing fixed effect – genotype*sex | | | Type 1 F-test | REML | F(1,47)=0.47 | 0.4947 |
| Testing treatment - Is genotype significant? | With genotype | Without genotype | LRT | ML | $\chi^2(2)=20.180$ | 7.04e-06 |

# Bone Mineral Density: Final model values and diagnostics

Parameter estimates:

| | Value | Std.Error | DF | t-value | p-value |
|---|---|---|---|---|---|
| (Intercept) | 0.040777 | 0.000706 | 412 | 57.72091 | 0.0000 |
| GenotypeSparc/Sparc | -0.00259 | 0.000513 | 412 | -5.04827 | 0.0000 |
| Weight | 0.000257 | 1.92E-05 | 412 | 13.39569 | 0.0000 |

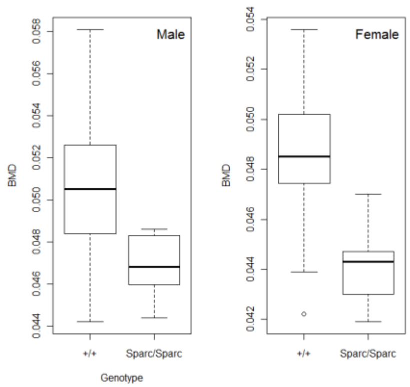


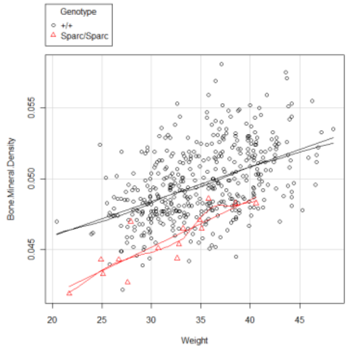


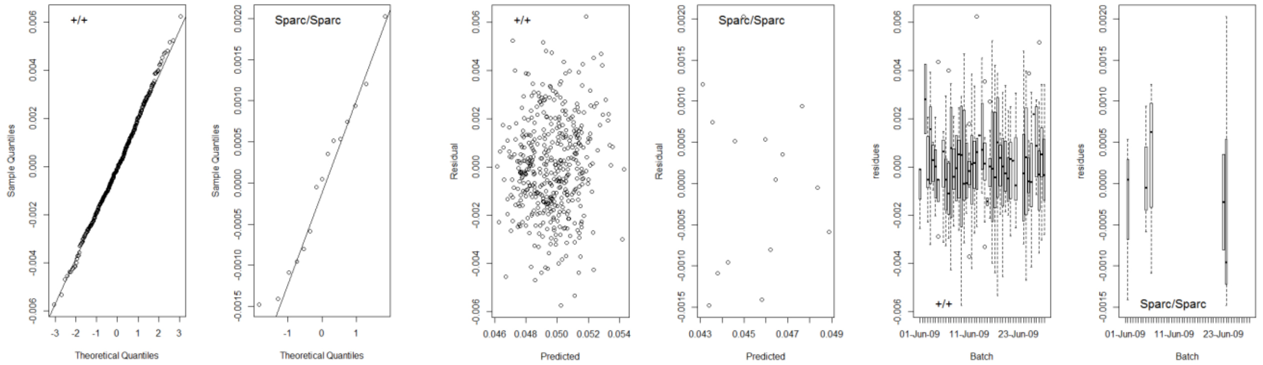


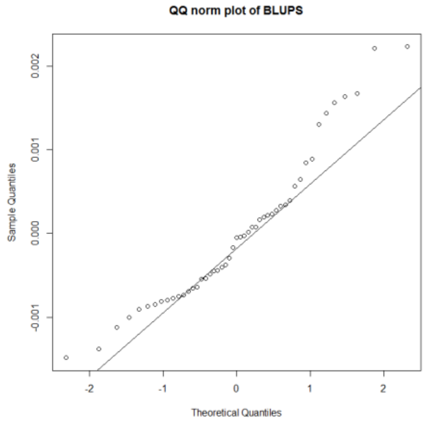

# Bone Mineral Content: Top down modelling output

| Hypothesis | Model1 | Model 2 | Test | Estimation method | Test statistic value | *p*-value |
|---|---|---|---|---|---|---|
| Is batch significant? | Batch | No batch | LRT | REML | $\chi^2(0:1)=8.26$ | 0.004 |
| Is variance homogenous? | Homogenous | Heterogeneous | LRT | REML | $\chi^2(2)=0.951$ | 0.3292 |
| Testing fixed effects – sex | | | Type 1 *F*-test | REML | F(1,411)=2.072 | 0.1507 |
| Testing fixed effects – weight | | | Type 1 *F*-test | REML | F(1,411)=285.8 | <0.0001 |
| Testing fixed effect – genotype*sex | | | Type 1 *F*-test | REML | F(1,47)=0.087 | 0.7688 |
| Testing treatment - Is genotype significant? | With genotype | Without genotype | LRT | ML | $\chi^2(2)=4.709$ | 0.03 |

# Bone Mineral Content: Final model values and diagnostics

Parameter estimates:

| | Value | Std.Error | DF | t-value | p-value |
|---|---|---|---|---|---|
| (Intercept) | 0.19 | 0.02 | 411 | 12.16 | 0.0000 |
| GenotypeSparc/Sparc | -0.02 | 0.01 | 411 | -2.39 | 0.0175 |
| Weight | 0.01 | 0.00 | 411 | 16.95 | 0.0000 |
| sexMale | 0.01 | 0.00 | 411 | 1.47 | 0.1420 |

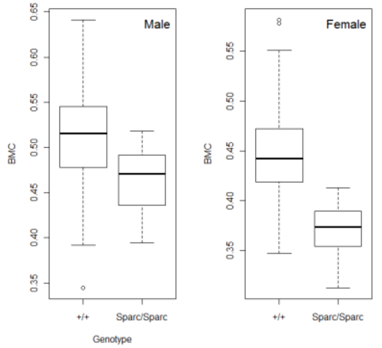


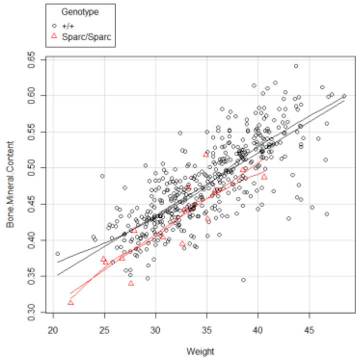


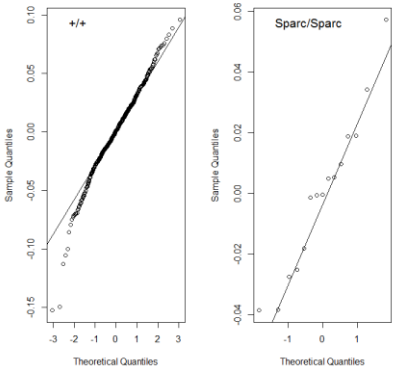


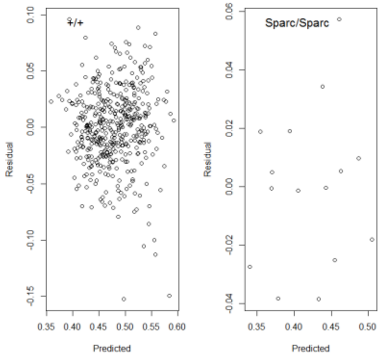


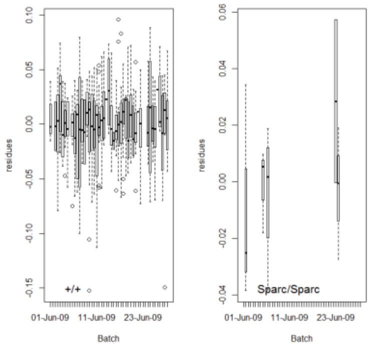


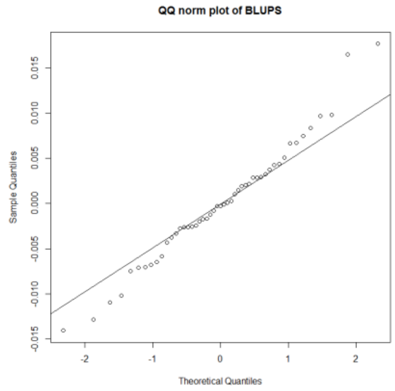

# Lean Mass:
# Top down modelling output

| Hypothesis | Model1 | Model 2 | Test | Estimation method | Test statistic value | *p*-value |
|---|---|---|---|---|---|---|
| Is batch significant? | Batch | No batch | LRT | REML | $\chi^2(0{:}1)=60.57$ | <0.0001 |
| Is variance homogenous? | Homogenous | Heterogeneous | LRT | REML | $\chi^2(2)=5.55$ | 0.0185 |
| Testing fixed effects – sex | | | Type 1 *F*-test | REML | F(1,411)=84.703 | <0.0001 |
| Testing fixed effects – weight | | | Type 1 *F*-test | REML | F(1,411)=363.67 | <0.0001 |
| Testing fixed effect – genotype*sex | | | Type 1 *F*-test | REML | F(1,47)=2.777 | 0.1022 |
| Testing treatment - Is genotype significant? | With genotype | Without genotype | LRT | ML | $\chi^2(2)=0.798$ | 0.3715 |

# Lean Mass: Final model values and diagnostics

Parameter estimates:

| | Value | Std.Error | DF | t-value | p-value |
|---|---|---|---|---|---|
| (Intercept) | 7.611139 | 0.588627 | 411 | 12.93034 | 0.0000 |
| GenotypeSparc/Sparc | -0.29144 | 0.33048 | 411 | -0.88186 | 0.3784 |
| sexMale | 1.640734 | 0.180809 | 411 | 9.074391 | 0.0000 |
| Weight | 0.34305 | 0.018081 | 411 | 18.97274 | 0.0000 |

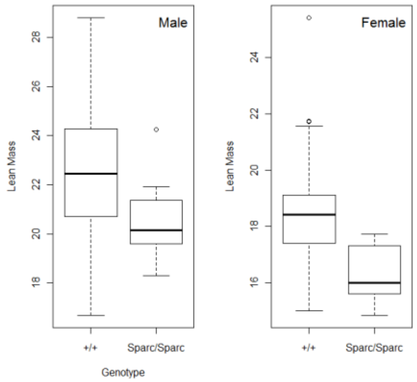


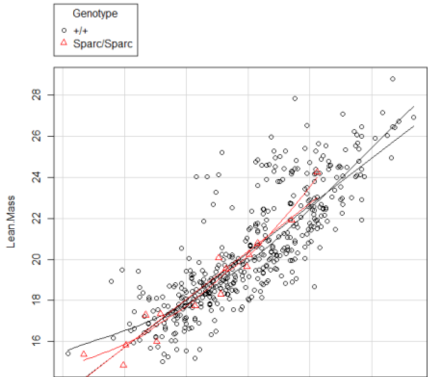


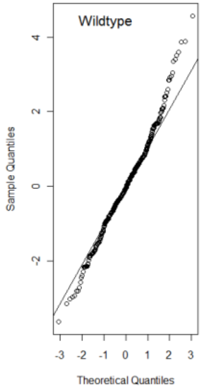


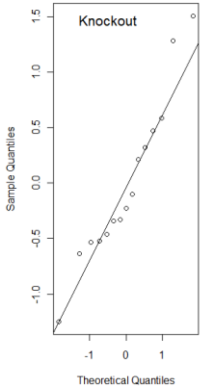


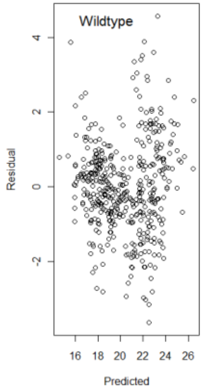


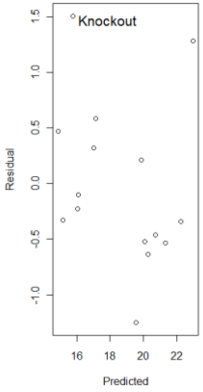


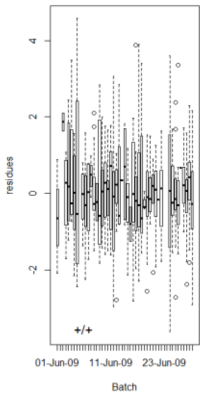


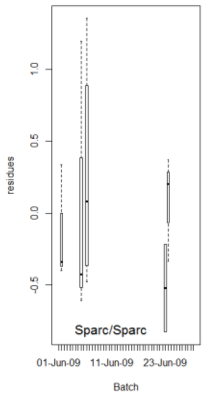


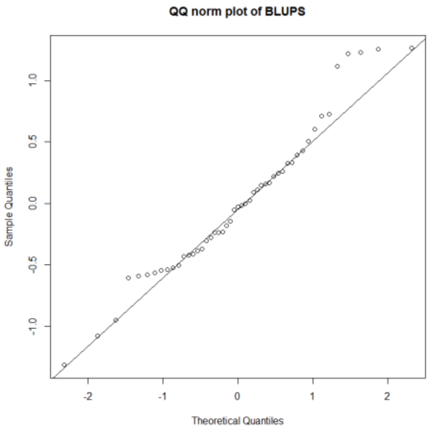

# Fat Mass: Final model values and diagnostics

| Hypothesis | Model1 | Model 2 | Test | Estimation method | Test statistic value | *p*-value |
|---|---|---|---|---|---|---|
| Is batch significant? | Batch | No batch | LRT | REML | $\chi^2(0:1)=52.79$ | <0.0001 |
| Is variance homogenous? | Homogenous | Heterogeneous | LRT | REML | $\chi^2(2)=7.515$ | 0.0061 |
| Testing fixed effects – sex | | | Type 1 F-test | REML | F(1,411)=84.754 | <0.0001 |
| Testing fixed effects – weight | | | Type 1 F-test | REML | F(1,411)=1309.60 | <0.0001 |
| Testing fixed effect – genotype*sex | | | Type 1 F-test | REML | F(1,47)= 0.744 | 0.3927 |
| Testing treatment - Is genotype significant? | With genotype | Without genotype | LRT | ML | $\chi^2(2)=1.554$ | 0.2125 |

# Fat Mass: Final model values and diagnostics

Parameter estimates:

| | Value | Std.Error | DF | t-value | p-value |
|---|---|---|---|---|---|
| (Intercept) | -8.58667 | 0.62306 | 411 | -13.7815 | 0.0000 |
| GenotypeSparc/Sparc | 0.393943 | 0.320326 | 411 | 1.22982 | 0.2195 |
| sexMale | -1.7669 | 0.192659 | 411 | -9.17113 | 0.0000 |
| Weight | 0.696697 | 0.019222 | 411 | 36.24525 | 0.0000 |

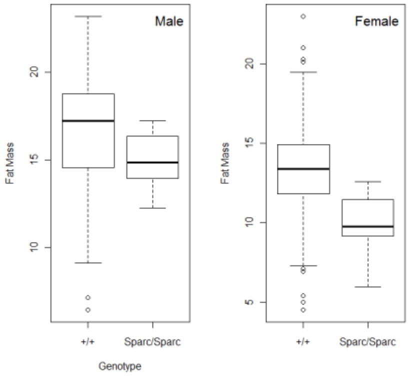


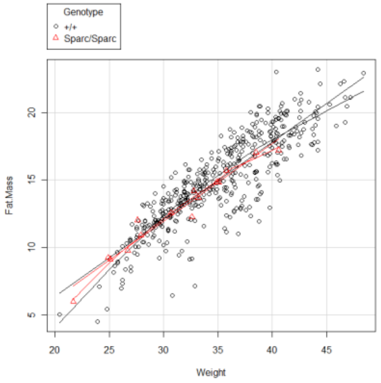


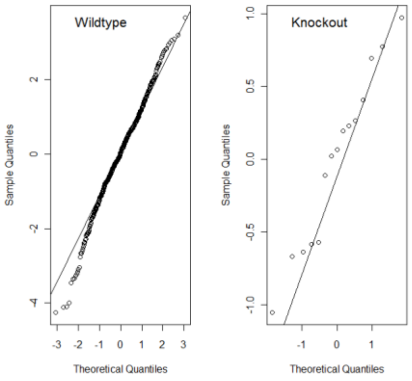


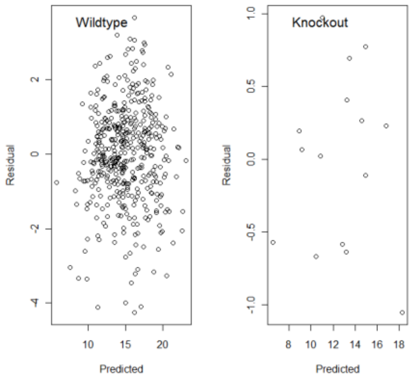


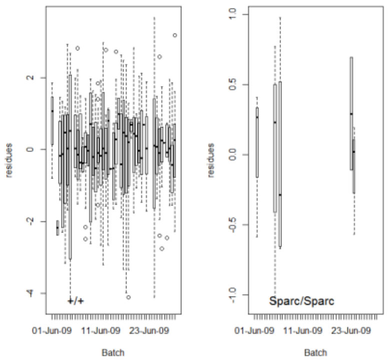


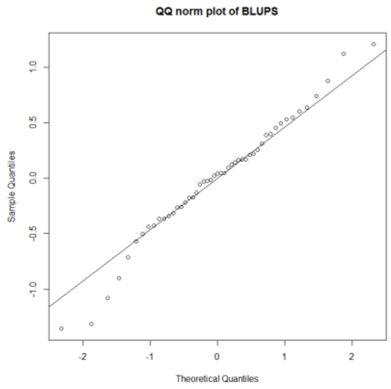

# Dependent variable: Fat Percentage (Fat %)

| Hypothesis | Model1 | Model 2 | Test | Estimation method | Test statistic value | *p*-value |
|---|---|---|---|---|---|---|
| Is batch significant? | Batch | No batch | LRT | REML | $\chi^2(0{:}1)=48.536$ | <.0001 |
| Is variance homogenous? | Homogenous variance | Heterogeneous | LRT | REML | $\chi^2(2)=3.3473$ | 0.0673 |
| Testing fixed effects – sex | | | Type 1 F-test | REML | F(1,411)=94.90 | <.0001 |
| Testing fixed effects – weight | | | Type 1 F-test | REML | F(1,411)=214.93 | <.0001 |
| Testing fixed effect – genotype*sex | | | Type 1 F-test | REML | F(1,47)=1.672 | 0.2023 |
| Is genotype significant? | With genotype | Without genotype | LRT | ML | $\chi^2(2)=0.6899$ | 0.4062 |

# Fat %: Final model values and diagnostics

Parameter estimates:

| | Value | Std.Error | DF | t-value | p-value |
|---|---|---|---|---|---|
| (Intercept) | 16.13209 | 1.784149 | 411 | 9.041899 | 0.0000 |
| GenotypeSparc/Sparc | 1.047678 | 1.275536 | 411 | 0.821363 | 0.4119 |
| sexMale | -5.30435 | 0.548967 | 411 | -9.66241 | 0.0000 |
| Weight | 0.808772 | 0.055044 | 411 | 14.69329 | 0.0000 |

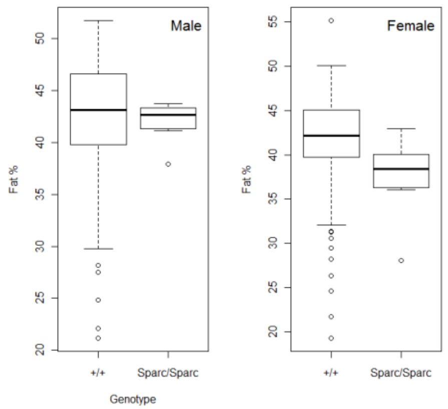


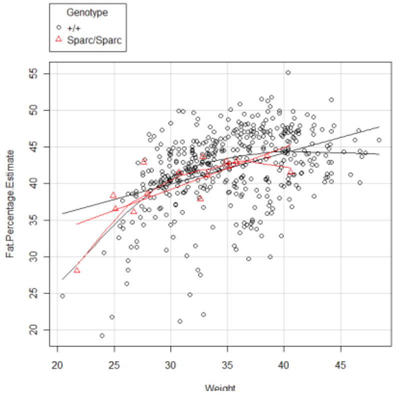


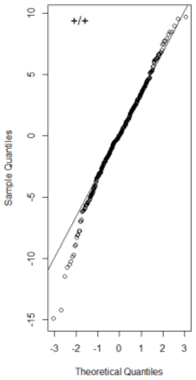


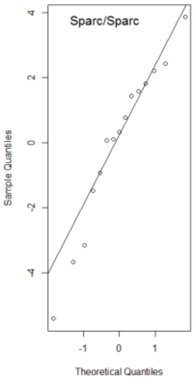


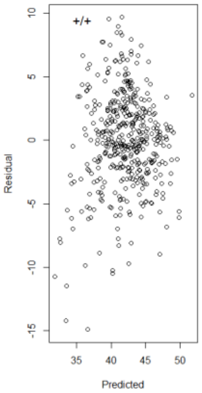


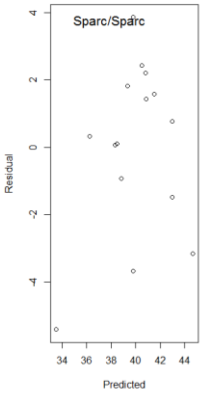


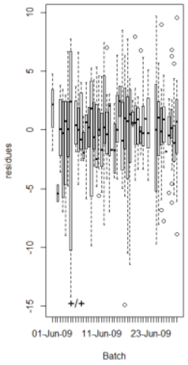


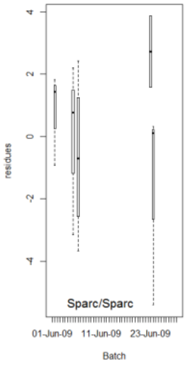


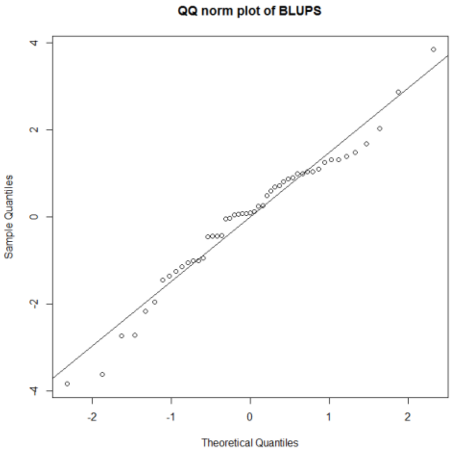

# Summary

| Variable | *p* value | Adjusted *p*' value | Genotype Estimate ± SE | Sex | Weight |
|---|---|---|---|---|---|
| Nose to tail length | 1.587e-7 | 7.52E-07 | Y↓ -0.325±0.060 | Y↑ | Y↑ |
| BMD | 7.048e-6 | 2.44E-05 | Y↓ -0.002±0.0005 | N | Y↑ |
| BMC | 0.03 | 0.0472 | Y↓ -0.02±0.01 | Y↑ | Y↑ |
| LM | 0.3715 | 0.4024 | N | Y↑ | Y↑ |
| FM | 0.2125 | 0.2569 | N | Y↓ | Y↑ |
| Fat % | 0.4062 | 0.4310 | N | Y↓ | Y↑ |

Y denotes a statistically significant effect and N indicates a non significant effect. The ↑ symbol indicates a positive estimated regression coefficient such that this effect leads to an increase in the dependent variable. Whilst, the ↓ symbol indicates a negative estimated regression coefficient such that this effect leads to a decrease in the dependent variable.
